# CTLA-4 blockade and interferon-α induce proinflammatory transcriptional changes in the tumor immune landscape that correlate with pathologic response in melanoma

**DOI:** 10.1371/journal.pone.0245287

**Published:** 2021-01-11

**Authors:** Arjun Khunger, Erin Piazza, Sarah Warren, Thomas H. Smith, Xing Ren, Andrew White, Nathan Elliott, Alessandra Cesano, Joseph M. Beechem, John M. Kirkwood, Ahmad A. Tarhini

**Affiliations:** 1 Department of Internal Medicine, Memorial Hospital West, Pembroke Pines, Florida, United States of America; 2 NanoString® Technologies, Inc., Seattle, Washington, United States of America; 3 ESSA Pharma, South San Francisco, California, United States of America; 4 UPMC Hillman Cancer Center, Pittsburgh, Pennsylvania, United States of America; 5 Department of Cutaneous Oncology and Immunology, H. Lee Moffitt Cancer Center and Research Institute, Tampa, Florida, United States of America; 6 University of Pittsburgh, Pittsburgh, Pennsylvania, United States of America; University of Nebraska Medical Center, UNITED STATES

## Abstract

Patients with locally/regionally advanced melanoma were treated with neoadjuvant combination immunotherapy with high-dose interferon α-2b (HDI) and ipilimumab in a phase I clinical trial. Tumor specimens were obtained prior to the initiation of neoadjuvant therapy, at the time of surgery and progression if available. In this study, gene expression profiles of tumor specimens (N = 27) were investigated using the NanoString nCounter® platform to evaluate associations with clinical outcomes (pathologic response, radiologic response, relapse-free survival (RFS), and overall survival (OS)) and define biomarkers associated with tumor response. The Tumor Inflammation Signature (TIS), an 18-gene signature that enriches for response to Programmed cell death protein 1 (PD-1) checkpoint blockade, was also evaluated for association with clinical response and survival. It was observed that neoadjuvant ipilimumab-HDI therapy demonstrated an upregulation of immune-related genes, chemokines, and transcription regulator genes involved in immune cell activation, function, or cell proliferation. Importantly, increased expression of baseline pro-inflammatory genes *CCL19*, *CD3D*, *CD8A*, *CD22*, *LY9*, *IL12RB1*, *C1S*, *C7*, *AMICA1*, *TIAM1*, *TIGIT*, *THY1* was associated with longer OS (*p* < 0.05). In addition, multiple genes that encode a component or a regulator of the extracellular matrix such as *MMP2* and *COL1A2* were identified post-treatment as being associated with longer RFS and OS. In all baseline tissues, high TIS scores were associated with longer OS (*p* = 0.0166). Also, downregulated expression of cell proliferation-related genes such as *CUL1*, *CCND1* and *AAMP* at baseline was associated with pathological and radiological response (unadjusted *p* < 0.01). In conclusion, we identified numerous genes that play roles in multiple biological pathways involved in immune activation, immune suppression and cell proliferation correlating with pathological/radiological responses following neoadjuvant immunotherapy highlighting the complexity of immune responses modulated by immunotherapy. Our observations suggest that TIS may be a useful biomarker for predicting survival outcomes with combination immunotherapy.

## Introduction

Stage III melanoma encompasses regional lymphatic and/or lymph node involvement, for which the current standard of care is surgery followed by adjuvant therapy. The first adjuvant therapy approved for melanoma was high dose interferon α-2b (HDI) which demonstrated reduced risk of relapse or death [1, 2]. More recently, adjuvant therapy with immune checkpoint blockade has demonstrated significant relapse-free survival (RFS) benefits, leading to US Food and Drug Administration (FDA) approval of single-agent ipilimumab (CTLA-4 blockade), nivolumab (PD-1 blockade) and pembrolizumab (PD-1 blockade) for adjuvant treatment of patients with high-risk stage III melanoma following complete resection [3–10]. In the phase III E1609 trial, ipilimumab was compared with HDI as adjuvant therapy in high risk stage III/IV melanoma patients, and ipilimumab at the 3 mg/kg dose was associated with improved overall survival when compared to HDI treatment [11–13]. For patients with resected, stage III BRAF mutated melanoma, the combination of dabrafenib and trametinib gained regulatory approval in adjuvant settings after it significantly improved RFS as compared to placebo [14].

In addition to the adjuvant use of immunotherapeutic agents, neoadjuvant therapy has emerged as a promising therapeutic option with the potential to improve surgical resectability, organ preservation, and overall survival, as well as to further reduce the risk of relapse in patients [15–17]. A number of neoadjuvant trials of molecularly-targeted agents and immunotherapies for local/regional metastases have shown improved clinical outcomes followed by adjuvant therapy [15, 17]. Efficacy of immunotherapy in the neoadjuvant setting has been assessed in multiple phase II/III trials of HDI or immune checkpoint inhibitor(s) [16, 18–21]. One of the advantages of neoadjuvant therapy as adopted in our study is providing access to biospecimens, allowing the evaluation of pathologic responses [17]. In addition, genomic and transcriptomic analyses of biospecimens provide new strategies to characterize the pro-inflammatory tumor microenvironment (TME) and correlate it with tumor response. For example, a prospective study by Weiss *et al*. demonstrated that pre-treatment biopsies of patients with metastatic melanoma that responded to IL-2 therapy displayed a pattern of immune activation [22]. In a retrospective study, a 4-gene immunotherapy panel of PD-L1, PD-L2, CD8D, and IRF1 demonstrated an association with clinical outcome in melanoma patients treated with pembrolizumab, nivolumab, or ipilimumab plus nivolumab [23]. Further, association of an IFN-inflammatory gene expression signature with clinical responses to pembrolizumab have been observed in melanoma patients [24, 25]. Recently, a gene-set scoring study has indicated that natural killer (NK) cell infiltration is also associated with an improved survival rate in metastatic cutaneous melanoma [26].

Previously, we conducted a clinical trial using combination immunotherapy with HDI and ipilimumab as neoadjuvant therapy in locally/regionally advanced operable melanoma (NCT01608594) [27]. The study was based on the rationale that patients with melanoma display strong Th2 polarized T cell responses that facilitates tumor progression and use of this combination therapy would synergistically induce a pro-inflammatory cytokine response (T_h_1 polarization), leading to increased T-cell infiltration and immune surveillance to control tumors [27]. It has been reported that in combination studies, presence of a T-cell-inflamed tumor microenvironment correlates with clinical efficacy of immunotherapies, including anti-PD-1 or IFN-α neoadjuvant therapy [20, 28–31], and T cell infiltrates are prognostic of outcome in patients with primary melanoma as well as melanoma metastatic to regional lymph nodes [28, 32, 33]. Thus, there is a critical need to better understand the predictive value of these immune activation signatures and develop biomarkers that classify patients who are most likely to derive clinical benefit from different forms of immunotherapy. For immunotherapy using ipilimumab and HDI, we were interested in defining potential resistance mechanisms and how we may build upon this regimen in designing future combinations.

In this study, our aim was to investigate potential biomarkers for response to this combined immunotherapy regimen, comparing gene expression in pre-treatment biopsies to specific response signatures in post-treatment patient samples. An additional goal of this study was to characterize the performance of the Tumor Inflammation Signature (TIS), an 18 gene expression signature that measures a peripherally suppressed adaptive immune response in the TME [25, 34]. TIS was previously shown to enrich for response to pembrolizumab in a variety of indications, but it has not been evaluated in the context of HDI plus ipilimumab to date [25]. In addition, cell typing signatures [35] and other research signatures were tested. Gene expression profiling was performed using three nCounter® PanCancer panels **(S1 Table)**; each of which contains 770 genes relevant to cancer progression, cancer pathways, and the immune response to the tumor. After accounting for overlap of genes between the panel, the samples were profiled with 1956 unique genes.

## Methods

### Patients

Patients with locally advanced, regionally advanced or recurrent melanoma were enrolled in this study (N = 30) and were treated with 3 or 10 mg/kg ipilimumab and HDI [27]. Inclusion criteria were patients above 18 years of age and clinically detectable locally and/or regionally advanced melanoma (cutaneous, mucosal or unknown primary) including (1) Tx or T1–4 and (2) N1b, or N2b, or N2c, or N3 and (3) M0 (American Joint Committee on Cancer, 7th edition). Patients were excluded if they met safety exclusion criteria related to CTLA4 blockade and interferon-α. Two doses of ipilimumab were given every 3 weeks, the first dose given 1–2 weeks after a baseline biopsy and prior to surgery. Two additional doses were administered after surgery 3 weeks apart, followed by up to 4 doses 12 weeks apart. IFN-α2b was administered concurrently with ipilimumab 20 MU/m²/day intravenously for 5 consecutive days/week for 4 weeks, followed by 10 MU/m²/d subcutaneously every other day, 3 times each week for 2 weeks prior to definitive surgery. After surgery, HDI was resumed at 10 MU/m²/d subcutaneously, every other day three times a week for 46 additional weeks. Of 30 patients enrolled, 2 were excluded due to disease stage. The study was initiated after approval from the institutional review board (IRB) and was conducted in accordance with the Declaration of Helsinki. Patients were enrolled between May 2013 and February 2016 at the University of Pittsburgh. A University of Pittsburgh IRB approved written informed consent (IRB#PRO12020161) was obtained from all patients.

### Clinical efficacy assessment

Clinical efficacy was assessed as tumor response evaluation per modified World Health Organization (mWHO) criteria using imaging studies conducted at baseline, 6–8 weeks after initiation of therapy (before surgery), and every 3 months thereafter. Based on best overall response as assessed pre-operatively, radiologic responses were categorized as complete response (CR), partial response (PR), stable disease (SD), or progressive disease (PD). Due to planned definitive surgery, responses were not possible to confirm radiologically based on mWHO criteria. Pathologic complete response (pCR) was assessed at the time of definitive surgery and defined as no visible malignant cells on hematoxylin and eosin staining by histological assessment. In addition, OS and RFS data were obtained for all patients.

### Tumor biopsies

Tumor biopsies were obtained at baseline (before initiating neoadjuvant therapy), at the time of surgery (resected tumor at 6–8 weeks following the initiation of neoadjuvant therapy), and at the time of progression (if available). Archival samples collected from primary tumors at the time of diagnosis were also available.

### Gene expression analysis

Formalin-fixed paraffin-embedded (FFPE) tissue samples were collected from 23 primary tumors, 22 baseline metastases, 25 post-treatment metastases, and 5 metastases from progressive disease. Total RNA was extracted using the Qiagen FFPE RNeasy kit. RNA concentration was measured by UV spectrophotometry and each sample was adjusted to 20 ng/μL. 100 ng total RNA from each sample was profiled with the NanoString nCounter® PanCancer Immune Profiling, PanCancer Pathways, and PanCancer Progression panels (**S1 Table**). Each panel was combined with a custom 30-gene Panel-Plus codeset to profile additional genes related to TIS, immune cell type profiling, and immunometabolism (**S1 Table**). Therefore, a total of 1,948 unique genes were profiled in this study. The data will be provided upon request from the corresponding author.

### Statistical methods

All data processing and figure generation was performed using R version 3.6.3.

#### Positive control probe normalization

Positive control normalization was used to reduce platform-associated sources of variation by normalizing count values based on the counts from six synthetic positive control targets that are included in each panel. Normalized counts were then log2 transformed.

#### Exclusion criteria

Each panel includes a set of housekeeping genes whose expression correlates with the quantity of input RNA. For each of the three panels, normalization factors were computed for each sample based on its relative geometric mean of the panel’s housekeeping genes. A housekeeping normalization factor > 8 would suggest a dramatically lower relative quantity of input RNA, thus any samples exceeding this threshold in any of the three panels were excluded from analysis. Six samples were excluded based on this criterion.

#### Merging panel expression data

Due to overlap in the targets covered by the three different panels used in this study, some genes had two, or, more rarely, three different expression values. This was especially true for housekeeping genes. These values were highly concordant, and are represented as their arithmetic means in the merged dataset.

#### Housekeeping gene normalization

After merging data from the three panels, housekeeping gene normalization was applied to correct for variability in sample input. This was performed using the union of the sets of housekeeping genes from the three different panels, which totaled 50 genes.

#### Signature score calculation

Gene expression signature scores were calculated as weighted linear averages of their constituent genes, using weights that are proprietary to NanoString Technologies.

#### Unsupervised clustering and dimensionality reduction

The heatmaps of signature scores were generated using the R package *pheatmap* [36] with row and column clustering by Euclidian distance. Principal component analysis (PCA) plots were generated using the base R *stats* package.

#### Differential expression analysis

For analysis of differential gene expression or signature scores, normalized gene counts or signature scores were fitted to the independent variable (i.e. pre- vs. post-treatment, responder vs. non-responder, baseline metastasis vs. primary tumor) with a linear model using the base R *stats* package. The log2 fold-change, Wald-Type 95% confidence interval and *p*-value were calculated for each gene and signature. For the analysis of therapy-induced changes in gene expression, *p*-values were adjusted by the Benjamini-Hochberg Procedure.

#### Survival analysis

Associations between gene expression or signature scores and OS or RFS was assessed by fitting a univariate Cox proportional hazards model for each gene or signature, with *p*-values derived from the log-rank test [37]. For analysis of the association between TIS and OS or RFS time, TIS scores were dichotomized into high and low groups based on the median TIS score across all samples. Kaplan-Meier curves were used to visualize OS or RFS times for patients in the high and low strata. The reported *p*-values are from the log-rank tests from the Cox proportional hazards model fitted to the data.

## Results

### Patient characteristics and clinical efficacy

**S2 Table** summarizes demographics and baseline disease characteristics of the patients who participated in the clinical trial. Among 30 patients enrolled, 27 had adequate tumor tissue for gene expression profiling analysis. Among 27 patients, 11 patients relapsed, of whom 5 died. Based on mWHO criteria, radiological response was observed in 10 patients (37%,) while 10 showed stable disease and 7 patients showed radiologic progression of disease. In this cohort, 8 patients (29.6%) achieved pathologic complete response.

### Clustering of gene expression signatures in pre-treatment (baseline) vs. post-treatment samples

Gene expression signatures were analyzed and compared between FFPE tumor samples obtained prior to neoadjuvant therapy (baseline) and resected tumor tissues at the time of surgery, normally 6–8 weeks following the initiation of neoadjuvant therapy. These analyses of 48 samples (24 patient-matched pre- and post-treatment samples) demonstrated some clustering of gene expression signatures specific to baseline vs. post-neoadjuvant treatment as shown in heatmaps (**S1 Fig**).

### Comparison of gene expression between primary tumor and baseline metastasis samples

To characterize and compare primary tumors to baseline metastasis samples, the expression patterns of individual genes as well as gene signatures were analyzed. In these tissue samples (n = 13), only four genes *ID4*, *HMGCR*, *ROCK1*, and *CPT1A* were differentially expressed (unadjusted *p* < 0.01, **S2A Fig**), and no gene expression signatures were significantly different between primary tumor and baseline metastasis samples (**S2B Fig**). Due to the minimal differences between primary tumor and baseline metastasis tissues, baseline metastasis samples were used as a baseline sample, if possible, otherwise primary tumor samples were used in this study.

### Association of baseline gene expression with pathologic response

**Fig 1:**
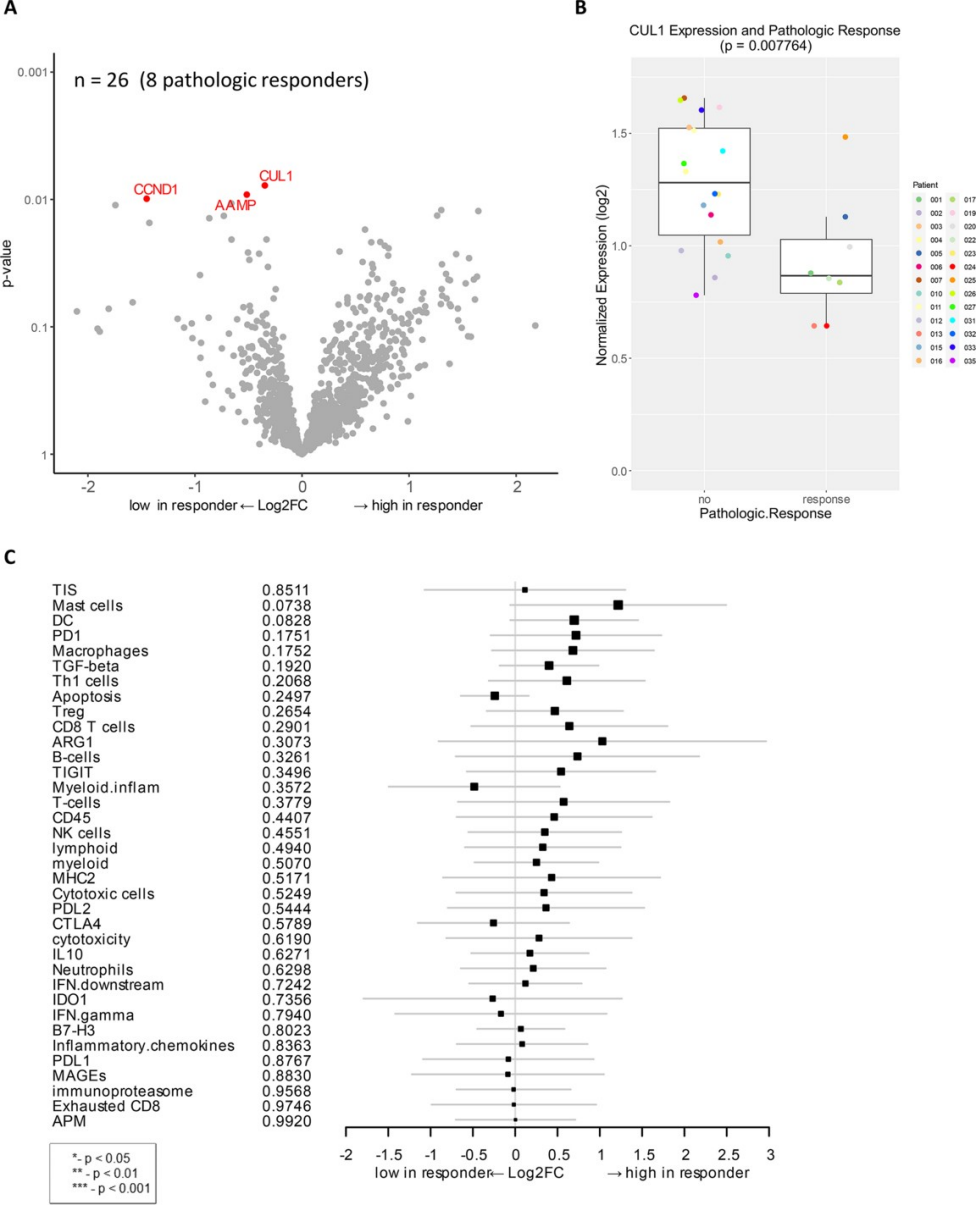
Genes associated with pathologic response to combination immunotherapy of HDI and ipilimumab as a neoadjuvant therapy. **A**) Volcano plot of unadjusted *p*-value vs. log2-fold change of the differential expression in patients who demonstrated pathologic response (unadjusted *p* < 0.01). Among 27 evaluable patients, 26 available samples (8 pathologic responders) were analyzed. **B**) Boxplot of *CUL1* expression and pathologic response. **C**) Forest plot of difference of gene signature scores between low expression and high expression in pathologic responders. The position of the squared dots denotes the difference of score, and the size denotes the statistical significance. The horizontal lines are the Wald-type confidence intervals. There was no significant *p*-value in this analysis.

Differential expression analysis of individual genes was performed on available baseline samples from 8 patients with pCR and 18 patients with residual tumor. The *CUL1* gene was significantly downregulated in baseline samples of patients with pCR as compared to those with residual tumor (unadjusted *p* < 0.01, **Fig 1A and 1B**). CUL1 is a scaffold protein in the SCF E3 ubiquitin ligase complex that mediate the ubiquitination of proteins involved in cell proliferation in melanoma [38]. Other genes that were significantly downregulated in patients with pCR were *CCND1* and *AAMP* (unadjusted *p* < 0.01), which are also linked to cell proliferation and migration (**Fig 1A**). *CCND1* encodes the cyclin D1 protein, which is complexed with cyclin-dependent kinases (CDKs) and regulates the cell cycle to induce cell migration and angiogenesis, linked to melanoma and metastatic progression [39, 40]. AAMP (angio-associated migratory cell protein) belongs to the immunoglobulin superfamily and has a function in cell migration and angiogenesis in breast cancer and non-small cell lung cancer [41, 42].

To determine if there are more robust indicators of biological activity, gene signatures rather than individual genes were analyzed in the cohort using previously developed gene signatures that describe TIS, cell typing, and other particular biological pathways [25, 34, 35]. Correlation analysis of gene expression signature to pathologic response showed some trends but were not statistically significant (**Fig 1C**).

### Association of baseline gene expression with radiologic response

**Fig 2:**
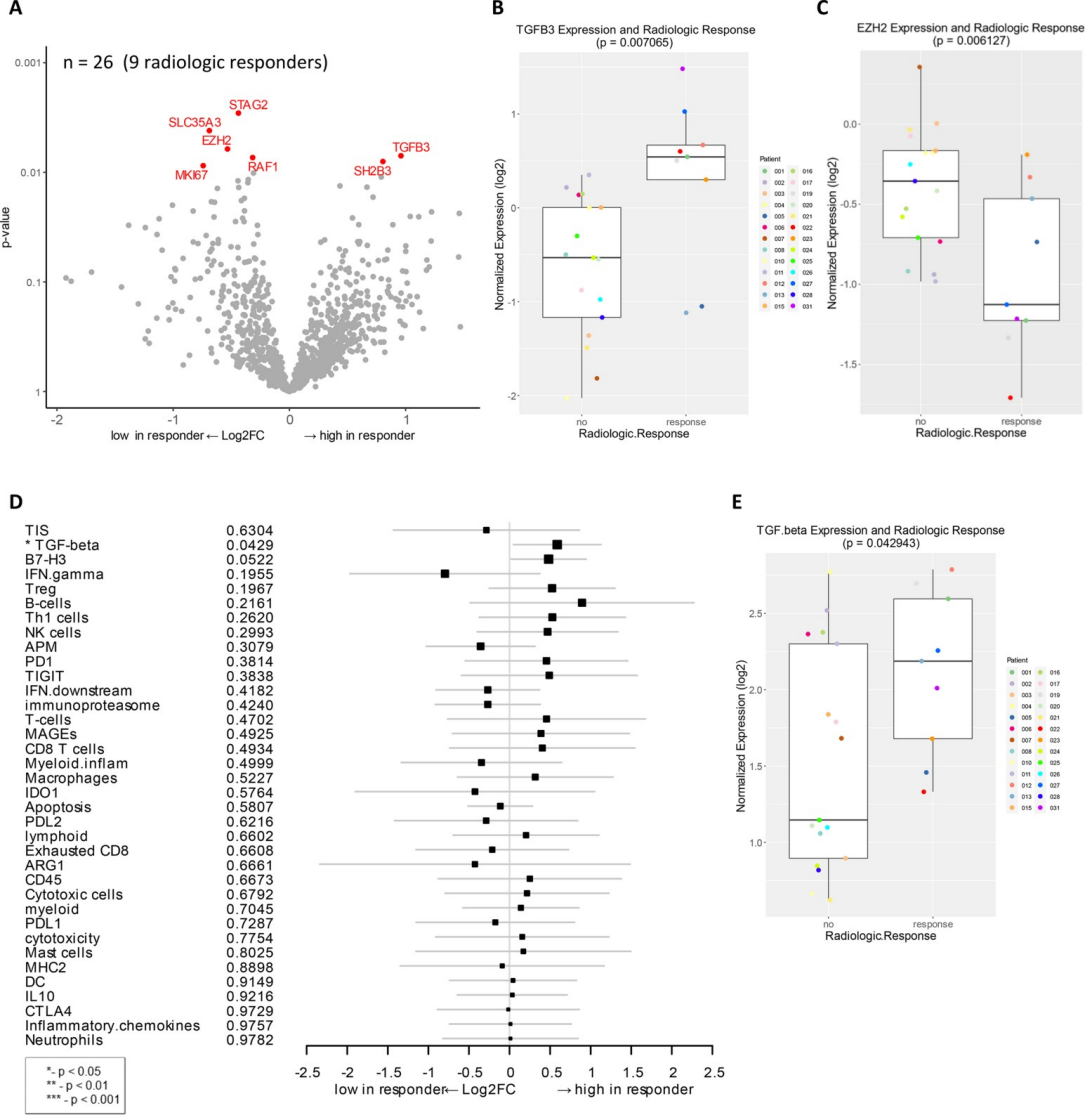
Genes and signatures associated with radiologic response in patients treated with combination immunotherapy of HDI and ipilimumab as a neoadjuvant therapy. **A**) Volcano plot of unadjusted *p*-value vs. log2-fold change of the differential expression in patients who demonstrated radiologic response (unadjusted *p* < 0.01). Among 27 evaluable patients, 26 samples (9 radiologic responders) were available for analysis. **B**) Boxplot of *TGFB3* expression and radiologic response. **C**) Boxplot of *EZH2* expression and radiologic response. **D**) Forest plot of difference of gene signature scores between low expression and high expression in radiologic responders. The position of the squared dots denotes the difference of score, and the size denotes the statistical significance. The horizontal lines are the Wald-type confidence intervals. The * sign denotes the significance of *p*-value (< 0.05*). **E**) Boxplot of *TGF-beta* signature score and radiologic response.

Next, we analyzed the correlation of the baseline gene expression with radiographic response by comparing radiologic responders (PR+CR) to patients with stable or progressive disease (SD+PD). *TGFB3* and *SH2B3* were highly expressed in radiologic responders (unadjusted *p* < 0.01, **Fig 2A and 2B**). *TGFB3* encodes transforming growth factor-β3 (TGF-β3), which is a multifunctional cytokine expressed in melanoma cells [43]. *SH2B3* encodes a lymphocyte adaptor, reported as a tumor suppressor gene in leukemia [44, 45]. Several genes that were downregulated in radiologic responders, compared to non-responders, include *EZH2*, *STAG2*, *SLC35A3*, *RAF1*, and *MKI67* (**Fig 2A and 2C**). EZH2 is an epigenetic-modifying histone methyltransferase, known to regulate melanoma growth and metastasis by silencing tumor suppressors and also induce resistance to immunotherapy [46–48]. Moreover, gene signature analysis of this cohort identified a correlation of TGF-β expression to radiologic response (*p* < 0.05, **Fig 2D and 2E**).

### Association of baseline gene expression with survival

**Fig 3:**
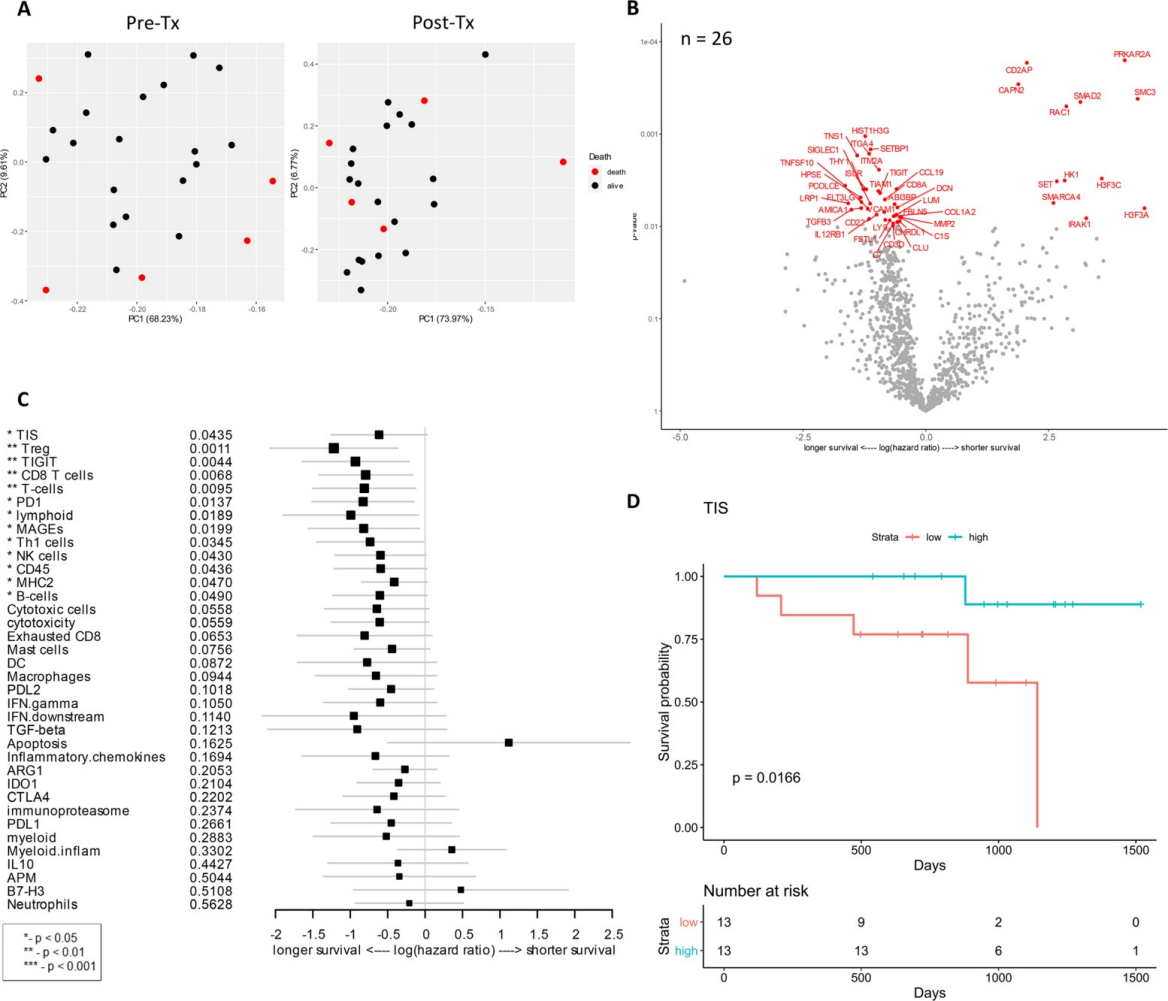
Genes and signatures associated with overall survival of patients treated with combination immunotherapy of HDI and ipilimumab as a neoadjuvant therapy. **A**) Principal component analysis was performed to look for distinct gene expression patterns in baseline vs. post-treatment samples by OS. **B**) Volcano plot of unadjusted *p*-value vs. log-hazard ratio of the differential expression associated with overall survival of treated patients (unadjusted p < 0.01). Among 27 evaluable patients, 26 samples were available for this analysis. **C**) Forest plot of difference of gene signature scores based on hazard ratio between longer survival and shorter survival. The position of the squared dots denotes the difference of score, and the size denotes the statistical significance. The horizontal lines are the Wald-type confidence intervals. The * sign denotes the significance of *p*-value (< 0.01**, < 0.05*). **D**) The Kaplan-Meier curves of TIS score groups for available samples (n = 26). Patients are stratified into “high” and “low” groups based on the TIS score 50% quantile. The Kaplan-Meier curves show that the high TIS score group has higher survival rate than the low TIS score group. The survival time was fit to TIS score group (high vs. low) with Cox proportional hazard model (*p*-value = 0.0116).

Among 27 evaluable patients, 22 patients survived with the median follow-up of 32 months while 5 patients died. Principal component analysis was performed to look for distinct gene expression patterns in baseline vs. post-treatment samples by outcome, which indicated that pre-treatment samples were relatively distinct when grouped by OS (**Fig 3A**). Analysis of differential gene expression associated with OS identified numerous genes (**Fig 3B**) compared to pathologic or radiologic response (**Figs 1 and 2**). The correlations of gene expression and signatures to OS were plotted with respect to their individual hazard ratios (**Fig 3B and 3C**). Many genes differentiated the survivors from non-surviving patients, encompassing aspects of tumor, immune, and stromal gene expression. Among the genes associated with longer survival included *TNFSF10*, also known as TRAIL, *TNS1* (Tensin 1; localizes to focal adhesions), *FLT3LG* (controls the development of dendritic cells), and TGFB3 (TGF-β3) (unadjusted *p* < 0.01, **Fig 3B**). In colorectal cancer, *TNS1* is identified as a potential biomarker for tumor cell proliferation and invasion [49]. TGFB3 was also associated with radiologic response and highly expressed in radiologic responders as shown in **Fig 2**. Immune-related genes whose expression was associated with longer OS were *CCL19*, *CD3D*, *CD8A*, *CD22*, *LY9*, *IL12RB1*, *C1S*, *C7*, *AMICA1*, *TIAM1*, *TIGIT*, and *THY1*. In addition, expression of extracellular matrix-related proteins and molecules (*COL1A2*, *MMP2*, *PCOLCE*, *DCN*, *FBLN5*, *SIGLEC1*, *HPSE*) was correlated with longer OS (**Fig 3B**). *COL1A2* encodes a type I collagen, one of main extracellular matrix proteins, which is controlled by its degrading matrix metalloproteinases (MMPs) and implicated in cancer development and progression [50, 51].

Genes that were associated with shorter OS (*SMC3*, *SMARCA4*, *SET*, *H3F3C*, *H3F3A*, *CD2AP*, *RAC1*, *IRAK1*, *PRKAR2A*) are also known to have several biological functions. For example, SMC3, SMARCA4, SET, H3F3C, and H3F3A (**Fig 3B**) are involved in regulation of gene expression by controlling the chromosome, chromatin, or histones. CD2AP (CD2 associated protein) and RAC1 (Rac Family Small GTPase 1) play a role in actin cytoskeleton remodeling and cell growth. IRAK1 (IL-1 receptor-associated kinase 1) is responsible for IL-1-induced upregulation of the transcription factor NF-κB. Expression of *PRKAR2A* (encoding the protein kinase cAMP-dependent type II regulatory subunit alpha) as well as the signal transducer SMAD2, which modulates multiple signaling pathways including TGF-β pathway, was strongly associated with unfavorable outcome (**Fig 3B**).

As observed in gene expression analysis, many genes and signatures correlated with longer OS, including CD8 T cell, T cells, lymphoid, T_h_1 cells, and CD45 (lymphocyte common antigen) signatures which are related to T cell/lymphoid activation and functions (*p* < 0.05, **Fig 3C**). Other genes and signatures that were correlated with longer OS include Treg, TIGIT (T cell immunoreceptor with Ig and ITIM domains), PD-1, MAGEs (melanoma antigen genes), NK cells, MHC2, B cells, and tumor inflammation signature (TIS) (*p* < 0.05, **Fig 3C**). This is concordant with the previously reported prognostic abilities of TIS in metastatic melanoma [34]. As tumor TIS measures a suppressed immune response in the tumor, including antigen presenting cell and T cell presence, IFN-γ related biology, and T cell suppression, we further investigated the changes in TIS for individual patients over the course of therapy. TIS was calculated for all available pre-treatment biopsies, which were then stratified into low and high TIS groups based on scoring of patient populations. In this cohort, a high TIS score was associated with extended OS with a *p*-value of 0.0166 (**Fig 3D**).

We further identified genes that were significantly associated with both OS and pathologic response. With a significance threshold of *p* < 0.05, there were 24 genes positively associated with each outcome: *ACVRL1*, *BAP1*, *CCND1*, *CD36*, *CHRDL1*, *CUL1*, *DCN*, *DPT*, *FBLN1*, *ISLR*, *LEPR*, *LUM*, *MEOX2*, *MIF*, *MMRN2*, *PDGFRA*, *PRKAR2A*, *PTK2*, *SH2B3*, *SLC3A2*, *TIE1*, *TNFSF10*, *VASH1*, and *WEE1*.

### Association of baseline gene expression with relapse-free survival

Median follow-up for 16 patients who have not relapsed was 32 months. In this cohort, differentially expressed genes and signatures were assessed for a correlation with RFS (**S3A and S3B Fig**). Expression of *TNS1 and TNFSF13B* in samples from longer RFS (**S3A Fig**) supports the gene expression data of *TNS1* and *TNFSF10*, another TNF superfamily member, being correlated with longer OS (**Fig 3B**). Notably, there was a trend of extracellular matrix-related proteins (*MMP2*, *COL1A2*, *PCOLCE*, *DCN*) expression with longer RFS (**S3A Fig**) as observed with longer OS (**Fig 3**).

Increased expression of *SMAD2*, *SMAD4*, and *SETD2* were strongly associated with shorter RFS (unadjusted *p* < 0.01, **S3A Fig**. *SMAD* proteins are involved in signal transduction of the TGF-β superfamily, and it should be noted that SMAD2 was associated with shorter OS (**Fig 3B**). The other gene *SETD2* (SET domain containing 2) encodes a histone lysine methyltransferase that plays a role in chromatin structure modulation. Expression of two immune-related genes *CD2AP* (CD2-associated protein) and *CD46* (complement regulatory protein) showed a statistically significant correlation to shorter RFS (**S3A Fig**).

Gene expression and signature analysis identified an association of MAGEs, CD8 T-cells, and PD-1 with longer RFS (*p* < 0.05, **S3B Fig**. All of these were also correlated to OS (**Fig 3C**), suggesting the importance of these biological functions in patient survival. As analyzed in the OS cohort, the change in TIS in the RFS cohort was also followed up over the course of therapy. For all available pre-treatment biopsies, stratified TIS scores demonstrated a trend of being associated with RFS, but it was not statistically significant (*p*-value = 0.0917, **S3C Fig**).

### Combination therapy-induced changes in gene expression

**Fig 4:**
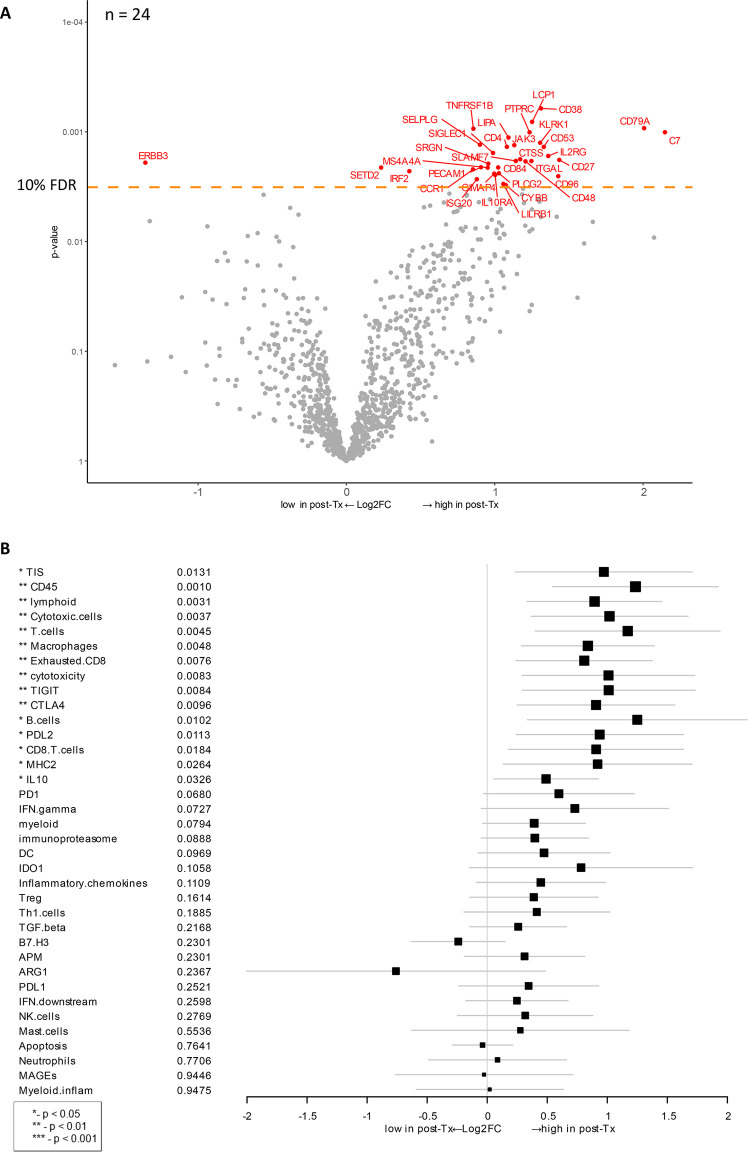
Therapy-induced changes after HDI and ipilimumab treatment vs. baseline. **A**) Volcano plot of unadjusted *p*-value vs. log2-fold change of the differential expression after treatment. Dots corresponding to genes that are significant with an FDR (false discovery rate) less than 10%. This analysis includes data from 24 patients (n = 24) for whom both baseline and post-treatment samples were available. **B**) Forest plot of difference of gene signature scores after treatment. The position of the squared dots denotes the difference of score, and the size denotes the statistical significance. The horizontal lines are the Wald-type confidence intervals. The * sign denotes the significance of *p*-value (< 0.01**, < 0.05*).

To investigate gene expression changes in response to ipilimumab/HDI combination therapy, we compared the gene expression data between baseline biopsy and post-treatment samples from patients for whom data from both time points were available (n = 24 patients, e.g., 48 samples). After treatment, expression of C7 and CD79A was most dramatically increased (**Fig 4A**). It is intriguing that C7 expression was also associated with longer OS (**Fig 3B**). C7 encodes a complement protein C7 that forms a membrane attack complex with other complement components and is recognized as a potential tumor suppressor [52]. CD79A encodes the B-cell antigen receptor complex-associated protein, which plays diverse roles in B cell development and function. Other biological groups of genes that were upregulated post-therapy include immune-related genes (*KLRK1*, *IL2RG*, *IL10RA*, *JAK3*, *CD4*, *CD27*, *CD48*, *CD84*, *CD96*, *PTPRC*, *LCP1*, *ITGAL*, *SELPLG*, *SLAMF7*, *PECAM1*, *CCR1*, *ISG20*, *LILRB1*, *IRF2*), transcription regulators (*SETD2*, *IRF2*), and TNF-α function (*TNFRSF1B*). After therapy, only *ERBB3* (Erb-B2 Receptor Tyrosine Kinase 3) gene was significantly downregulated based on a cutoff at 10% FDR (false discovery rate). This gene encodes a tyrosine kinase for epidermal growth factor receptors (EGFRs), which play a role in development of various types of cancers [53, 54].

Analysis of therapy-induced changes in gene expression signatures detected a significant increase (*p* < 0.05) across multiple genes and signatures related to an activation of T-cell activity, including lymphoid, MHC2, cytotoxicity, B-cells, CD45, CD8 T-cells, cytotoxic cells, exhausted CD8, macrophages, T-cells, CTLA-4, IL-10, PD-L2, TIGIT, as well as TIS (**Fig 4B**). High expression of these genes and signatures indicates an overall increase in immune activity and lymphocyte infiltration.

### Changes in post-treatment gene expression that correlate to pathologic response

**Fig 5:**
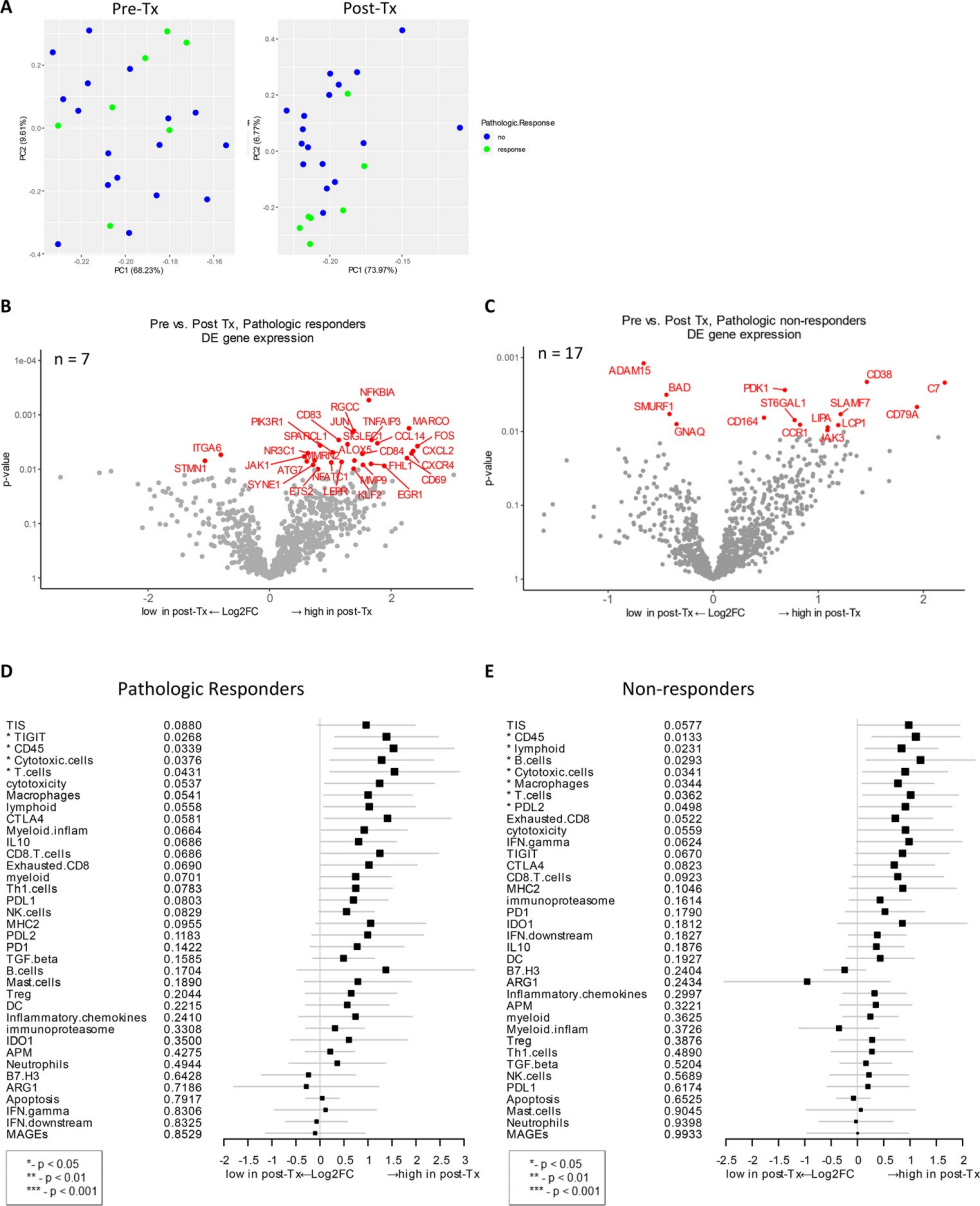
Post-treatment gene expression changes that correlate with pathologic response. **A**) Principal component analysis was performed to look for distinct gene expression patterns in baseline vs. post-treatment samples. **B**) Volcano plot of unadjusted *p*-value vs. log2-fold change of the differential expression after treatment, which associate with pathologic response (unadjusted *p* < 0.01). This analysis only includes data from 7 patients (n = 7) who exhibited pathologic response and for whom both baseline and post-treatment samples were available **C**) Analysis as as in Fig 5B, but only includes data from 17 patients (n = 17) who did not exhibit pathologic response and for whom both baseline and post-treatment samples were available (unadjusted *p* < 0.01). **D and E**) Forest plot of difference of gene signature scores post-treatment in pathologic responders (**D**) and non-responders (**E**). The position of the squared dots denotes the difference of score, and the size denotes the statistical significance. The horizontal lines are the Wald-type confidence intervals. The * sign denotes the significance of *p*-value (< 0.05*).

The gene expression change between baseline and post-treatment biopsy from pCR vs. those with residual tumor was examined to investigate if any changes correlated with pathologic response. Principal component analysis revealed that post-treatment samples grouped by pathologic response were most distinct from others outcome groups (**Fig 5A**). In patients with pCR, *NFKBIA* (NF-κB inhibitor α), *TNFAIP3* (TNF-α induced protein 3), and *MARCO* (macrophage pattern recognition scavenger receptor) were highly expressed after treatment (unadjusted *p* < 0.01, **Fig 5B**). Both NFKBIA and TNFAIP3 proteins inhibit NF-κB transcription factor activity to regulate genes responsible for innate and adaptive immune response. MARCO gene expression is normally associated with immunosuppressive pathways, enabling the establishment and persistence of solid tumors as well as metastasis in cancer [55, 56]. Other highly expressed genes post-therapy that are immune-related and correlated to pCR were *CCL14*, *CXCL2*, *CXCL4*, *NFATC1*, *JAK1*, and *CD84* (**Fig 5B**). In addition to these immune-related genes, other genes detected in post-therapy samples from patients with pCR play important role in cell cycle (*RGCC*), autophagy (*ATG7*), transcriptional regulation (*JUN*, *ETS2*, *NFATC1*, *KLF2*, *EGR1*), and extracellular matrix (*MMP9*, *SIGLEC1*). Another upregulated gene corresponding to therapy was *FOS* proto-oncogene, which regulates cell proliferation, differentiation, and transformation, in some cases, associated with apoptotic cell death.

Among pathologic non-responders, one of the differentially expressed genes after treatment was *SLAMF7* (signaling lymphocytic activation molecule [SLAM] family member 7) (unadjusted *p* < 0.01, **Fig 5C**). SLAMF7 is a surface marker of NK cells, T cells, and B cells and is involved in the regulation of NK cell functions in addition to other innate and adaptive immune response functions [57–59]. Other immune-related genes that were highly expressed post-therapy in non-responders include *CD79A*, *CD164*, *C7*, *CCR1*, and *JAK3*. CD164 (sialomucin) is involved in the regulation of cell proliferation and apoptosis, and the overexpression is detected in cancer patients [60]. Genes that were significantly downregulated after therapy were *ITGA6* and *STMN1* in pathologic responders; *ADAM15*, *BAD*, *SMURF1*, and *GNAQ* in non-responders (**Fig 5B and 5C**).

Next, treatment-induced gene expression changes that associated with pCR were analyzed for gene signatures. Four gene signatures, TIGIT, CD45, cytotoxic cells, and T cells, were highly expressed in patients who responded to therapy (*p* < 0.05, **Fig 5D**). In non-responders, multiple gene signatures including lymphoid, B cells, cytotoxic cells, macrophages, T-cells, CD45 and PD-L2 were increased after treatment (*p* < 0.05, **Fig 5E**). When these signatures were compared between these two cohorts, TIGIT was significantly highly expressed in pathologic responders while lymphoid, B cells, macrophages, and PD-L2 signatures were high in non-responders (**Fig 5D and 5E**).

### Changes in post-treatment gene expression that correlate to radiologic response

Lastly, changes in post-treatment gene expression were evaluated between samples available from radiologic responders (n = 8) vs. non-responders (n = 16). In radiologic responders, more than 10 genes such as *NFKBIA*, *MARCO*, *CHIT1* (chitotriosidase 1), and *TANK* (TRAF family member associated NF-κB activator) demonstrated increased expression that were associated with radiologic response (unadjusted *p* < 0.01, **S4A Fig**). Both *NFKBIA*, and *TANK* are involved in NF-κB activation, and common polymorphisms in NFKBIA is associated with susceptibility to cancer [61–63]. Chitinase is secreted by activated human macrophages, implicated in several types of cancers and inflammation [64]. Differential high expression of *MARCO*, *NFKBIA*, *RGCC*, *CCL14*, *CXCL2*, *PIK3R1*, and *ETS2* was correlated to both radiologic response and pathologic response to therapy (**Figs 5B and S4A**).

In tissue samples from patients with SD or PD, *IL2RG* (IL-2 receptor subunit gamma) was differentially expressed after treatment, which was previously identified to contribute in pancreatic cancer growth [65]. In post-therapy samples, increased expression of *SLAMF7*, *CD38*, *C7*, *and CD79A* was associated with lack of radiologic response (**S4A Fig**), and increased expression of these genes was also associated with pathologic non-responders (**Fig 5C**). Other highly expressed genes in non-responders after treatment were *CD22*, *CD27*, *CD53*, *ST6GAL1*, *JAK3*, *ICAM3*, *LCP1*, *RAC2*, *LILRB1*, and *ADA*; all these genes play a role in immunity or immune-regulation. Correlated to radiologic response, a few genes that were significantly downregulated after therapy: *PCNA* and *MAD2L2* in radiologic responders, *ADAM15* and *EPS8L1* in non-responders (**S4A and S4B Fig**). Post-therapy downregulation of ADAM15 was seen in pathologic non-responders as well (**Fig 5C**).

In evaluation of gene and signature expression in radiologic responders, only PD-L2 was statistically associated with radiologic response (*p* < 0.05, **S4C Fig**). In radiologic non-responders, B cells, CD45, lymphoid, T cells, cytotoxic cells, TIGIT, cytotoxicity, CTLA-4, and macrophages were highly expressed compared to responding patients (*p* < 0.05, **S4D Fig**). These data indicate overall increase in immune activity and lymphocyte infiltration post-treatment.

## Discussion

In this study, we characterized gene expression profiles of patients with locally advanced/regionally melanoma who underwent neoadjuvant ipilimumab and HDI combination treatment. The gene expression data were interpreted using PanCancer analysis to identify the association between gene expression at baseline and after treatment with response, either pathologic or radiologic. Special consideration was given to the performance of the TIS to characterize response and survival of patients. Schematic of experimental strategy and summary of results in this manuscript are shown in S5 Fig. Gene expression and signature analysis revealed significant changes in a number of genes involved in immune signaling pathways. Further, in baseline samples, lower expression of genes that control cell proliferation and migration in tumors were associated with pCR (**Fig 1A and 1B**). Analysis of correlation to radiologic response also revealed downregulated expression of genes involved in cell proliferation, cancer progression and recurrence (**Fig 2A and 2C**).

As expected, high expression of a number of pro-inflammatory genes at baseline was associated with a benefit in patient outcome. Increased expression of *CCL19*, *CD3D*, *CD8A*, *CD22*, *LY9*, *IL12RB1*, *C1S*, *C7*, *AMICA1*, *TIAM1*, *TIGIT*, *THY1* was associated with longer OS (*p* < 0.05) (**Fig 3B**). CCL19 gene codes a chemotactic factor that strongly attracts naïve T cells and DCs [66]. Also, CCL19 is a potent inducer of T-cell proliferation and induction of T helper (Th) 1 rather than Th2 responses [67]. More recently, CAR T cells secreting CCL19 are being evaluated to potentially increase tumor infiltration rate of dendritic cells and T cells, and improve anti-tumor immunity [68]. In contrast, it was intriguing to observe that higher levels of TIGIT, an inhibitory immune checkpoint receptor was associated with longer OS (Fig 3B).

In terms of radiologic response, upregulation of genes (*SH2B3* and *TGFB3*) as well as TGF-β signature demonstrated a significant correlation to response (**Fig 2A, 2B, 2D and 2E**). *SH2B3* is known as a tumor suppressor in leukemia [44, 45], supporting the relevancy of this gene to clinical benefit. The association of upregulated *TGFB3* gene expression and TGF-β signature with radiologic response was unexpected considering that activation of the TGF-β pathway normally leads to melanoma progression and metastasis [69, 70]. Moreover, *TGFB3* was correlated to longer OS (**Fig 3B**). On the other hand, increased expression of *SMAD2* and/or *SMAD4* that belong to the TGF-β cascade was strongly associated with shorter RFS and OS (**Figs 3B and S3A**). It is known that TGF-β plays a complex role during carcinogenesis by either acting as a tumor suppressor or as an oncogene in a context-dependent manner, as well as having known roles in the immune system that compound any activity already present within the tumor [71]. Based on these data, we hypothesize that, in complex ways, ipilimumab and HDI therapy influences the expression of genes in the TGF-β pathway in responders compared to non-responders; therefore, these genes may be biomarkers of clinical response and survival. Further studies are warranted to examine this hypothesis.

Another biological category of genes that correlated to patient survival was extracellular matrix-related proteins and molecules. Increased expression of *COL1A2*, *MMP2*, *PCOLCE*, *DCN*, *FBLN5*, *SIGLEC1*, and *HPSE* was associated with longer OS (**Fig 3B**), and *MMP2*, *COL1A2*, *PCOLCE*, and *DCN* with longer RFS (**S3A Fig**). The extracellular matrix, including collagen, fibronectin, decorin, and related regulatory enzymes (*e*.*g*. MMPs), is the most abundant component in tumor microenvironment that can regulate tumor cell progression [72–74]. While higher MMP2 expression was associated with prolonged OS and RFS, post-treatment expression of another metalloproteinase ADAM15 was significantly low in both pathologic and radiologic non-responders (**Figs 5C and S4B**), implying a beneficial role of these metalloproteinases in response to ipilimumab and HDI therapy.

Further, genes related to epigenetic regulation were also differentially expressed, including an association of *EZH2* downregulation with radiologic response in this cohort (**Fig 2A and 2C**). Another study by Zingg *et al*. has demonstrated higher expression of EZH2 is linked to poor patient survival in melanoma [46]. Our findings support the emerging data that implicates the role of EZH2 histone methyltransferase in progression of melanoma and suppression of immune responses via various mechanisms including stabilization of the activated T_reg_ cells [75–77].

When looking further to determine gene expression changes in response to neoadjuvant ipilimumab and HDI treatment, we observed multiple immune related genes that achieved maximal differential expression before and after treatment. Among those genes, upregulated expression of *MARCO*, *RGCC*, *CCL14*, *CXCL2*, *PIK3R1*, and *ETS2* was associated with both pathologic and radiologic response (**Figs 5B and S4A**). These data suggest that regulation of NF-κB signaling or cell cycle as well as chemokine-mediated or transcription regulator-mediated immunoregulatory and inflammatory processes are associated with clinical response in post-therapy samples. Interestingly, there was increased expression of *JAK1* (Janus Kinase 1) after treatment in patients with pCR (**Fig 5B**). JAK1 plays a key role in IFN-α/β and IFN-γ signal transduction by phosphorylation of STAT signal transducers and transcription activators, and loss-of-function mutations in JAK1 or JAK2 have been identified as a mediator of primary resistance to pembrolizumab [78, 79]. Therefore, we can hypothesize that upregulation of *JAK1* by ipilimumab/HDI combination therapy may improve clinical response by potentiating the immunological environment to respond to subsequent anti-PD-1 treatment.

In our cohort, analysis of gene signatures indicated the significant correlation of multiple signatures to OS in baseline biopsy samples (**Fig 3C**). Most of these signatures are related to activation and function of lymphocytes (T cells, B cells, NK cells) as well as antigen presentation. MAGEs, CD8 T-cells, and PD-1 were associated with longer RFS (**S3B Fig**) and OS (**Fig 3C**). The signatures that were associated with response to ipilimumab and HDI combination therapy were TIGIT, CD45, cytotoxic cells, and T cells (**Fig 5D**), suggesting that efficient T cell infiltration correlates with response.

Next, we applied TIS algorithm to characterize the inflammatory profile of tumor samples and correlate it to OS. It was observed that TIS scores were associated with OS (*p* = 0.0166) (**Fig 3D**). In general, patients experienced a significant increase in TIS score after neoadjuvant ipilimumab and HDI therapy (**Fig 4B**), raising the possibility that this regimen may be effective in priming and enhancing the immunogenicity of less inflamed tumors. However, high TIS score post-therapy was not significantly associated with either pathological or radiologic response (**Figs 5D and S4C**), possibly attributable to the lower statistical power with the small sample size of subgroup analysis. This finding raises the possibility that ipilimumab and HDI combination therapy may be priming a naïve immune response or augmenting a pre-existing suboptimal immune response, but at the same time, inducing expression of compensatory immune checkpoints that restrict complete elimination of the tumor. In a study of single-dose pembrolizumab in resectable melanoma, response signature (*e*.*g*. T cell activation, adaptive immune response, and T cell migration) has been associated with clinical benefit while disease recurrence displayed a resistance phenotype, such as immune suppression, mutational escape, or tumor evolution [80]. Thus, sequential immunotherapy may be an appropriate strategy to achieve maximum clinical benefit in this patient population. Careful profiling of the immune response prior to and following each course of immune therapy will aid in the characterization of the evolving immune response and resistance.

Limitations of this study include the low number of patients enrolled. Our sample size might have not been adequate to demonstrate a statistically significant difference in critical analyses if correction for multiple testing was applied. Thus, the results of this retrospective analysis should be interpreted as hypothesis generating. Larger sample sizes and prospective validation are needed to confirm findings of this study and to ascertain whether gene expression signatures in patients can serve as prognostic and predictive biomarkers in relation to ipilimumab and HDI combination treatment as well as other immunotherapy agents. An additional limitation is the lack of validation of the immune signatures through immunohistochemistry due to limited tumor tissue availability.

## Conclusion

This is the first study to investigate the effects of neoadjuvant CTLA-4 blockade and IFN-α2b therapy in combination upon gene expression profiles in tumors of patients with locally/regionally advanced melanoma. Our work highlights the utility of gene expression profiling as a potential predictor of survival benefit and clinical response to immunotherapy. Additionally, we showed that comprehensive analysis of immune-related gene expression can serve as an important tool to unravel potential resistance mechanisms of immunotherapeutic combinations.

## Supporting information

S1 Fig Unsupervised hierarchical clustering (complete-linkage method) of gene expression signature in pre-treatment (baseline) vs. post-treatment samples. (TIF)

S2 Fig Comparison of baseline metastasis and primary tumor samples. **A**) Volcano plot of unadjusted *p*-value vs. log2-fold change of the differential expression between baseline metastasis and primary tumor samples (unadjusted *p* < 0.01). There were 13 samples for this analysis. **B**) Forest plot of gene signature scores between low expression and high expression in primary tumor samples. The position of the squared dots denotes the difference of score, and the size denotes the statistical significance. The horizontal lines are the Wald-type confidence intervals. There was no significant *p*-value in this analysis.
(TIF)

S3 Fig Genes and signatures associated with relapse-free survival (RFS) of patients treated with HDI and ipilimumab as a neoadjuvant therapy. **A**) Volcano plot of unadjusted *p*-value vs. log-hazard ratio of the differential expression associated with RFS of treated patients (unadjusted *p* < 0.01). Among 27 evaluable patients, 26 samples were available for this analysis. **B**) Forest plot of difference of gene signature scores based on hazard ratio between longer RFS and shorter RFS. The position of the squared dots denotes the difference of score, and the size denotes the statistical significance. The horizontal lines are the Wald-type confidence intervals. The * sign denotes the significance of *p*-value (< 0.05*). **C**) The Kaplan-Meier curves of TIS score groups for available samples (n = 26). Patients are stratified into “high” and “low” groups based on the TIS score 50% quantile. The Kaplan-Meier curves show that the high TIS score group demonstrated a trend of higher survival rate than the low TIS score group but statistically significant (*p*-value = 0.0917). The survival time was fit to TIS score group (high vs. low) with Cox proportional hazard model.
(TIF)

S4 Fig Post-treatment gene expression changes that correlate with radiologic response. **A**) Volcano plot of unadjusted *p*-value vs. log2-fold change of the differential expression after treatment, which associate with radiologic response (unadjusted p < 0.01). This analysis only includes data from 8 patients (n = 8) who exhibited radiologic response and for whom both baseline and post-treatment samples were available **B**) Was examine as in S4A Fig, but only includes data from 16 patients (n = 16) who did not exhibit radiologic response and for whom both baseline and post-treatment samples were available. **C and D**) Forest plot of difference of gene signature scores post-treatment in radiologic responders (**C**) and non-responders (**D**). The position of the squared dots denotes the difference of score, and the size denotes the statistical significance. The horizontal lines are the Wald-type confidence intervals. The * sign denotes the significance of *p*-value (< 0.01**, < 0.05*).
(TIF)

S5 Fig Schematic of experimental strategy and summary of results in this manuscript. (TIF)

S1 Table Genes analyzed in PanCancer immune profiling, PanCancer pathways, PanCancer progression, and custom panels. (PDF)

S2 Table Patient demographics and baseline disease characteristics of patients enrolled in clinical trial (N = 30 patients). (PDF)

## List of abbreviations

AAMP: angio-associated migratory cell protein
CD2AP: CD2 associated protein
CDKs: cyclin-dependent kinases
CHIT1: chitotriosidase 1
CR: complete response
EGFRs: epidermal growth factor receptors
FDA: US Food and Drug Administration
FFPE: formalin-fixed paraffin-embedded
HDI: interferon α-2b
IRAK1: IL-1 receptor-associated kinase 1
IL2RG: IL-2 receptor subunit gamma
JAk1: Janus Kinase 1
MAGEs: melanoma antigen genes
MARCO: macrophage pattern recognition scavenger receptor
MMPs: matrix metalloproteinases
mWHO: modified World Health Organization
NFKBIA: NF-κB inhibitor α
NK: natural killer
OS: overall survival
pCR: pathologic complete response
PD: progressive disease
PR: partial response
PRKAR2A: protein kinase cAMP-dependent type II regulatory subunit alpha
RAC1: Rac Family Small GTPase 1
RFS: relapse-free survival
SD: stable disease
SETD2: SET domain containing 2
SLAMF7: signaling lymphocytic activation molecule [SLAM] family member 7
TANK: TRAF family member associated NF-κB activator
TGF-β3: transforming growth factor-β3
TIGIT: T cell immunoreceptor with Ig and ITIM domains
TIS: tumor inflammation signature
TME: tumor microenvironment
TNFAIP3: TNF-α induced protein 3
