# Supplementary figures and images for: CTLA-4 blockade and interferon-α induce proinflammatory transcriptional changes in the tumor immune landscape that correlate with pathologic response in melanoma
Source: PLoS One. 2021 Jan 11;16(1):e0245287. doi: 10.1371/journal.pone.0245287 (PMC7799833; doi:10.1371/journal.pone.0245287)

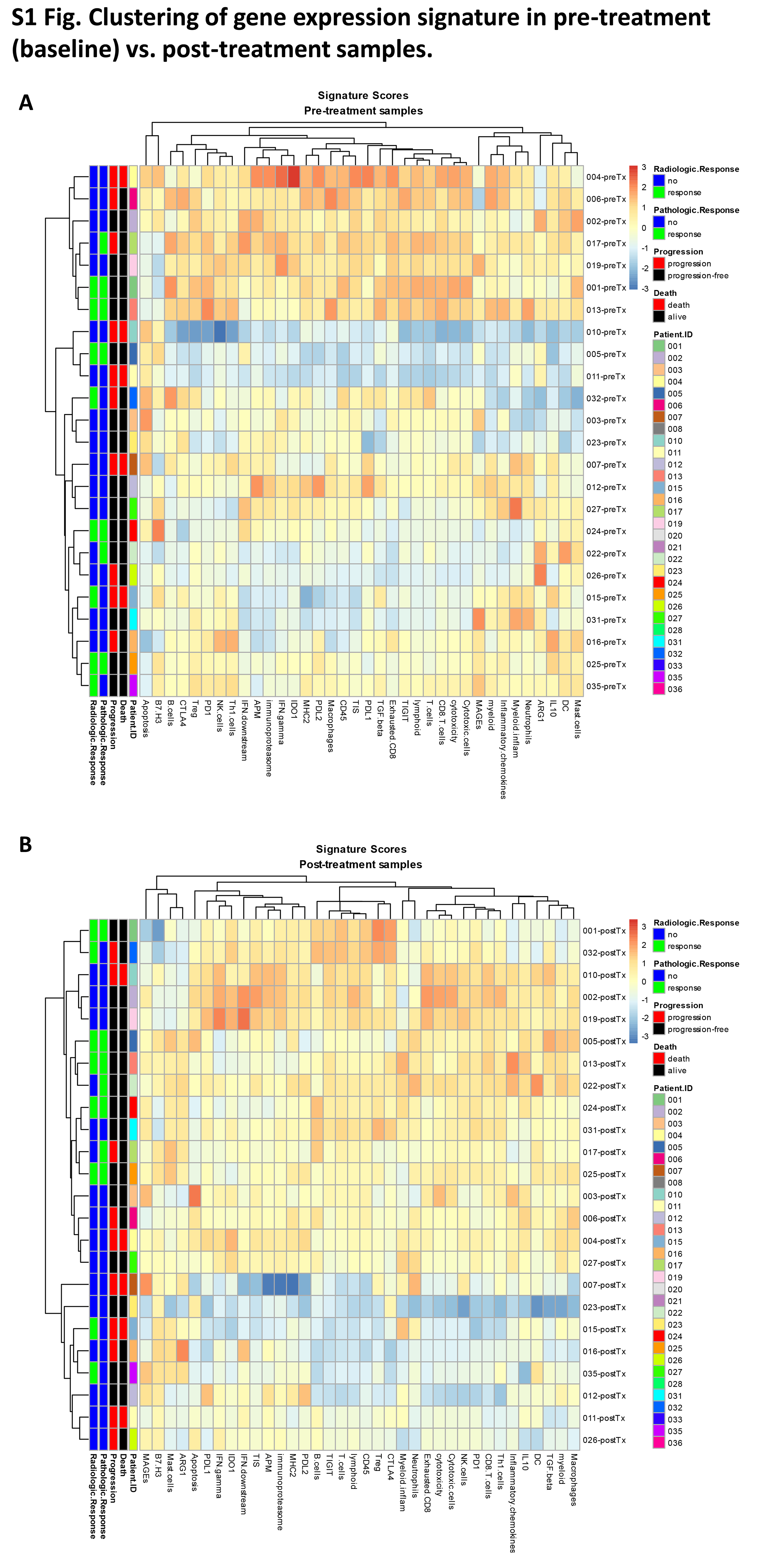

Supplement: S1 Fig — (TIF) [file pone.0245287.s001.tif]

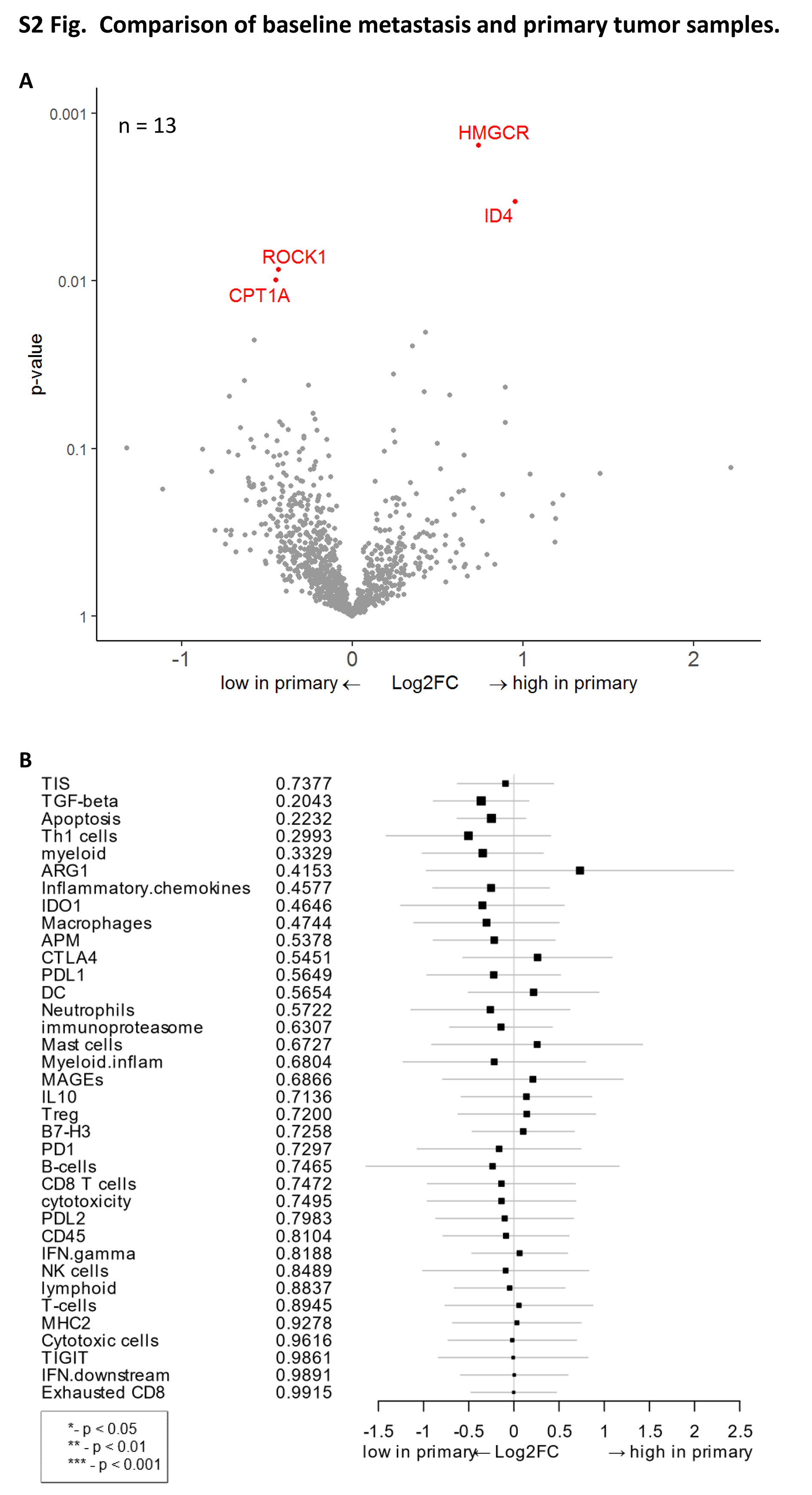

Supplement: S2 Fig — A) Volcano plot of unadjusted p-value vs. log2-fold change of the differential expression between baseline metastasis and primary tumor samples (unadjusted p < 0.01). There were 13 samples for this analysis. B) Forest plot of gene signature scores between low expression and high expression in primary tumor samples. The position of the squared dots denotes the difference of score, and the size denotes the statistical significance. The horizontal lines are the Wald-type confidence intervals. There was no significant p-value in this analysis. (TIF) [file pone.0245287.s002.tif]

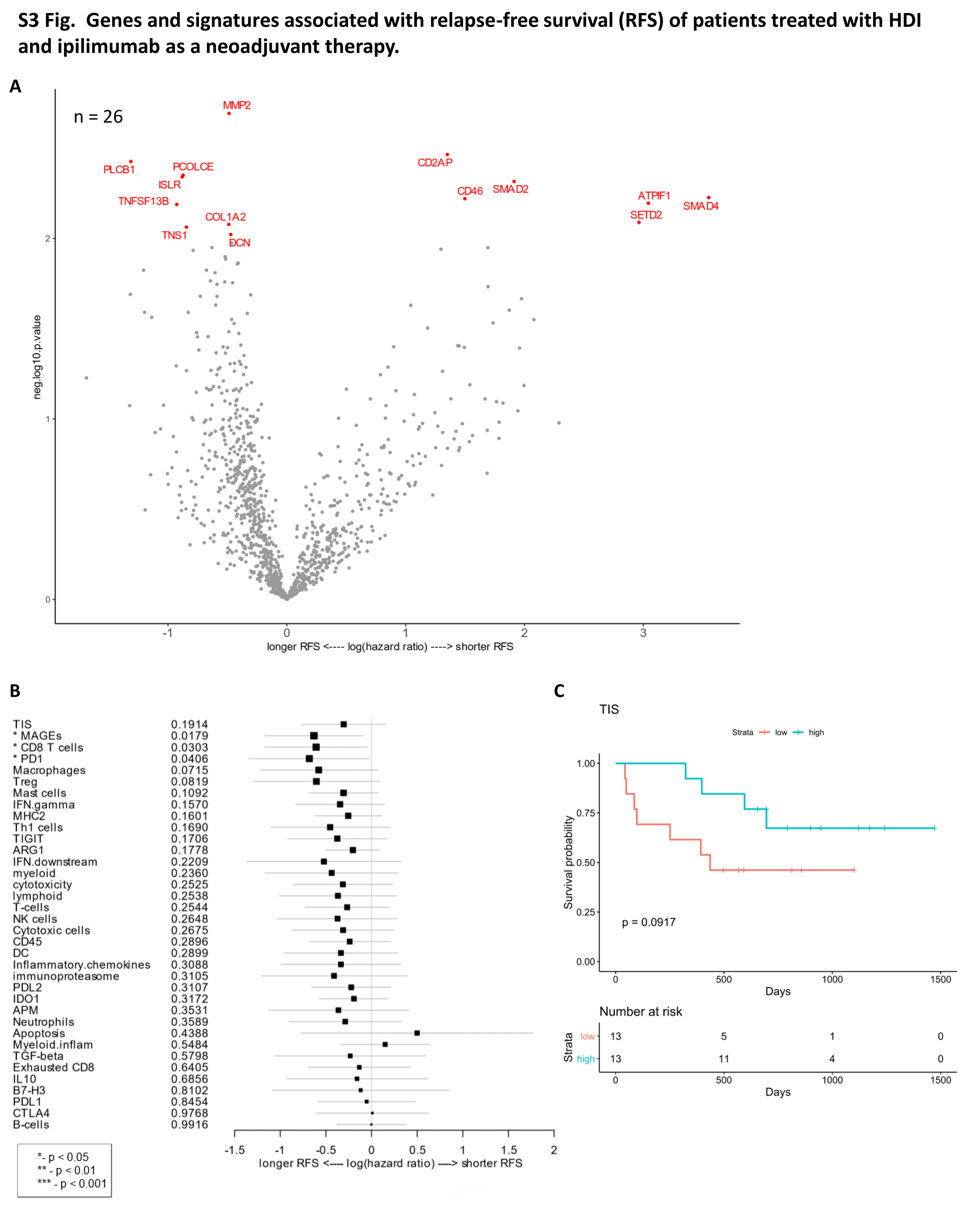

Supplement: S3 Fig — A) Volcano plot of unadjusted p-value vs. log-hazard ratio of the differential expression associated with RFS of treated patients (unadjusted p < 0.01). Among 27 evaluable patients, 26 samples were available for this analysis. B) Forest plot of difference of gene signature scores based on hazard ratio between longer RFS and shorter RFS. The position of the squared dots denotes the difference of score, and the size denotes the statistical significance. The horizontal lines are the Wald-type confidence intervals. The * sign denotes the significance of p-value (< 0.05*). C) The Kaplan-Meier curves of TIS score groups for available samples (n = 26). Patients are stratified into “high” and “low” groups based on the TIS score 50% quantile. The Kaplan-Meier curves show that the high TIS score group demonstrated a trend of higher survival rate than the low TIS score group but statistically significant (p-value = 0.0917). The survival time was fit to TIS score group (high vs. low) with Cox proportional hazard model. (TIF) [file pone.0245287.s003.tif]

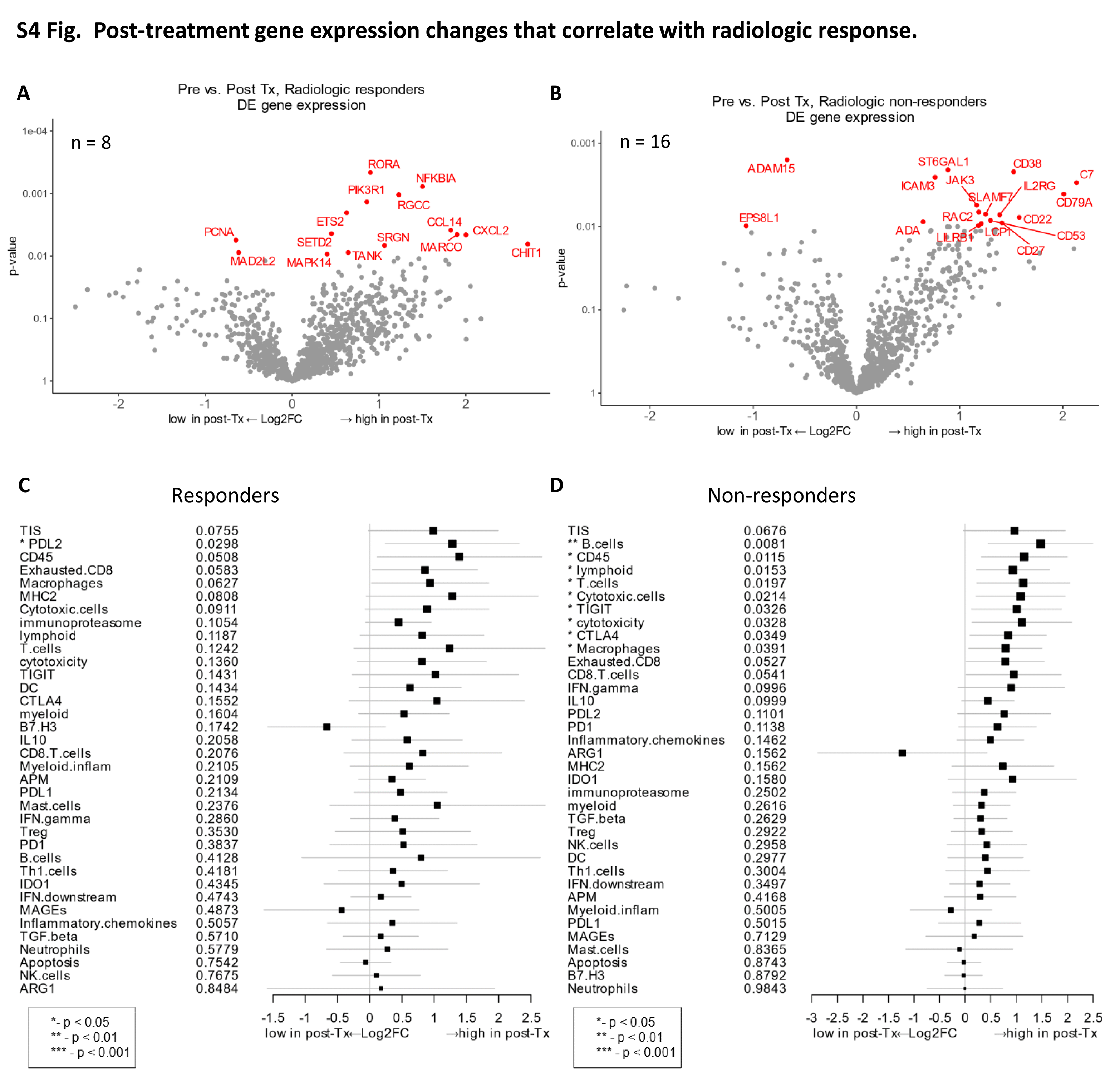

Supplement: S4 Fig — A) Volcano plot of unadjusted p-value vs. log2-fold change of the differential expression after treatment, which associate with radiologic response (unadjusted p < 0.01). This analysis only includes data from 8 patients (n = 8) who exhibited radiologic response and for whom both baseline and post-treatment samples were available B) Was examine as in S4A Fig, but only includes data from 16 patients (n = 16) who did not exhibit radiologic response and for whom both baseline and post-treatment samples were available. C and D) Forest plot of difference of gene signature scores post-treatment in radiologic responders (C) and non-responders (D). The position of the squared dots denotes the difference of score, and the size denotes the statistical significance. The horizontal lines are the Wald-type confidence intervals. The * sign denotes the significance of p-value (< 0.01**, < 0.05*). (TIF) [file pone.0245287.s004.tif]

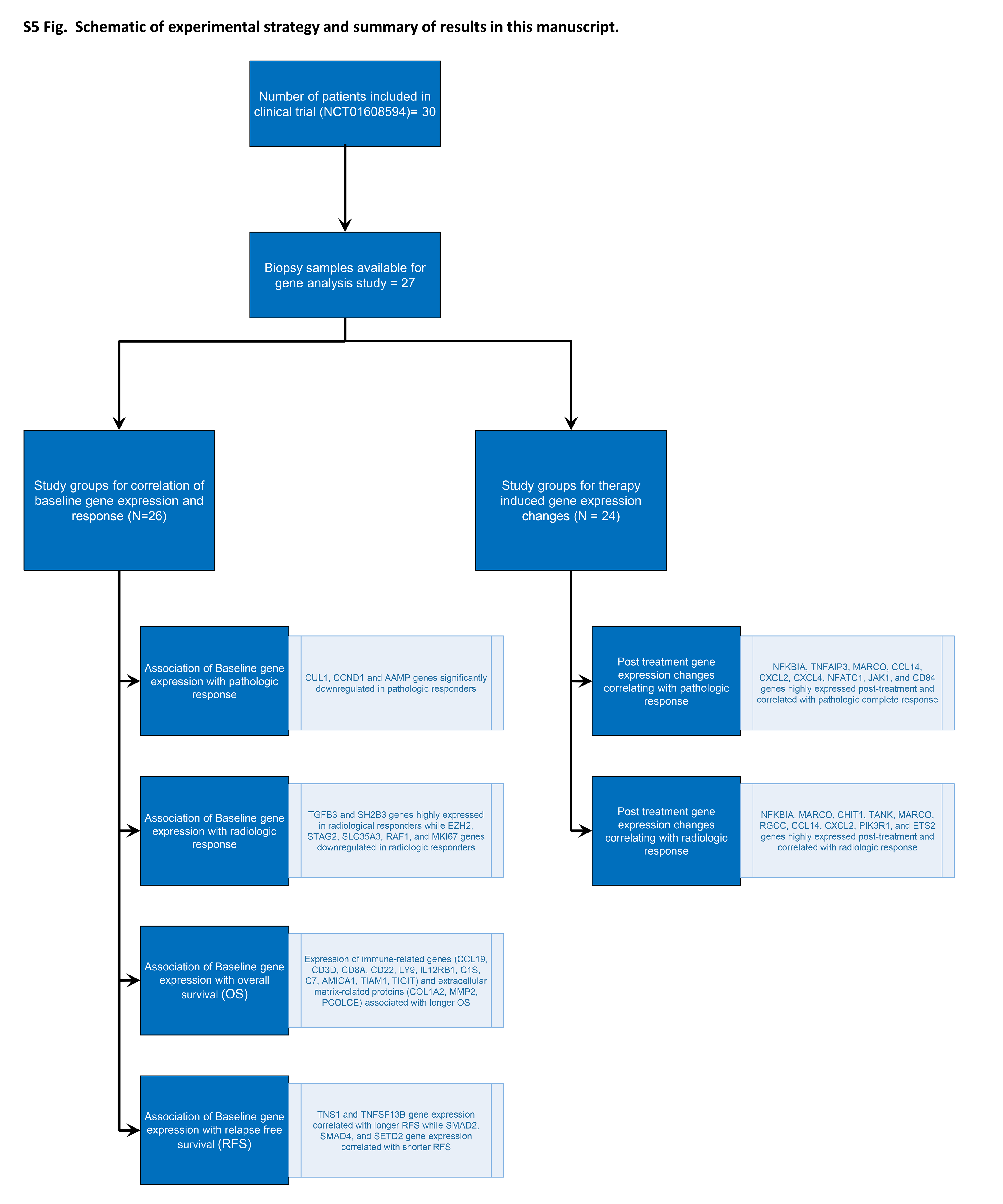

Supplement: S5 Fig — (TIF) [file pone.0245287.s005.tif]
