# Supplementary material for: CTLA-4 blockade and interferon-α induce proinflammatory transcriptional changes in the tumor immune landscape that correlate with pathologic response in melanoma
Source: PLoS One. 2021 Jan 11;16(1):e0245287. doi: 10.1371/journal.pone.0245287 (PMC7799833; doi:10.1371/journal.pone.0245287)
Supplement: S1 Table — (PDF) [file pone.0245287.s006.pdf]

Supplementary table of genes:

Gene Symbol	Gene Name	Panel
A2M	alpha-2-macroglobulin	PanCancer Immune Profiling
ABCB1	ATP-binding cassette, sub-family B (MDR/TAP), member 1	PanCancer Immune Profiling
ABL1	c-abl oncogene 1, non-receptor tyrosine kinase	PanCancer Immune Profiling
ADA	adenosine deaminase	PanCancer Immune Profiling
ADORA2A	adenosine A2a receptor	PanCancer Immune Profiling
AICDA	activation-induced cytidine deaminase	PanCancer Immune Profiling
AIRE	autoimmune regulator	PanCancer Immune Profiling
AKT3	v-akt murine thymoma viral oncogene homolog 3 (protein kinase B, gamma)	PanCancer Immune Profiling
ALCAM	activated leukocyte cell adhesion molecule	PanCancer Immune Profiling
AMBP	alpha-1-microglobulin/bikunin precursor	PanCancer Immune Profiling
AMICA1	adhesion molecule, interacts with CXADR antigen 1	PanCancer Immune Profiling
ANP32B	acidic (leucine-rich) nuclear phosphoprotein 32 family, member B	PanCancer Immune Profiling
ANXA1	annexin A1	PanCancer Immune Profiling
APOE	apolipoprotein E	PanCancer Immune Profiling

APP	amyloid beta (A4) precursor protein	PanCancer Immune Profiling
ARG1	arginase, liver	PanCancer Immune Profiling
ARG2	arginase, type II	PanCancer Immune Profiling
ATF1	activating transcription factor 1	PanCancer Immune Profiling
ATF2	activating transcription factor 2	PanCancer Immune Profiling
ATG10	autophagy related 10	PanCancer Immune Profiling
ATG12	autophagy related 12	PanCancer Immune Profiling
ATG16L1	autophagy related 16-like 1 (S. cerevisiae)	PanCancer Immune Profiling
ATG5	autophagy related 5	PanCancer Immune Profiling
ATG7	autophagy related 7	PanCancer Immune Profiling
ATM	ataxia telangiectasia mutated	PanCancer Immune Profiling
AXL	AXL receptor tyrosine kinase	PanCancer Immune Profiling
BAGE	B melanoma antigen	PanCancer Immune Profiling
BATF	basic leucine zipper transcription factor, ATF-like	PanCancer Immune Profiling
BAX	BCL2-associated X protein	PanCancer Immune Profiling

BCL10	B-cell CLL/lymphoma 10	PanCancer Immune Profiling
BCL2	B-cell CLL/lymphoma 2	PanCancer Immune Profiling
BCL2L1	BCL2-like 1	PanCancer Immune Profiling
BCL6	B-cell CLL/lymphoma 6	PanCancer Immune Profiling
BID	BH3 interacting domain death agonist	PanCancer Immune Profiling
BIRC5	baculoviral IAP repeat containing 5	PanCancer Immune Profiling
BLK	B lymphoid tyrosine kinase	PanCancer Immune Profiling
BLNK	B-cell linker	PanCancer Immune Profiling
BMI1	BMI1 polycomb ring finger oncogene	PanCancer Immune Profiling
BST1	bone marrow stromal cell antigen 1	PanCancer Immune Profiling
BST2	bone marrow stromal cell antigen 2	PanCancer Immune Profiling
BTK	Bruton agammaglobulinemia tyrosine kinase	PanCancer Immune Profiling
BTLA	B and T lymphocyte associated	PanCancer Immune Profiling
C1QA	complement component 1, q subcomponent, A chain	PanCancer Immune Profiling
C1QB	complement component 1, q subcomponent, B chain	PanCancer Immune Profiling

C1QBP	complement component 1, q subcomponent binding protein	PanCancer Immune Profiling
C1R	complement component 1, r subcomponent	PanCancer Immune Profiling
C1S	complement component 1, s subcomponent	PanCancer Immune Profiling
C2	complement component 2	PanCancer Immune Profiling
C3	complement component 3	PanCancer Immune Profiling
C3AR1	complement component 3a receptor 1	PanCancer Immune Profiling
C4B	complement component 4B (Chido blood group)	PanCancer Immune Profiling
C4BPA	complement component 4 binding protein, alpha	PanCancer Immune Profiling
C5	complement component 5	PanCancer Immune Profiling
C6	complement component 6	PanCancer Immune Profiling
C7	complement component 7	PanCancer Immune Profiling
C8A	complement component 8, alpha polypeptide	PanCancer Immune Profiling
C8B	complement component 8, beta polypeptide	PanCancer Immune Profiling
C8G	complement component 8, gamma polypeptide	PanCancer Immune Profiling
C9	complement component 9	PanCancer Immune Profiling

CAMP	cathelicidin antimicrobial peptide	PanCancer Immune Profiling
CARD11	caspase recruitment domain family, member 11	PanCancer Immune Profiling
CARD9	caspase recruitment domain family, member 9	PanCancer Immune Profiling
CASP1	caspase 1, apoptosis-related cysteine peptidase	PanCancer Immune Profiling
CASP10	caspase 10, apoptosis-related cysteine peptidase	PanCancer Immune Profiling
CASP3	caspase 3, apoptosis-related cysteine peptidase	PanCancer Immune Profiling
CASP8	caspase 8, apoptosis-related cysteine peptidase	PanCancer Immune Profiling
CCL1	chemokine (C-C motif) ligand 1	PanCancer Immune Profiling
CCL11	chemokine (C-C motif) ligand 11	PanCancer Immune Profiling
CCL13	chemokine (C-C motif) ligand 13	PanCancer Immune Profiling
CCL14	chemokine (C-C motif) ligand 14	PanCancer Immune Profiling
CCL15	chemokine (C-C motif) ligand 15	PanCancer Immune Profiling
CCL16	chemokine (C-C motif) ligand 16	PanCancer Immune Profiling
CCL17	chemokine (C-C motif) ligand 17	PanCancer Immune Profiling
CCL18	chemokine (C-C motif) ligand 18 (pulmonary and activation-regulated)	PanCancer Immune Profiling

CCL19	chemokine (C-C motif) ligand 19	PanCancer Immune Profiling
CCL2	chemokine (C-C motif) ligand 2	PanCancer Immune Profiling
CCL20	chemokine (C-C motif) ligand 20	PanCancer Immune Profiling
CCL21	chemokine (C-C motif) ligand 21	PanCancer Immune Profiling
CCL22	chemokine (C-C motif) ligand 22	PanCancer Immune Profiling
CCL23	chemokine (C-C motif) ligand 23	PanCancer Immune Profiling
CCL24	chemokine (C-C motif) ligand 24	PanCancer Immune Profiling
CCL25	chemokine (C-C motif) ligand 25	PanCancer Immune Profiling
CCL26	chemokine (C-C motif) ligand 26	PanCancer Immune Profiling
CCL27	chemokine (C-C motif) ligand 27	PanCancer Immune Profiling
CCL28	chemokine (C-C motif) ligand 28	PanCancer Immune Profiling
CCL3	chemokine (C-C motif) ligand 3	PanCancer Immune Profiling
CCL3L1	chemokine (C-C motif) ligand 3-like 1	PanCancer Immune Profiling
CCL4	chemokine (C-C motif) ligand 4	PanCancer Immune Profiling
CCL5	chemokine (C-C motif) ligand 5	PanCancer Immune Profiling

CCL7	chemokine (C-C motif) ligand 7	PanCancer Immune Profiling
CCL8	chemokine (C-C motif) ligand 8	PanCancer Immune Profiling
CCND3	cyclin D3	PanCancer Immune Profiling
CCR1	chemokine (C-C motif) receptor 1	PanCancer Immune Profiling
CCR2	chemokine (C-C motif) receptor 2	PanCancer Immune Profiling
CCR3	chemokine (C-C motif) receptor 3	PanCancer Immune Profiling
CCR4	chemokine (C-C motif) receptor 4	PanCancer Immune Profiling
CCR5	chemokine (C-C motif) receptor 5 (gene/pseudogene)	PanCancer Immune Profiling
CCR6	chemokine (C-C motif) receptor 6	PanCancer Immune Profiling
CCR7	chemokine (C-C motif) receptor 7	PanCancer Immune Profiling
CCR9	chemokine (C-C motif) receptor 9	PanCancer Immune Profiling
CCRL2	chemokine (C-C motif) receptor-like 2	PanCancer Immune Profiling
CD14	CD14 molecule	PanCancer Immune Profiling
CD160	CD160 molecule	PanCancer Immune Profiling
CD163	CD163 molecule	PanCancer Immune Profiling

CD164	CD164 molecule, sialomucin	PanCancer Immune Profiling
CD180	CD180 molecule	PanCancer Immune Profiling
CD19	CD19 molecule	PanCancer Immune Profiling
CD1A	CD1a molecule	PanCancer Immune Profiling
CD1B	CD1b molecule	PanCancer Immune Profiling
CD1C	CD1c molecule	PanCancer Immune Profiling
CD1D	CD1d molecule	PanCancer Immune Profiling
CD1E	CD1e molecule	PanCancer Immune Profiling
CD2	CD2 molecule	PanCancer Immune Profiling
CD200	CD200 molecule	PanCancer Immune Profiling
CD207	CD207 molecule, langerin	PanCancer Immune Profiling
CD209	CD209 molecule	PanCancer Immune Profiling
CD22	CD22 molecule	PanCancer Immune Profiling
CD24	CD24 molecule	PanCancer Immune Profiling
CD244	CD244 molecule, natural killer cell receptor 2B4	PanCancer Immune Profiling

CD247	CD247 molecule	PanCancer Immune Profiling
CD27	CD27 molecule	PanCancer Immune Profiling
CD274	CD274 molecule	PanCancer Immune Profiling
CD276	CD276 molecule	PanCancer Immune Profiling
CD28	CD28 molecule	PanCancer Immune Profiling
CD33	CD33 molecule	PanCancer Immune Profiling
CD34	CD34 molecule	PanCancer Immune Profiling
CD36	CD36 molecule (thrombospondin receptor)	PanCancer Immune Profiling
CD37	CD37 molecule	PanCancer Immune Profiling
CD38	CD38 molecule	PanCancer Immune Profiling
CD3D	CD3d molecule, delta (CD3-TCR complex)	PanCancer Immune Profiling
CD3E	CD3e molecule, epsilon (CD3-TCR complex)	PanCancer Immune Profiling
CD3EAP	CD3e molecule, epsilon associated protein	PanCancer Immune Profiling
CD3G	CD3g molecule, gamma (CD3-TCR complex)	PanCancer Immune Profiling
CD4	CD4 molecule	PanCancer Immune Profiling

CD40	CD40 molecule, TNF receptor superfamily member 5	PanCancer Immune Profiling
CD40LG	CD40 ligand	PanCancer Immune Profiling
CD44	CD44 molecule (Indian blood group)	PanCancer Immune Profiling
CD46	CD46 molecule, complement regulatory protein	PanCancer Immune Profiling
CD47	CD47 molecule	PanCancer Immune Profiling
CD48	CD48 molecule	PanCancer Immune Profiling
CD5	CD5 molecule	PanCancer Immune Profiling
CD53	CD53 molecule	PanCancer Immune Profiling
CD55	CD55 molecule, decay accelerating factor for complement (Cromer blood group)	PanCancer Immune Profiling
CD58	CD58 molecule	PanCancer Immune Profiling
CD59	CD59 molecule, complement regulatory protein	PanCancer Immune Profiling
CD6	CD6 molecule	PanCancer Immune Profiling
CD63	CD63 molecule	PanCancer Immune Profiling
CD68	CD68 molecule	PanCancer Immune Profiling
CD7	CD7 molecule	PanCancer Immune Profiling

CD70	CD70 molecule	PanCancer Immune Profiling
CD74	CD74 molecule, major histocompatibility complex, class II invariant chain	PanCancer Immune Profiling
CD79A	CD79a molecule, immunoglobulin-associated alpha	PanCancer Immune Profiling
CD79B	CD79b molecule, immunoglobulin-associated beta	PanCancer Immune Profiling
CD80	CD80 molecule	PanCancer Immune Profiling
CD81	CD81 molecule	PanCancer Immune Profiling
CD83	CD83 molecule	PanCancer Immune Profiling
CD84	CD84 molecule	PanCancer Immune Profiling
CD86	CD86 molecule	PanCancer Immune Profiling
CD8A	CD8a molecule	PanCancer Immune Profiling
CD8B	CD8b molecule	PanCancer Immune Profiling
CD9	CD9 molecule	PanCancer Immune Profiling
CD96	CD96 molecule	PanCancer Immune Profiling
CD97	CD97 molecule	PanCancer Immune Profiling
CD99	CD99 molecule	PanCancer Immune Profiling

CDH1	cadherin 1, type 1, E-cadherin (epithelial)	PanCancer Immune Profiling
CDH5	cadherin 5, type 2 (vascular endothelium)	PanCancer Immune Profiling
CDK1	cyclin-dependent kinase 1	PanCancer Immune Profiling
CDKN1A	cyclin-dependent kinase inhibitor 1A (p21, Cip1)	PanCancer Immune Profiling
CEACAM1	carcinoembryonic antigen-related cell adhesion molecule 1 (biliary glycoprotein)	PanCancer Immune Profiling
CEACAM6	carcinoembryonic antigen-related cell adhesion molecule 6 (non-specific cross reacting antigen)	PanCancer Immune Profiling
CEACAM8	carcinoembryonic antigen-related cell adhesion molecule 8	PanCancer Immune Profiling
CEBPB	CCAAT/enhancer binding protein (C/EBP), beta	PanCancer Immune Profiling
CFB	complement factor B	PanCancer Immune Profiling
CFD	complement factor D (adipsin)	PanCancer Immune Profiling
CFI	complement factor I	PanCancer Immune Profiling
CFP	complement factor properdin	PanCancer Immune Profiling
CHIT1	chitinase 1 (chitotriosidase)	PanCancer Immune Profiling
CHUK	conserved helix-loop-helix ubiquitous kinase	PanCancer Immune Profiling
CKLF	chemokine-like factor	PanCancer Immune Profiling

CLEC4A	C-type lectin domain family 4, member A	PanCancer Immune Profiling
CLEC4C	C-type lectin domain family 4, member C	PanCancer Immune Profiling
CLEC5A	C-type lectin domain family 5, member A	PanCancer Immune Profiling
CLEC6A	C-type lectin domain family 6, member A	PanCancer Immune Profiling
CLEC7A	C-type lectin domain family 7, member A	PanCancer Immune Profiling
CLU	clusterin	PanCancer Immune Profiling
CMA1	chymase 1, mast cell	PanCancer Immune Profiling
CMKLR1	chemokine-like receptor 1	PanCancer Immune Profiling
COL3A1	collagen, type III, alpha 1	PanCancer Immune Profiling
COLEC12	collectin sub-family member 12	PanCancer Immune Profiling
CR1	complement component (3b/4b) receptor 1 (Knops blood group)	PanCancer Immune Profiling
CR2	complement component (3d/Epstein Barr virus) receptor 2	PanCancer Immune Profiling
CREB1	cAMP responsive element binding protein 1	PanCancer Immune Profiling
CREB5	cAMP responsive element binding protein 5	PanCancer Immune Profiling
CREBBP	CREB binding protein	PanCancer Immune Profiling

CRP	C-reactive protein, pentraxin-related	PanCancer Immune Profiling
CSF1	colony stimulating factor 1 (macrophage)	PanCancer Immune Profiling
CSF1R	colony stimulating factor 1 receptor	PanCancer Immune Profiling
CSF2	colony stimulating factor 2 (granulocyte-macrophage)	PanCancer Immune Profiling
CSF2RB	colony stimulating factor 2 receptor, beta, low-affinity (granulocyte-macrophage)	PanCancer Immune Profiling
CSF3	colony stimulating factor 3 (granulocyte)	PanCancer Immune Profiling
CSF3R	colony stimulating factor 3 receptor (granulocyte)	PanCancer Immune Profiling
CT45A1	cancer/testis antigen family 45, member A1	PanCancer Immune Profiling
CTAG1B	cancer/testis antigen 1B	PanCancer Immune Profiling
CTAGE1	cutaneous T-cell lymphoma-associated antigen 1	PanCancer Immune Profiling
CTCFL	CCCTC-binding factor (zinc finger protein)-like	PanCancer Immune Profiling
CTLA4	cytotoxic T-lymphocyte-associated protein 4	PanCancer Immune Profiling
CTSG	cathepsin G	PanCancer Immune Profiling
CTSH	cathepsin H	PanCancer Immune Profiling
CTSL	cathepsin L	PanCancer Immune Profiling

CTSS	cathepsin S	PanCancer Immune Profiling
CTSW	cathepsin W	PanCancer Immune Profiling
CX3CL1	chemokine (C-X3-C motif) ligand 1	PanCancer Immune Profiling
CX3CR1	chemokine (C-X3-C motif) receptor 1	PanCancer Immune Profiling
CXCL1	chemokine (C-X-C motif) ligand 1 (melanoma growth stimulating activity, alpha)	PanCancer Immune Profiling
CXCL10	chemokine (C-X-C motif) ligand 10	PanCancer Immune Profiling
CXCL11	chemokine (C-X-C motif) ligand 11	PanCancer Immune Profiling
CXCL12	chemokine (C-X-C motif) ligand 12	PanCancer Immune Profiling
CXCL13	chemokine (C-X-C motif) ligand 13	PanCancer Immune Profiling
CXCL14	chemokine (C-X-C motif) ligand 14	PanCancer Immune Profiling
CXCL16	chemokine (C-X-C motif) ligand 16	PanCancer Immune Profiling
CXCL2	chemokine (C-X-C motif) ligand 2	PanCancer Immune Profiling
CXCL3	chemokine (C-X-C motif) ligand 3	PanCancer Immune Profiling
CXCL5	chemokine (C-X-C motif) ligand 5	PanCancer Immune Profiling
CXCL6	chemokine (C-X-C motif) ligand 6 (granulocyte chemotactic protein 2)	PanCancer Immune Profiling

CXCL8	chemokine (C-X-C motif) ligand 8	PanCancer Immune Profiling
CXCL9	chemokine (C-X-C motif) ligand 9	PanCancer Immune Profiling
CXCR1	chemokine (C-X-C motif) receptor 1	PanCancer Immune Profiling
CXCR2	chemokine (C-X-C motif) receptor 2	PanCancer Immune Profiling
CXCR3	chemokine (C-X-C motif) receptor 3	PanCancer Immune Profiling
CXCR4	chemokine (C-X-C motif) receptor 4	PanCancer Immune Profiling
CXCR5	chemokine (C-X-C motif) receptor 5	PanCancer Immune Profiling
CXCR6	chemokine (C-X-C motif) receptor 6	PanCancer Immune Profiling
CYBB	cytochrome b-245, beta polypeptide	PanCancer Immune Profiling
CYFIP2	cytoplasmic FMR1 interacting protein 2	PanCancer Immune Profiling
CYLD	cylindromatosis (turban tumor syndrome)	PanCancer Immune Profiling
DDX43	DEAD (Asp-Glu-Ala-Asp) box polypeptide 43	PanCancer Immune Profiling
DDX58	DEAD (Asp-Glu-Ala-Asp) box polypeptide 58	PanCancer Immune Profiling
DEFB1	defensin, beta 1	PanCancer Immune Profiling
DMBT1	deleted in malignant brain tumors 1	PanCancer Immune Profiling

DOCK9	dedicator of cytokinesis 9	PanCancer Immune Profiling
DPP4	dipeptidyl-peptidase 4	PanCancer Immune Profiling
DUSP4	dual specificity phosphatase 4	PanCancer Immune Profiling
DUSP6	dual specificity phosphatase 6	PanCancer Immune Profiling
EBI3	Epstein-Barr virus induced 3	PanCancer Immune Profiling
ECSIT	ECSIT homolog (Drosophila)	PanCancer Immune Profiling
EGR1	early growth response 1	PanCancer Immune Profiling
EGR2	early growth response 2	PanCancer Immune Profiling
ELANE	elastase, neutrophil expressed	PanCancer Immune Profiling
ELK1	ELK1, member of ETS oncogene family	PanCancer Immune Profiling
ENG	endoglin	PanCancer Immune Profiling
ENTPD1	ectonucleoside triphosphate diphosphohydrolase 1	PanCancer Immune Profiling
EOMES	eomesodermin	PanCancer Immune Profiling
EP300	E1A binding protein p300	PanCancer Immune Profiling
EPCAM	epithelial cell adhesion molecule	PanCancer Immune Profiling

ETS1	v-ets erythroblastosis virus E26 oncogene homolog 1 (avian)	PanCancer Immune Profiling
EWSR1	Ewing sarcoma breakpoint region 1	PanCancer Immune Profiling
F12	coagulation factor XII (Hageman factor)	PanCancer Immune Profiling
F13A1	coagulation factor XIII, A1 polypeptide	PanCancer Immune Profiling
F2RL1	coagulation factor II (thrombin) receptor-like 1	PanCancer Immune Profiling
FADD	Fas (TNFRSF6)-associated via death domain	PanCancer Immune Profiling
FAS	Fas (TNF receptor superfamily, member 6)	PanCancer Immune Profiling
FCER1A	Fc fragment of IgE, high affinity I, receptor for; alpha polypeptide	PanCancer Immune Profiling
FCER1G	Fc fragment of IgE, high affinity I, receptor for; gamma polypeptide	PanCancer Immune Profiling
FCER2	Fc fragment of IgE, low affinity II, receptor for (CD23)	PanCancer Immune Profiling
FCGR1A	Fc fragment of IgG, high affinity Ia, receptor (CD64)	PanCancer Immune Profiling
FCGR2A	Fc fragment of IgG, low affinity IIa, receptor (CD32)	PanCancer Immune Profiling
FCGR2B	Fc fragment of IgG, low affinity IIb, receptor (CD32)	PanCancer Immune Profiling
FCGR3A	Fc fragment of IgG, low affinity IIIa, receptor (CD16a)	PanCancer Immune Profiling
FEZ1	fasciculation and elongation protein zeta 1 (zygin I)	PanCancer Immune Profiling

FLT3	fms-related tyrosine kinase 3	PanCancer Immune Profiling
FLT3LG	fms-related tyrosine kinase 3 ligand	PanCancer Immune Profiling
FN1	fibronectin 1	PanCancer Immune Profiling
FOS	FBJ murine osteosarcoma viral oncogene homolog	PanCancer Immune Profiling
FOXJ1	forkhead box J1	PanCancer Immune Profiling
FOXP3	forkhead box P3	PanCancer Immune Profiling
FPR2	formyl peptide receptor 2	PanCancer Immune Profiling
FUT5	fucosyltransferase 5 (alpha (1,3) fucosyltransferase)	PanCancer Immune Profiling
FUT7	fucosyltransferase 7 (alpha (1,3) fucosyltransferase)	PanCancer Immune Profiling
FYN	FYN oncogene related to SRC, FGR, YES	PanCancer Immune Profiling
GAGE1	G antigen 1	PanCancer Immune Profiling
GATA3	GATA binding protein 3	PanCancer Immune Profiling
GNLY	granulysin	PanCancer Immune Profiling
GPI	glucose-6-phosphate isomerase	PanCancer Immune Profiling
GTF3C1	general transcription factor IIIC, polypeptide 1, alpha 220kDa	PanCancer Immune Profiling

GZMA	granzyme A (granzyme 1, cytotoxic T-lymphocyte-associated serine esterase 3)	PanCancer Immune Profiling
GZMB	granzyme B (granzyme 2, cytotoxic T-lymphocyte-associated serine esterase 1)	PanCancer Immune Profiling
GZMH	granzyme H (cathepsin G-like 2, protein h-CCPX)	PanCancer Immune Profiling
GZMK	granzyme K (granzyme 3; tryptase II)	PanCancer Immune Profiling
GZMM	granzyme M (lymphocyte met-ase 1)	PanCancer Immune Profiling
HAMP	hepcidin antimicrobial peptide	PanCancer Immune Profiling
HAVCR2	hepatitis A virus cellular receptor 2	PanCancer Immune Profiling
HCK	hemopoietic cell kinase	PanCancer Immune Profiling
HLA-A	major histocompatibility complex, class I, A	PanCancer Immune Profiling
HLA-B	major histocompatibility complex, class I, B	PanCancer Immune Profiling
HLA-C	major histocompatibility complex, class I, C	PanCancer Immune Profiling
HLA-DMA	major histocompatibility complex, class II, DM alpha	PanCancer Immune Profiling
HLA-DMB	major histocompatibility complex, class II, DM beta	PanCancer Immune Profiling
HLA-DOB	major histocompatibility complex, class II, DO beta	PanCancer Immune Profiling
HLA-DPA1	major histocompatibility complex, class II, DP alpha 1	PanCancer Immune Profiling

HLA-DPB1	major histocompatibility complex, class II, DP beta 1	PanCancer Immune Profiling
HLA-DQA1	major histocompatibility complex, class II, DQ alpha 1	PanCancer Immune Profiling
HLA-DQB1	major histocompatibility complex, class II, DQ beta 1	PanCancer Immune Profiling
HLA-DRA	major histocompatibility complex, class II, DR alpha	PanCancer Immune Profiling
HLA-DRB3	major histocompatibility complex, class II, DR beta 3	PanCancer Immune Profiling
HLA-DRB4	major histocompatibility complex, class II, DR beta 4	PanCancer Immune Profiling
HLA-E	major histocompatibility complex, class I, E	PanCancer Immune Profiling
HLA-G	major histocompatibility complex, class I, G	PanCancer Immune Profiling
HMGB1	high mobility group box 1	PanCancer Immune Profiling
HRAS	v-Ha-ras Harvey rat sarcoma viral oncogene homolog	PanCancer Immune Profiling
HSD11B1	hydroxysteroid (11-beta) dehydrogenase 1	PanCancer Immune Profiling
ICAM1	intercellular adhesion molecule 1	PanCancer Immune Profiling
ICAM2	intercellular adhesion molecule 2	PanCancer Immune Profiling
ICAM3	intercellular adhesion molecule 3	PanCancer Immune Profiling
ICAM4	intercellular adhesion molecule 4 (Landsteiner-Wiener blood group)	PanCancer Immune Profiling

ICOS	inducible T-cell co-stimulator	PanCancer Immune Profiling
ICOSLG	inducible T-cell co-stimulator ligand	PanCancer Immune Profiling
IDO1	indoleamine 2,3-dioxygenase 1	PanCancer Immune Profiling
IFI16	interferon, gamma-inducible protein 16	PanCancer Immune Profiling
IFI27	interferon, alpha-inducible protein 27	PanCancer Immune Profiling
IFI35	interferon-induced protein 35	PanCancer Immune Profiling
IFIH1	interferon induced with helicase C domain 1	PanCancer Immune Profiling
IFIT1	interferon-induced protein with tetratricopeptide repeats 1	PanCancer Immune Profiling
IFIT2	interferon-induced protein with tetratricopeptide repeats 2	PanCancer Immune Profiling
IFITM1	interferon induced transmembrane protein 1	PanCancer Immune Profiling
IFITM2	interferon induced transmembrane protein 2	PanCancer Immune Profiling
IFNA1	interferon, alpha 1	PanCancer Immune Profiling
IFNA17	interferon, alpha 17	PanCancer Immune Profiling
IFNA2	interferon, alpha 2	PanCancer Immune Profiling
IFNA7	interferon, alpha 7	PanCancer Immune Profiling

IFNA8	interferon, alpha 8	PanCancer Immune Profiling
IFNAR1	interferon (alpha, beta and omega) receptor 1	PanCancer Immune Profiling
IFNAR2	interferon (alpha, beta and omega) receptor 2	PanCancer Immune Profiling
IFNB1	interferon, beta 1, fibroblast	PanCancer Immune Profiling
IFNG	interferon, gamma	PanCancer Immune Profiling
IFNGR1	interferon gamma receptor 1	PanCancer Immune Profiling
IFNL1	interferon lambda 1	PanCancer Immune Profiling
IFNL2	interferon lambda 2	PanCancer Immune Profiling
IGF1R	insulin-like growth factor 1 receptor	PanCancer Immune Profiling
IGF2R	insulin-like growth factor 2 receptor	PanCancer Immune Profiling
IGLL1	immunoglobulin lambda-like polypeptide 1	PanCancer Immune Profiling
IKBKB	inhibitor of kappa light polypeptide gene enhancer in B-cells, kinase beta	PanCancer Immune Profiling
IKBKE	inhibitor of kappa light polypeptide gene enhancer in B-cells, kinase epsilon	PanCancer Immune Profiling
IKBKG	inhibitor of kappa light polypeptide gene enhancer in B-cells, kinase gamma	PanCancer Immune Profiling
IL10	interleukin 10	PanCancer Immune Profiling

IL10RA	interleukin 10 receptor, alpha	PanCancer Immune Profiling
IL11	interleukin 11	PanCancer Immune Profiling
IL11RA	interleukin 11 receptor, alpha	PanCancer Immune Profiling
IL12A	interleukin 12A (natural killer cell stimulatory factor 1, cytotoxic lymphocyte maturation factor 1, p35)	PanCancer Immune Profiling
IL12B	interleukin 12B (natural killer cell stimulatory factor 2, cytotoxic lymphocyte maturation factor 2, p40)	PanCancer Immune Profiling
IL12RB1	interleukin 12 receptor, beta 1	PanCancer Immune Profiling
IL12RB2	interleukin 12 receptor, beta 2	PanCancer Immune Profiling
IL13	interleukin 13	PanCancer Immune Profiling
IL13RA1	interleukin 13 receptor, alpha 1	PanCancer Immune Profiling
IL13RA2	interleukin 13 receptor, alpha 2	PanCancer Immune Profiling
IL15	interleukin 15	PanCancer Immune Profiling
IL15RA	interleukin 15 receptor, alpha	PanCancer Immune Profiling
IL16	interleukin 16	PanCancer Immune Profiling
IL17A	interleukin 17A	PanCancer Immune Profiling
IL17B	interleukin 17B	PanCancer Immune Profiling

IL17F	interleukin 17F	PanCancer Immune Profiling
IL17RA	interleukin 17 receptor A	PanCancer Immune Profiling
IL17RB	interleukin 17 receptor B	PanCancer Immune Profiling
IL18	interleukin 18 (interferon-gamma-inducing factor)	PanCancer Immune Profiling
IL18R1	interleukin 18 receptor 1	PanCancer Immune Profiling
IL18RAP	interleukin 18 receptor accessory protein	PanCancer Immune Profiling
IL19	interleukin 19	PanCancer Immune Profiling
IL1A	interleukin 1, alpha	PanCancer Immune Profiling
IL1B	interleukin 1, beta	PanCancer Immune Profiling
IL1R1	interleukin 1 receptor, type I	PanCancer Immune Profiling
IL1R2	interleukin 1 receptor, type II	PanCancer Immune Profiling
IL1RAP	interleukin 1 receptor accessory protein	PanCancer Immune Profiling
IL1RAPL2	interleukin 1 receptor accessory protein-like 2	PanCancer Immune Profiling
IL1RL1	interleukin 1 receptor-like 1	PanCancer Immune Profiling
IL1RL2	interleukin 1 receptor-like 2	PanCancer Immune Profiling

IL1RN	interleukin 1 receptor antagonist	PanCancer Immune Profiling
IL2	interleukin 2	PanCancer Immune Profiling
IL21	interleukin 21	PanCancer Immune Profiling
IL21R	interleukin 21 receptor	PanCancer Immune Profiling
IL22	interleukin 22	PanCancer Immune Profiling
IL22RA1	interleukin 22 receptor, alpha 1	PanCancer Immune Profiling
IL22RA2	interleukin 22 receptor, alpha 2	PanCancer Immune Profiling
IL23A	interleukin 23, alpha subunit p19	PanCancer Immune Profiling
IL23R	interleukin 23 receptor	PanCancer Immune Profiling
IL24	interleukin 24	PanCancer Immune Profiling
IL25	interleukin 25	PanCancer Immune Profiling
IL26	interleukin 26	PanCancer Immune Profiling
IL27	interleukin 27	PanCancer Immune Profiling
IL2RA	interleukin 2 receptor, alpha	PanCancer Immune Profiling
IL2RB	interleukin 2 receptor, beta	PanCancer Immune Profiling

IL2RG	interleukin 2 receptor, gamma	PanCancer Immune Profiling
IL3	interleukin 3 (colony-stimulating factor, multiple)	PanCancer Immune Profiling
IL32	interleukin 32	PanCancer Immune Profiling
IL34	interleukin 34	PanCancer Immune Profiling
IL3RA	interleukin 3 receptor, alpha (low affinity)	PanCancer Immune Profiling
IL4	interleukin 4	PanCancer Immune Profiling
IL4R	interleukin 4 receptor	PanCancer Immune Profiling
IL5	interleukin 5 (colony-stimulating factor, eosinophil)	PanCancer Immune Profiling
IL5RA	interleukin 5 receptor, alpha	PanCancer Immune Profiling
IL6	interleukin 6 (interferon, beta 2)	PanCancer Immune Profiling
IL6R	interleukin 6 receptor	PanCancer Immune Profiling
IL6ST	interleukin 6 signal transducer (gp130, oncostatin M receptor)	PanCancer Immune Profiling
IL7	interleukin 7	PanCancer Immune Profiling
IL7R	interleukin 7 receptor	PanCancer Immune Profiling
IL9	interleukin 9	PanCancer Immune Profiling

ILF3	interleukin enhancer binding factor 3, 90kDa	PanCancer Immune Profiling
INPP5D	inositol polyphosphate-5-phosphatase, 145kDa	PanCancer Immune Profiling
IRAK1	interleukin-1 receptor-associated kinase 1	PanCancer Immune Profiling
IRAK2	interleukin-1 receptor-associated kinase 2	PanCancer Immune Profiling
IRAK4	interleukin-1 receptor-associated kinase 4	PanCancer Immune Profiling
IRF1	interferon regulatory factor 1	PanCancer Immune Profiling
IRF2	interferon regulatory factor 2	PanCancer Immune Profiling
IRF3	interferon regulatory factor 3	PanCancer Immune Profiling
IRF4	interferon regulatory factor 4	PanCancer Immune Profiling
IRF5	interferon regulatory factor 5	PanCancer Immune Profiling
IRF7	interferon regulatory factor 7	PanCancer Immune Profiling
IRF8	interferon regulatory factor 8	PanCancer Immune Profiling
IRGM	immunity-related GTPase family, M	PanCancer Immune Profiling
ISG15	ISG15 ubiquitin-like modifier	PanCancer Immune Profiling
ISG20	interferon stimulated exonuclease gene 20kDa	PanCancer Immune Profiling

ITCH	itchy E3 ubiquitin protein ligase	PanCancer Immune Profiling
ITGA1	integrin, alpha 1	PanCancer Immune Profiling
ITGA2	integrin, alpha 2 (CD49B, alpha 2 subunit of VLA-2 receptor)	PanCancer Immune Profiling
ITGA2B	integrin, alpha 2b (platelet glycoprotein IIb of IIb/IIIa complex, antigen CD41)	PanCancer Immune Profiling
ITGA4	integrin, alpha 4 (antigen CD49D, alpha 4 subunit of VLA-4 receptor)	PanCancer Immune Profiling
ITGA5	integrin, alpha 5 (fibronectin receptor, alpha polypeptide)	PanCancer Immune Profiling
ITGA6	integrin, alpha 6	PanCancer Immune Profiling
ITGAE	integrin, alpha E (antigen CD103, human mucosal lymphocyte antigen 1; alpha polypeptide)	PanCancer Immune Profiling
ITGAL	integrin, alpha L (antigen CD11A (p180), lymphocyte function-associated antigen 1; alpha polypeptide)	PanCancer Immune Profiling
ITGAM	integrin, alpha M (complement component 3 receptor 3 subunit)	PanCancer Immune Profiling
ITGAX	integrin, alpha X (complement component 3 receptor 4 subunit)	PanCancer Immune Profiling
ITGB1	integrin, beta 1 (fibronectin receptor, beta polypeptide, antigen CD29 includes MDF2, MSK12)	PanCancer Immune Profiling
ITGB2	integrin, beta 2 (complement component 3 receptor 3 and 4 subunit)	PanCancer Immune Profiling
ITGB3	integrin, beta 3 (platelet glycoprotein IIIa, antigen CD61)	PanCancer Immune Profiling
ITGB4	integrin, beta 4	PanCancer Immune Profiling

ITK	IL2-inducible T-cell kinase	PanCancer Immune Profiling
JAK1	Janus kinase 1	PanCancer Immune Profiling
JAK2	Janus kinase 2	PanCancer Immune Profiling
JAK3	Janus kinase 3	PanCancer Immune Profiling
JAM3	junctional adhesion molecule 3	PanCancer Immune Profiling
KIR2DL1	killer cell immunoglobulin-like receptor, two domains, long cytoplasmic tail, 1	PanCancer Immune Profiling
KIR2DL3	killer cell immunoglobulin-like receptor, two domains, long cytoplasmic tail, 3	PanCancer Immune Profiling
KIR2DS1	killer cell immunoglobulin-like receptor, two domains, short cytoplasmic tail, 1	PanCancer Immune Profiling
KIR3DL1	killer cell immunoglobulin-like receptor, three domains, long cytoplasmic tail, 1	PanCancer Immune Profiling
KIR3DL2	killer cell immunoglobulin-like receptor, three domains, long cytoplasmic tail, 2	PanCancer Immune Profiling
KIR3DL3	killer cell immunoglobulin-like receptor, three domains, long cytoplasmic tail, 3	PanCancer Immune Profiling
KIR3DS1	killer cell immunoglobulin-like receptor, three domains, short cytoplasmic tail, 1	PanCancer Immune Profiling
KIT	v-kit Hardy-Zuckerman 4 feline sarcoma viral oncogene homolog	PanCancer Immune Profiling
KLRB1	killer cell lectin-like receptor subfamily B, member 1	PanCancer Immune Profiling
KLRC1	killer cell lectin-like receptor subfamily C, member 1	PanCancer Immune Profiling

KLRC2	killer cell lectin-like receptor subfamily C, member 2	PanCancer Immune Profiling
KLRD1	killer cell lectin-like receptor subfamily D, member 1	PanCancer Immune Profiling
KLRF1	killer cell lectin-like receptor subfamily F, member 1	PanCancer Immune Profiling
KLRG1	killer cell lectin-like receptor subfamily G, member 1	PanCancer Immune Profiling
KLRK1	killer cell lectin-like receptor subfamily K, member 1	PanCancer Immune Profiling
LAG3	lymphocyte-activation gene 3	PanCancer Immune Profiling
LAIR2	leukocyte-associated immunoglobulin-like receptor 2	PanCancer Immune Profiling
LAMP1	lysosomal-associated membrane protein 1	PanCancer Immune Profiling
LAMP2	lysosomal-associated membrane protein 2	PanCancer Immune Profiling
LAMP3	lysosomal-associated membrane protein 3	PanCancer Immune Profiling
LBP	lipopolysaccharide binding protein	PanCancer Immune Profiling
LCK	lymphocyte-specific protein tyrosine kinase	PanCancer Immune Profiling
LCN2	lipocalin 2	PanCancer Immune Profiling
LCP1	lymphocyte cytosolic protein 1 (L-plastin)	PanCancer Immune Profiling
LGALS3	lectin, galactoside-binding, soluble, 3	PanCancer Immune Profiling

LIF	leukemia inhibitory factor	PanCancer Immune Profiling
LILRA1	leukocyte immunoglobulin-like receptor, subfamily A (with TM domain), member 1	PanCancer Immune Profiling
LILRA4	leukocyte immunoglobulin-like receptor, subfamily A (with TM domain), member 4	PanCancer Immune Profiling
LILRA5	leukocyte immunoglobulin-like receptor, subfamily A (with TM domain), member 5	PanCancer Immune Profiling
LILRB1	leukocyte immunoglobulin-like receptor, subfamily B (with TM and ITIM domains), member 1	PanCancer Immune Profiling
LILRB2	leukocyte immunoglobulin-like receptor, subfamily B (with TM and ITIM domains), member 2	PanCancer Immune Profiling
LILRB3	leukocyte immunoglobulin-like receptor, subfamily B (with TM and ITIM domains), member 3	PanCancer Immune Profiling
LRP1	low density lipoprotein receptor-related protein 1	PanCancer Immune Profiling
LRRN3	leucine rich repeat neuronal 3	PanCancer Immune Profiling
LTA	lymphotoxin alpha (TNF superfamily, member 1)	PanCancer Immune Profiling
LTB	lymphotoxin beta (TNF superfamily, member 3)	PanCancer Immune Profiling
LTBR	lymphotoxin beta receptor (TNFR superfamily, member 3)	PanCancer Immune Profiling
LTF	lactotransferrin	PanCancer Immune Profiling
LTK	leukocyte receptor tyrosine kinase	PanCancer Immune Profiling
LY86	lymphocyte antigen 86	PanCancer Immune Profiling

LY9	lymphocyte antigen 9	PanCancer Immune Profiling
LY96	lymphocyte antigen 96	PanCancer Immune Profiling
LYN	v-yes-1 Yamaguchi sarcoma viral related oncogene homolog	PanCancer Immune Profiling
MAF	v-maf musculoaponeurotic fibrosarcoma oncogene homolog (avian)	PanCancer Immune Profiling
MAGEA1	melanoma antigen family A, 1 (directs expression of antigen MZ2-E)	PanCancer Immune Profiling
MAGEA12	melanoma antigen family A, 12	PanCancer Immune Profiling
MAGEA3	melanoma antigen family A, 3	PanCancer Immune Profiling
MAGEA4	melanoma antigen family A, 4	PanCancer Immune Profiling
MAGEB2	melanoma antigen family B, 2	PanCancer Immune Profiling
MAGEC1	melanoma antigen family C, 1	PanCancer Immune Profiling
MAGEC2	melanoma antigen family C, 2	PanCancer Immune Profiling
MAP2K1	mitogen-activated protein kinase kinase 1	PanCancer Immune Profiling
MAP2K2	mitogen-activated protein kinase kinase 2	PanCancer Immune Profiling
MAP2K4	mitogen-activated protein kinase kinase 4	PanCancer Immune Profiling
MAP3K1	mitogen-activated protein kinase kinase kinase 1, E3 ubiquitin protein ligase	PanCancer Immune Profiling

MAP3K5	mitogen-activated protein kinase kinase kinase 5	PanCancer Immune Profiling
MAP3K7	mitogen-activated protein kinase kinase kinase 7	PanCancer Immune Profiling
MAP4K2	mitogen-activated protein kinase kinase kinase kinase 2	PanCancer Immune Profiling
MAPK1	mitogen-activated protein kinase 1	PanCancer Immune Profiling
MAPK11	mitogen-activated protein kinase 11	PanCancer Immune Profiling
MAPK14	mitogen-activated protein kinase 14	PanCancer Immune Profiling
MAPK3	mitogen-activated protein kinase 3	PanCancer Immune Profiling
MAPK8	mitogen-activated protein kinase 8	PanCancer Immune Profiling
MAPKAPK 2	mitogen-activated protein kinase-activated protein kinase 2	PanCancer Immune Profiling
MARCO	macrophage receptor with collagenous structure	PanCancer Immune Profiling
MASP1	mannan-binding lectin serine peptidase 1 (C4/C2 activating component of Ra-reactive factor)	PanCancer Immune Profiling
MASP2	mannan-binding lectin serine peptidase 2	PanCancer Immune Profiling
MAVS	mitochondrial antiviral signaling protein	PanCancer Immune Profiling
MBL2	mannose-binding lectin (protein C) 2, soluble	PanCancer Immune Profiling
MCAM	melanoma cell adhesion molecule	PanCancer Immune Profiling

MEF2C	myocyte enhancer factor 2C	PanCancer Immune Profiling
MEFV	Mediterranean fever	PanCancer Immune Profiling
MERTK	c-mer proto-oncogene tyrosine kinase	PanCancer Immune Profiling
MFGE8	milk fat globule-EGF factor 8 protein	PanCancer Immune Profiling
MICA	MHC class I polypeptide-related sequence A	PanCancer Immune Profiling
MICB	MHC class I polypeptide-related sequence B	PanCancer Immune Profiling
MIF	macrophage migration inhibitory factor (glycosylation-inhibiting factor)	PanCancer Immune Profiling
MME	membrane metallo-endopeptidase	PanCancer Immune Profiling
MNX1	motor neuron and pancreas homeobox 1	PanCancer Immune Profiling
MPPED1	metallophosphoesterase domain containing 1	PanCancer Immune Profiling
MR1	major histocompatibility complex, class I-related	PanCancer Immune Profiling
MRC1	mannose receptor, C type 1	PanCancer Immune Profiling
MS4A1	membrane-spanning 4-domains, subfamily A, member 1	PanCancer Immune Profiling
MS4A2	membrane-spanning 4-domains, subfamily A, member 2	PanCancer Immune Profiling
MSR1	macrophage scavenger receptor 1	PanCancer Immune Profiling

MST1R	macrophage stimulating 1 receptor (c-met-related tyrosine kinase)	PanCancer Immune Profiling
MUC1	mucin 1, cell surface associated	PanCancer Immune Profiling
MX1	myxovirus (influenza virus) resistance 1, interferon-inducible protein p78 (mouse)	PanCancer Immune Profiling
MYD88	myeloid differentiation primary response gene (88)	PanCancer Immune Profiling
NCAM1	neural cell adhesion molecule 1	PanCancer Immune Profiling
NCF4	neutrophil cytosolic factor 4, 40kDa	PanCancer Immune Profiling
NCR1	natural cytotoxicity triggering receptor 1	PanCancer Immune Profiling
NEFL	neurofilament, light polypeptide	PanCancer Immune Profiling
NFATC1	nuclear factor of activated T-cells, cytoplasmic, calcineurin-dependent 1	PanCancer Immune Profiling
NFATC2	nuclear factor of activated T-cells, cytoplasmic, calcineurin-dependent 2	PanCancer Immune Profiling
NFATC3	nuclear factor of activated T-cells, cytoplasmic, calcineurin-dependent 3	PanCancer Immune Profiling
NFATC4	nuclear factor of activated T-cells, cytoplasmic, calcineurin-dependent 4	PanCancer Immune Profiling
NFKB1	nuclear factor of kappa light polypeptide gene enhancer in B-cells 1	PanCancer Immune Profiling
NFKB2	nuclear factor of kappa light polypeptide gene enhancer in B-cells 2 (p49/p100)	PanCancer Immune Profiling
NFKBIA	nuclear factor of kappa light polypeptide gene enhancer in B-cells inhibitor, alpha	PanCancer Immune Profiling

NLRC5	NLR family, CARD domain containing 5	PanCancer Immune Profiling
NLRP3	NLR family, pyrin domain containing 3	PanCancer Immune Profiling
NOD1	nucleotide-binding oligomerization domain containing 1	PanCancer Immune Profiling
NOD2	nucleotide-binding oligomerization domain containing 2	PanCancer Immune Profiling
NOS2A	nitric oxide synthase 2	PanCancer Immune Profiling
NOTCH1	notch 1	PanCancer Immune Profiling
NRP1	neuropilin 1	PanCancer Immune Profiling
NT5E	5'-nucleotidase, ecto (CD73)	PanCancer Immune Profiling
NUP107	nucleoporin 107kDa	PanCancer Immune Profiling
OAS3	2'-5'-oligoadenylate synthetase 3, 100kDa	PanCancer Immune Profiling
OSM	oncostatin M	PanCancer Immune Profiling
PASD1	PAS domain containing 1	PanCancer Immune Profiling
PAX5	paired box 5	PanCancer Immune Profiling
PBK	PDZ binding kinase	PanCancer Immune Profiling
PDCD1	programmed cell death 1	PanCancer Immune Profiling

PDCD1LG2	programmed cell death 1 ligand 2	PanCancer Immune Profiling
PDGFC	platelet derived growth factor C	PanCancer Immune Profiling
PDGFRB	platelet-derived growth factor receptor, beta polypeptide	PanCancer Immune Profiling
PECAM1	platelet/endothelial cell adhesion molecule 1	PanCancer Immune Profiling
PIK3CD	phosphatidylinositol-4,5-bisphosphate 3-kinase, catalytic subunit delta	PanCancer Immune Profiling
PIK3CG	phosphatidylinositol-4,5-bisphosphate 3-kinase, catalytic subunit gamma	PanCancer Immune Profiling
PIN1	peptidylprolyl cis/trans isomerase, NIMA-interacting 1	PanCancer Immune Profiling
PLA2G1B	phospholipase A2, group IB (pancreas)	PanCancer Immune Profiling
PLA2G6	phospholipase A2, group VI (cytosolic, calcium-independent)	PanCancer Immune Profiling
PLAU	plasminogen activator, urokinase	PanCancer Immune Profiling
PLAUR	plasminogen activator, urokinase receptor	PanCancer Immune Profiling
PMCH	pro-melanin-concentrating hormone	PanCancer Immune Profiling
PNMA1	paraneoplastic Ma antigen 1	PanCancer Immune Profiling
POU2AF1	POU class 2 associating factor 1	PanCancer Immune Profiling
POU2F2	POU class 2 homeobox 2	PanCancer Immune Profiling

PPARG	peroxisome proliferator-activated receptor gamma	PanCancer Immune Profiling
PPBP	pro-platelet basic protein (chemokine (C-X-C motif) ligand 7)	PanCancer Immune Profiling
PRAME	preferentially expressed antigen in melanoma	PanCancer Immune Profiling
PRF1	perforin 1 (pore forming protein)	PanCancer Immune Profiling
PRG2	proteoglycan 2, bone marrow (natural killer cell activator, eosinophil granule major basic protein)	PanCancer Immune Profiling
PRKCD	protein kinase C, delta	PanCancer Immune Profiling
PRKCE	protein kinase C, epsilon	PanCancer Immune Profiling
PRM1	protamine 1	PanCancer Immune Profiling
PSEN1	presenilin 1	PanCancer Immune Profiling
PSEN2	presenilin 2 (Alzheimer disease 4)	PanCancer Immune Profiling
PSMB10	proteasome (prosome, macropain) subunit, beta type, 10	PanCancer Immune Profiling
PSMB7	proteasome (prosome, macropain) subunit, beta type, 7	PanCancer Immune Profiling
PSMB8	proteasome (prosome, macropain) subunit, beta type, 8 (large multifunctional peptidase 7)	PanCancer Immune Profiling
PSMB9	proteasome (prosome, macropain) subunit, beta type, 9 (large multifunctional peptidase 2)	PanCancer Immune Profiling
PSMD7	proteasome (prosome, macropain) 26S subunit, non-ATPase, 7	PanCancer Immune Profiling

PTGDR2	prostaglandin D2 receptor 2	PanCancer Immune Profiling
PTGS2	prostaglandin-endoperoxide synthase 2 (prostaglandin G/H synthase and cyclooxygenase)	PanCancer Immune Profiling
PTPRC	protein tyrosine phosphatase, receptor type, C	PanCancer Immune Profiling
PVR	poliovirus receptor	PanCancer Immune Profiling
PYCARD	PYD and CARD domain containing	PanCancer Immune Profiling
RAG1	recombination activating gene 1	PanCancer Immune Profiling
REL	v-rel reticuloendotheliosis viral oncogene homolog (avian)	PanCancer Immune Profiling
RELA	v-rel reticuloendotheliosis viral oncogene homolog A (avian)	PanCancer Immune Profiling
RELB	v-rel reticuloendotheliosis viral oncogene homolog B	PanCancer Immune Profiling
REPS1	RALBP1 associated Eps domain containing 1	PanCancer Immune Profiling
RIPK2	receptor-interacting serine-threonine kinase 2	PanCancer Immune Profiling
ROPN1	rhophilin associated tail protein 1	PanCancer Immune Profiling
RORA	RAR-related orphan receptor A	PanCancer Immune Profiling
RORC	RAR-related orphan receptor C	PanCancer Immune Profiling
RPS6	ribosomal protein S6	PanCancer Immune Profiling

RRAD	Ras-related associated with diabetes	PanCancer Immune Profiling
RUNX1	runt-related transcription factor 1	PanCancer Immune Profiling
RUNX3	runt-related transcription factor 3	PanCancer Immune Profiling
S100A12	S100 calcium binding protein A12	PanCancer Immune Profiling
S100A7	S100 calcium binding protein A7	PanCancer Immune Profiling
S100A8	S100 calcium binding protein A8	PanCancer Immune Profiling
S100B	S100 calcium binding protein B	PanCancer Immune Profiling
SAA1	serum amyloid A1	PanCancer Immune Profiling
SBNO2	strawberry notch homolog 2 (Drosophila)	PanCancer Immune Profiling
SELE	selectin E	PanCancer Immune Profiling
SELL	selectin L	PanCancer Immune Profiling
SELPLG	selectin P ligand	PanCancer Immune Profiling
SEMG1	semenogelin I	PanCancer Immune Profiling
SERPINB2	serpin peptidase inhibitor, clade B (ovalbumin), member 2	PanCancer Immune Profiling
SERPING1	serpin peptidase inhibitor, clade G (C1 inhibitor), member 1	PanCancer Immune Profiling

SH2B2	SH2B adaptor protein 2	PanCancer Immune Profiling
SH2D1A	SH2 domain containing 1A	PanCancer Immune Profiling
SH2D1B	SH2 domain containing 1B	PanCancer Immune Profiling
SIGIRR	single immunoglobulin and toll-interleukin 1 receptor (TIR) domain	PanCancer Immune Profiling
SIGLEC1	sialic acid binding Ig-like lectin 1, sialoadhesin	PanCancer Immune Profiling
SLAMF1	signaling lymphocytic activation molecule family member 1	PanCancer Immune Profiling
SLAMF6	SLAM family member 6	PanCancer Immune Profiling
SLAMF7	SLAM family member 7	PanCancer Immune Profiling
SLC11A1	solute carrier family 11 (proton-coupled divalent metal ion transporters), member 1	PanCancer Immune Profiling
SMAD2	SMAD family member 2	PanCancer Immune Profiling
SMAD3	SMAD family member 3	PanCancer Immune Profiling
SMPD3	sphingomyelin phosphodiesterase 3, neutral membrane (neutral sphingomyelinase II)	PanCancer Immune Profiling
SOCS1	suppressor of cytokine signaling 1	PanCancer Immune Profiling
SPA17	sperm autoantigenic protein 17	PanCancer Immune Profiling
SPACA3	sperm acrosome associated 3	PanCancer Immune Profiling

SPANXB1	SPANX family, member B1	PanCancer Immune Profiling
SPINK5	serine peptidase inhibitor, Kazal type 5	PanCancer Immune Profiling
SPN	sialophorin	PanCancer Immune Profiling
SPO11	SPO11 meiotic protein covalently bound to DSB homolog (S. cerevisiae)	PanCancer Immune Profiling
SPP1	secreted phosphoprotein 1	PanCancer Immune Profiling
SSX1	synovial sarcoma, X breakpoint 1	PanCancer Immune Profiling
SSX4	synovial sarcoma, X breakpoint 4	PanCancer Immune Profiling
ST6GAL1	ST6 beta-galactosamide alpha-2,6-sialyltranferase 1	PanCancer Immune Profiling
STAT1	signal transducer and activator of transcription 1, 91kDa	PanCancer Immune Profiling
STAT2	signal transducer and activator of transcription 2, 113kDa	PanCancer Immune Profiling
STAT3	signal transducer and activator of transcription 3 (acute-phase response factor)	PanCancer Immune Profiling
STAT4	signal transducer and activator of transcription 4	PanCancer Immune Profiling
STAT5B	signal transducer and activator of transcription 5B	PanCancer Immune Profiling
STAT6	signal transducer and activator of transcription 6, interleukin-4 induced	PanCancer Immune Profiling
SYCP1	synaptonemal complex protein 1	PanCancer Immune Profiling

SYK	spleen tyrosine kinase	PanCancer Immune Profiling
SYT17	synaptotagmin XVII	PanCancer Immune Profiling
TAB1	TGF-beta activated kinase 1/MAP3K7 binding protein 1	PanCancer Immune Profiling
TAL1	T-cell acute lymphocytic leukemia 1	PanCancer Immune Profiling
TANK	TRAF family member-associated NFKB activator	PanCancer Immune Profiling
TAP1	transporter 1, ATP-binding cassette, sub-family B (MDR/TAP)	PanCancer Immune Profiling
TAP2	transporter 2, ATP-binding cassette, sub-family B (MDR/TAP)	PanCancer Immune Profiling
TAPBP	TAP binding protein (tapasin)	PanCancer Immune Profiling
TARP	TCR gamma alternate reading frame protein	PanCancer Immune Profiling
TBK1	TANK-binding kinase 1	PanCancer Immune Profiling
TBX21	T-box 21	PanCancer Immune Profiling
TCF7	transcription factor 7 (T-cell specific, HMG-box)	PanCancer Immune Profiling
TFE3	transcription factor binding to IGHM enhancer 3	PanCancer Immune Profiling
TFEB	transcription factor EB	PanCancer Immune Profiling
TFRC	transferrin receptor (p90, CD71)	PanCancer Immune Profiling

TGFB1	transforming growth factor, beta 1	PanCancer Immune Profiling
TGFB2	transforming growth factor, beta 2	PanCancer Immune Profiling
THBD	thrombomodulin	PanCancer Immune Profiling
THBS1	thrombospondin 1	PanCancer Immune Profiling
THY1	Thy-1 cell surface antigen	PanCancer Immune Profiling
TICAM1	toll-like receptor adaptor molecule 1	PanCancer Immune Profiling
TICAM2	toll-like receptor adaptor molecule 2	PanCancer Immune Profiling
TIGIT	T cell immunoreceptor with Ig and ITIM domains	PanCancer Immune Profiling
TIRAP	toll-interleukin 1 receptor (TIR) domain containing adaptor protein	PanCancer Immune Profiling
TLR1	toll-like receptor 1	PanCancer Immune Profiling
TLR10	toll-like receptor 10	PanCancer Immune Profiling
TLR2	toll-like receptor 2	PanCancer Immune Profiling
TLR3	toll-like receptor 3	PanCancer Immune Profiling
TLR4	toll-like receptor 4	PanCancer Immune Profiling
TLR5	toll-like receptor 5	PanCancer Immune Profiling

TLR6	toll-like receptor 6	PanCancer Immune Profiling
TLR7	toll-like receptor 7	PanCancer Immune Profiling
TLR8	toll-like receptor 8	PanCancer Immune Profiling
TLR9	toll-like receptor 9	PanCancer Immune Profiling
TMEFF2	transmembrane protein with EGF-like and two follistatin-like domains 2	PanCancer Immune Profiling
TNF	tumor necrosis factor	PanCancer Immune Profiling
TNFAIP3	tumor necrosis factor, alpha-induced protein 3	PanCancer Immune Profiling
TNFRSF10B	tumor necrosis factor receptor superfamily, member 10b	PanCancer Immune Profiling
TNFRSF10C	tumor necrosis factor receptor superfamily, member 10c, decoy without an intracellular domain	PanCancer Immune Profiling
TNFRSF11A	tumor necrosis factor receptor superfamily, member 11a, NFKB activator	PanCancer Immune Profiling
TNFRSF11B	tumor necrosis factor receptor superfamily, member 11b	PanCancer Immune Profiling
TNFRSF12A	tumor necrosis factor receptor superfamily, member 12A	PanCancer Immune Profiling
TNFRSF13B	tumor necrosis factor receptor superfamily, member 13B	PanCancer Immune Profiling
TNFRSF13C	tumor necrosis factor receptor superfamily, member 13C	PanCancer Immune Profiling
TNFRSF14	tumor necrosis factor receptor superfamily, member 14	PanCancer Immune Profiling

TNFRSF17	tumor necrosis factor receptor superfamily, member 17	PanCancer Immune Profiling
TNFRSF18	tumor necrosis factor receptor superfamily, member 18	PanCancer Immune Profiling
TNFRSF1A	tumor necrosis factor receptor superfamily, member 1A	PanCancer Immune Profiling
TNFRSF1B	tumor necrosis factor receptor superfamily, member 1B	PanCancer Immune Profiling
TNFRSF4	tumor necrosis factor receptor superfamily, member 4	PanCancer Immune Profiling
TNFRSF8	tumor necrosis factor receptor superfamily, member 8	PanCancer Immune Profiling
TNFRSF9	tumor necrosis factor receptor superfamily, member 9	PanCancer Immune Profiling
TNFSF10	tumor necrosis factor (ligand) superfamily, member 10	PanCancer Immune Profiling
TNFSF11	tumor necrosis factor (ligand) superfamily, member 11	PanCancer Immune Profiling
TNFSF12	tumor necrosis factor (ligand) superfamily, member 12	PanCancer Immune Profiling
TNFSF13	tumor necrosis factor (ligand) superfamily, member 13	PanCancer Immune Profiling
TNFSF13B	tumor necrosis factor (ligand) superfamily, member 13b	PanCancer Immune Profiling
TNFSF14	tumor necrosis factor (ligand) superfamily, member 14	PanCancer Immune Profiling
TNFSF15	tumor necrosis factor (ligand) superfamily, member 15	PanCancer Immune Profiling
TNFSF18	tumor necrosis factor (ligand) superfamily, member 18	PanCancer Immune Profiling

TNFSF4	tumor necrosis factor (ligand) superfamily, member 4	PanCancer Immune Profiling
TNFSF8	tumor necrosis factor (ligand) superfamily, member 8	PanCancer Immune Profiling
TOLLIP	toll interacting protein	PanCancer Immune Profiling
TP53	tumor protein p53	PanCancer Immune Profiling
TPSAB1	tryptase alpha/beta 1	PanCancer Immune Profiling
TPTE	transmembrane phosphatase with tensin homology	PanCancer Immune Profiling
TRAF2	TNF receptor-associated factor 2	PanCancer Immune Profiling
TRAF3	TNF receptor-associated factor 3	PanCancer Immune Profiling
TRAF6	TNF receptor-associated factor 6, E3 ubiquitin protein ligase	PanCancer Immune Profiling
TREM1	triggering receptor expressed on myeloid cells 1	PanCancer Immune Profiling
TREM2	triggering receptor expressed on myeloid cells 2	PanCancer Immune Profiling
TTK	TTK protein kinase	PanCancer Immune Profiling
TXK	TXK tyrosine kinase	PanCancer Immune Profiling
TXNIP	thioredoxin interacting protein	PanCancer Immune Profiling
TYK2	tyrosine kinase 2	PanCancer Immune Profiling

UBC	ubiquitin C	PanCancer Immune Profiling
ULBP2	UL16 binding protein 2	PanCancer Immune Profiling
USP9Y	ubiquitin specific peptidase 9, Y-linked	PanCancer Immune Profiling
VCAM1	vascular cell adhesion molecule 1	PanCancer Immune Profiling
VEGFA	vascular endothelial growth factor A	PanCancer Immune Profiling
VEGFC	vascular endothelial growth factor C	PanCancer Immune Profiling
XCL2	chemokine (C motif) ligand 2	PanCancer Immune Profiling
XCR1	chemokine (C motif) receptor 1	PanCancer Immune Profiling
YTHDF2	YTH domain family, member 2	PanCancer Immune Profiling
ZAP70	zeta-chain (TCR) associated protein kinase 70kDa	PanCancer Immune Profiling
ZNF205	zinc finger protein 205	PanCancer Immune Profiling
ABCF1	ATP-binding cassette, sub-family F (GCN20), member 1	PanCancer Immune Profiling
AGK	acylglycerol kinase	PanCancer Immune Profiling
ALAS1	aminolevulinate, delta-, synthase 1	PanCancer Immune Profiling
AMMECR1L	AMME chromosomal region gene 1-like	PanCancer Immune Profiling

CC2D1B	coiled-coil and C2 domain containing 1B	PanCancer Immune Profiling
CNOT10	CCR4-NOT transcription complex, subunit 10	PanCancer Immune Profiling
CNOT4	CCR4-NOT transcription complex, subunit 4	PanCancer Immune Profiling
COG7	component of oligomeric golgi complex 7	PanCancer Immune Profiling
DDX50	DEAD (Asp-Glu-Ala-Asp) box polypeptide 50	PanCancer Immune Profiling
DHX16	DEAH (Asp-Glu-Ala-His) box polypeptide 16	PanCancer Immune Profiling
DNAJC14	DnaJ (Hsp40) homolog, subfamily C, member 14	PanCancer Immune Profiling
EDC3	enhancer of mRNA decapping 3 homolog (S. cerevisiae)	PanCancer Immune Profiling
EIF2B4	eukaryotic translation initiation factor 2B, subunit 4 delta, 67kDa	PanCancer Immune Profiling
ERCC3	excision repair cross-complementing rodent repair deficiency, complementation group 3	PanCancer Immune Profiling
FCF1	FCF1 small subunit (SSU) processome component homolog (S. cerevisiae)	PanCancer Immune Profiling
G6PD	glucose-6-phosphate dehydrogenase	PanCancer Immune Profiling
GPATCH3	G patch domain containing 3	PanCancer Immune Profiling
GUSB	glucuronidase, beta	PanCancer Immune Profiling
HDAC3	histone deacetylase 3	PanCancer Immune Profiling

HPRT1	hypoxanthine phosphoribosyltransferase 1	PanCancer Immune Profiling
MRPS5	mitochondrial ribosomal protein S5	PanCancer Immune Profiling
MTMR14	myotubularin related protein 14	PanCancer Immune Profiling
NOL7	nucleolar protein 7, 27kDa	PanCancer Immune Profiling
NUBP1	nucleotide binding protein 1	PanCancer Immune Profiling
POLR2A	polymerase (RNA) II (DNA directed) polypeptide A, 220kDa	PanCancer Immune Profiling
PPIA	peptidylprolyl isomerase A (cyclophilin A)	PanCancer Immune Profiling
PRPF38A	PRP38 pre-mRNA processing factor 38 (yeast) domain containing A	PanCancer Immune Profiling
SAP130	Sin3A-associated protein, 130kDa	PanCancer Immune Profiling
SDHA	succinate dehydrogenase complex, subunit A, flavoprotein (Fp)	PanCancer Immune Profiling
SF3A3	splicing factor 3a, subunit 3, 60kDa	PanCancer Immune Profiling
TBP	TATA box binding protein	PanCancer Immune Profiling
TLK2	tousled-like kinase 2	PanCancer Immune Profiling
TMUB2	transmembrane and ubiquitin-like domain containing 2	PanCancer Immune Profiling
TRIM39	tripartite motif containing 39	PanCancer Immune Profiling

TUBB	tubulin, beta class I	PanCancer Immune Profiling
USP39	ubiquitin specific peptidase 39	PanCancer Immune Profiling
ZC3H14	zinc finger CCCH-type containing 14	PanCancer Immune Profiling
ZKSCAN5	zinc finger with KRAB and SCAN domains 5	PanCancer Immune Profiling
ZNF143	zinc finger protein 143	PanCancer Immune Profiling
ZNF346	zinc finger protein 346	PanCancer Immune Profiling
ABL1	c-abl oncogene 1, non-receptor tyrosine kinase	PanCancer Pathways
ACVR1B	activin A receptor, type IB	PanCancer Pathways
ACVR1C	activin A receptor, type IC	PanCancer Pathways
ACVR2A	activin A receptor, type IIA	PanCancer Pathways
AKT1	v-akt murine thymoma viral oncogene homolog 1	PanCancer Pathways
AKT2	v-akt murine thymoma viral oncogene homolog 2	PanCancer Pathways
AKT3	v-akt murine thymoma viral oncogene homolog 3	PanCancer Pathways
ALK	anaplastic lymphoma receptor tyrosine kinase	PanCancer Pathways
ALKBH2	alkB, alkylation repair homolog 2 (E. coli)	PanCancer Pathways
ALKBH3	alkB, alkylation repair homolog 3 (E. coli)	PanCancer Pathways
AMER1	APC membrane recruitment protein 1	PanCancer Pathways
AMH	anti-Mullerian hormone	PanCancer Pathways
ANGPT1	angiopoietin 1	PanCancer Pathways
APC	adenomatous polyposis coli	PanCancer Pathways

APH1B	APH1B gamma secretase subunit	PanCancer Pathways
AR	androgen receptor	PanCancer Pathways
ARID1A	AT rich interactive domain 1A (SWI-like)	PanCancer Pathways
ARID1B	AT rich interactive domain 1B (SWI1-like)	PanCancer Pathways
ARID2	AT rich interactive domain 2 (ARID, RFX-like)	PanCancer Pathways
ARNT2	aryl-hydrocarbon receptor nuclear translocator 2	PanCancer Pathways
ASXL1	additional sex combs like 1 (Drosophila)	PanCancer Pathways
ATM	ataxia telangiectasia mutated	PanCancer Pathways
ATR	ataxia telangiectasia and Rad3 related	PanCancer Pathways
ATRX	alpha thalassemia/mental retardation syndrome X-linked	PanCancer Pathways
AXIN1	axin 1	PanCancer Pathways
AXIN2	axin 2	PanCancer Pathways
B2M	beta-2-microglobulin	PanCancer Pathways
BAD	BCL2-associated agonist of cell death	PanCancer Pathways
BAIAP3	BAI1-associated protein 3	PanCancer Pathways
BAMBI	BMP and activin membrane-bound inhibitor	PanCancer Pathways
BAP1	BRCA1 associated protein-1 (ubiquitin carboxy-terminal hydrolase)	PanCancer Pathways
BAX	BCL2-associated X protein	PanCancer Pathways
BCL2	B-cell CLL/lymphoma 2	PanCancer Pathways
BCL2A1	BCL2-related protein A1	PanCancer Pathways
BCL2L1	BCL2-like 1	PanCancer Pathways
BCOR	BCL6 corepressor	PanCancer Pathways
BDNF	brain-derived neurotrophic factor	PanCancer Pathways

BID	BH3 interacting domain death agonist	PanCancer Pathways
BIRC3	baculoviral IAP repeat containing 3	PanCancer Pathways
BIRC7	baculoviral IAP repeat containing 7	PanCancer Pathways
BMP2	bone morphogenetic protein 2	PanCancer Pathways
BMP4	bone morphogenetic protein 4	PanCancer Pathways
BMP5	bone morphogenetic protein 5	PanCancer Pathways
BMP6	bone morphogenetic protein 6	PanCancer Pathways
BMP7	bone morphogenetic protein 7	PanCancer Pathways
BMP8A	bone morphogenetic protein 8a	PanCancer Pathways
BMPR1B	bone morphogenetic protein receptor, type IB	PanCancer Pathways
BNIP3	BCL2/adenovirus E1B 19kDa interacting protein 3	PanCancer Pathways
BRAF	v-raf murine sarcoma viral oncogene homolog B	PanCancer Pathways
BRCA1	breast cancer 1, early onset	PanCancer Pathways
BRCA2	breast cancer 2, early onset	PanCancer Pathways
BRIP1	BRCA1 interacting protein C-terminal helicase 1	PanCancer Pathways
C19orf40	chromosome 19 open reading frame 40	PanCancer Pathways
CACNA1C	calcium channel, voltage-dependent, L type, alpha 1C subunit	PanCancer Pathways
CACNA1D	calcium channel, voltage-dependent, L type, alpha 1D subunit	PanCancer Pathways
CACNA1E	calcium channel, voltage-dependent, R type, alpha 1E subunit	PanCancer Pathways
CACNA1G	calcium channel, voltage-dependent, T type, alpha 1G subunit	PanCancer Pathways
CACNA1H	calcium channel, voltage-dependent, T type, alpha 1H subunit	PanCancer Pathways
CACNA2D1	calcium channel, voltage-dependent, alpha 2/delta subunit 1	PanCancer Pathways
CACNA2D2	calcium channel, voltage-dependent, alpha 2/delta subunit 2	PanCancer Pathways

CACNA2D3	calcium channel, voltage-dependent, alpha 2/delta subunit 3	PanCancer Pathways
CACNA2D4	calcium channel, voltage-dependent, alpha 2/delta subunit 4	PanCancer Pathways
CACNB2	calcium channel, voltage-dependent, beta 2 subunit	PanCancer Pathways
CACNB3	calcium channel, voltage-dependent, beta 3 subunit	PanCancer Pathways
CACNB4	calcium channel, voltage-dependent, beta 4 subunit	PanCancer Pathways
CACNG1	calcium channel, voltage-dependent, gamma subunit 1	PanCancer Pathways
CACNG4	calcium channel, voltage-dependent, gamma subunit 4	PanCancer Pathways
CACNG6	calcium channel, voltage-dependent, gamma subunit 6	PanCancer Pathways
CALML3	calmodulin-like 3	PanCancer Pathways
CALML5	calmodulin-like 5	PanCancer Pathways
CALML6	calmodulin-like 6	PanCancer Pathways
CAMK2B	calcium/calmodulin-dependent protein kinase II beta	PanCancer Pathways
CAPN2	calpain 2, (m/II) large subunit	PanCancer Pathways
CARD11	caspase recruitment domain family, member 11	PanCancer Pathways
CASP10	caspase 10, apoptosis-related cysteine peptidase	PanCancer Pathways
CASP12	caspase 12 (gene/pseudogene)	PanCancer Pathways
CASP3	caspase 3, apoptosis-related cysteine peptidase	PanCancer Pathways
CASP7	caspase 7, apoptosis-related cysteine peptidase	PanCancer Pathways
CASP8	caspase 8, apoptosis-related cysteine peptidase	PanCancer Pathways
CASP9	caspase 9, apoptosis-related cysteine peptidase	PanCancer Pathways
CBL	Cbl proto-oncogene, E3 ubiquitin protein ligase	PanCancer Pathways
CBLC	Cbl proto-oncogene C, E3 ubiquitin protein ligase	PanCancer Pathways
CCNA1	cyclin A1	PanCancer Pathways

CCNA2	cyclin A2	PanCancer Pathways
CCNB1	cyclin B1	PanCancer Pathways
CCNB3	cyclin B3	PanCancer Pathways
CCND1	cyclin D1	PanCancer Pathways
CCND2	cyclin D2	PanCancer Pathways
CCND3	cyclin D3	PanCancer Pathways
CCNE1	cyclin E1	PanCancer Pathways
CCNE2	cyclin E2	PanCancer Pathways
CCNO	cyclin O	PanCancer Pathways
CCR7	chemokine (C-C motif) receptor 7	PanCancer Pathways
CD14	CD14 molecule	PanCancer Pathways
CD19	CD19 molecule	PanCancer Pathways
CD40	CD40 molecule, TNF receptor superfamily member 5	PanCancer Pathways
CDC14A	cell division cycle 14A	PanCancer Pathways
CDC14B	cell division cycle 14B	PanCancer Pathways
CDC25A	cell division cycle 25A	PanCancer Pathways
CDC25B	cell division cycle 25B	PanCancer Pathways
CDC25C	cell division cycle 25C	PanCancer Pathways
CDC6	cell division cycle 6	PanCancer Pathways
CDC7	cell division cycle 7	PanCancer Pathways
CDH1	cadherin 1, type 1, E-cadherin (epithelial)	PanCancer Pathways
CDK2	cyclin-dependent kinase 2	PanCancer Pathways
CDK4	cyclin-dependent kinase 4	PanCancer Pathways

CDK6	cyclin-dependent kinase 6	PanCancer Pathways
CDKN1A	cyclin-dependent kinase inhibitor 1A (p21, Cip1)	PanCancer Pathways
CDKN1B	cyclin-dependent kinase inhibitor 1B (p27, Kip1)	PanCancer Pathways
CDKN1C	cyclin-dependent kinase inhibitor 1C (p57, Kip2)	PanCancer Pathways
CDKN2A	cyclin-dependent kinase inhibitor 2A	PanCancer Pathways
CDKN2B	cyclin-dependent kinase inhibitor 2B (p15, inhibits CDK4)	PanCancer Pathways
CDKN2C	cyclin-dependent kinase inhibitor 2C (p18, inhibits CDK4)	PanCancer Pathways
CDKN2D	cyclin-dependent kinase inhibitor 2D (p19, inhibits CDK4)	PanCancer Pathways
CEBPA	CCAAT/enhancer binding protein (C/EBP), alpha	PanCancer Pathways
CEBPE	CCAAT/enhancer binding protein (C/EBP), epsilon	PanCancer Pathways
CHAD	chondroadherin	PanCancer Pathways
CHEK1	checkpoint kinase 1	PanCancer Pathways
CHEK2	checkpoint kinase 2	PanCancer Pathways
CHUK	conserved helix-loop-helix ubiquitous kinase	PanCancer Pathways
CIC	capicua transcriptional repressor	PanCancer Pathways
CLCF1	cardiotrophin-like cytokine factor 1	PanCancer Pathways
CNTFR	ciliary neurotrophic factor receptor	PanCancer Pathways
COL11A1	collagen, type XI, alpha 1	PanCancer Pathways
COL11A2	collagen, type XI, alpha 2	PanCancer Pathways
COL1A1	collagen, type I, alpha 1	PanCancer Pathways
COL1A2	collagen, type I, alpha 2	PanCancer Pathways
COL24A1	collagen, type XXIV, alpha 1	PanCancer Pathways
COL27A1	collagen, type XXVII, alpha 1	PanCancer Pathways

COL2A1	collagen, type II, alpha 1	PanCancer Pathways
COL3A1	collagen, type III, alpha 1	PanCancer Pathways
COL4A3	collagen, type IV, alpha 3 (Goodpasture antigen)	PanCancer Pathways
COL4A4	collagen, type IV, alpha 4	PanCancer Pathways
COL4A5	collagen, type IV, alpha 5	PanCancer Pathways
COL4A6	collagen, type IV, alpha 6	PanCancer Pathways
COL5A1	collagen, type V, alpha 1	PanCancer Pathways
COL5A2	collagen, type V, alpha 2	PanCancer Pathways
COL6A6	collagen, type VI, alpha 6	PanCancer Pathways
COMP	cartilage oligomeric matrix protein	PanCancer Pathways
CREB3L1	cAMP responsive element binding protein 3-like 1	PanCancer Pathways
CREB3L3	cAMP responsive element binding protein 3-like 3	PanCancer Pathways
CREB3L4	cAMP responsive element binding protein 3-like 4	PanCancer Pathways
CREB5	cAMP responsive element binding protein 5	PanCancer Pathways
CREBBP	CREB binding protein	PanCancer Pathways
CRLF2	cytokine receptor-like factor 2	PanCancer Pathways
CSF1R	colony stimulating factor 1 receptor	PanCancer Pathways
CSF2	colony stimulating factor 2 (granulocyte-macrophage)	PanCancer Pathways
CSF3	colony stimulating factor 3 (granulocyte)	PanCancer Pathways
CSF3R	colony stimulating factor 3 receptor (granulocyte)	PanCancer Pathways
CTNNB1	catenin (cadherin-associated protein), beta 1, 88kDa	PanCancer Pathways
CUL1	cullin 1	PanCancer Pathways
CXXC4	CXXC finger protein 4	PanCancer Pathways

CYLD	cylindromatosis (turban tumor syndrome)	PanCancer Pathways
DAXX	death-domain associated protein	PanCancer Pathways
DDB2	damage-specific DNA binding protein 2, 48kDa	PanCancer Pathways
DDIT3	DNA-damage-inducible transcript 3	PanCancer Pathways
DDIT4	DNA-damage-inducible transcript 4	PanCancer Pathways
DKK1	dickkopf WNT signaling pathway inhibitor 1	PanCancer Pathways
DKK2	dickkopf WNT signaling pathway inhibitor 2	PanCancer Pathways
DKK4	dickkopf WNT signaling pathway inhibitor 4	PanCancer Pathways
DLL1	delta-like 1 (Drosophila)	PanCancer Pathways
DLL3	delta-like 3 (Drosophila)	PanCancer Pathways
DLL4	delta-like 4 (Drosophila)	PanCancer Pathways
DNMT1	DNA (cytosine-5-)-methyltransferase 1	PanCancer Pathways
DNMT3A	DNA (cytosine-5-)-methyltransferase 3 alpha	PanCancer Pathways
DTX1	deltex 1, E3 ubiquitin ligase	PanCancer Pathways
DTX3	deltex 3, E3 ubiquitin ligase	PanCancer Pathways
DTX4	deltex 4, E3 ubiquitin ligase	PanCancer Pathways
DUSP10	dual specificity phosphatase 10	PanCancer Pathways
DUSP2	dual specificity phosphatase 2	PanCancer Pathways
DUSP4	dual specificity phosphatase 4	PanCancer Pathways
DUSP5	dual specificity phosphatase 5	PanCancer Pathways
DUSP6	dual specificity phosphatase 6	PanCancer Pathways
DUSP8	dual specificity phosphatase 8	PanCancer Pathways
E2F1	E2F transcription factor 1	PanCancer Pathways

E2F5	E2F transcription factor 5, p130-binding	PanCancer Pathways
EFNA1	ephrin-A1	PanCancer Pathways
EFNA2	ephrin-A2	PanCancer Pathways
EFNA3	ephrin-A3	PanCancer Pathways
EFNA5	ephrin-A5	PanCancer Pathways
EGF	epidermal growth factor	PanCancer Pathways
EGFR	epidermal growth factor receptor	PanCancer Pathways
EIF4EBP1	eukaryotic translation initiation factor 4E binding protein 1	PanCancer Pathways
ENDOG	endonuclease G	PanCancer Pathways
EP300	E1A binding protein p300	PanCancer Pathways
EPHA2	EPH receptor A2	PanCancer Pathways
EPO	erythropoietin	PanCancer Pathways
EPOR	erythropoietin receptor	PanCancer Pathways
ERBB2	v-erb-b2 avian erythroblastic leukemia viral oncogene homolog 2	PanCancer Pathways
ERCC2	excision repair cross-complementing rodent repair deficiency, complementation group 2	PanCancer Pathways
ERCC6	excision repair cross-complementing rodent repair deficiency, complementation group 6	PanCancer Pathways
ETS2	v-ets avian erythroblastosis virus E26 oncogene homolog 2	PanCancer Pathways
ETV1	ets variant 1	PanCancer Pathways
ETV4	ets variant 4	PanCancer Pathways
ETV7	ets variant 7	PanCancer Pathways
EYA1	eyes absent homolog 1 (Drosophila)	PanCancer Pathways
EZH2	enhancer of zeste homolog 2 (Drosophila)	PanCancer Pathways
FANCA	Fanconi anemia, complementation group A	PanCancer Pathways

FANCB	Fanconi anemia, complementation group B	PanCancer Pathways
FANCC	Fanconi anemia, complementation group C	PanCancer Pathways
FANCE	Fanconi anemia, complementation group E	PanCancer Pathways
FANCF	Fanconi anemia, complementation group F	PanCancer Pathways
FANCG	Fanconi anemia, complementation group G	PanCancer Pathways
FANCL	Fanconi anemia, complementation group L	PanCancer Pathways
FAS	Fas cell surface death receptor	PanCancer Pathways
FASLG	Fas ligand (TNF superfamily, member 6)	PanCancer Pathways
FBXW7	F-box and WD repeat domain containing 7, E3 ubiquitin protein ligase	PanCancer Pathways
FEN1	flap structure-specific endonuclease 1	PanCancer Pathways
FGF1	fibroblast growth factor 1 (acidic)	PanCancer Pathways
FGF10	fibroblast growth factor 10	PanCancer Pathways
FGF11	fibroblast growth factor 11	PanCancer Pathways
FGF12	fibroblast growth factor 12	PanCancer Pathways
FGF13	fibroblast growth factor 13	PanCancer Pathways
FGF14	fibroblast growth factor 14	PanCancer Pathways
FGF16	fibroblast growth factor 16	PanCancer Pathways
FGF17	fibroblast growth factor 17	PanCancer Pathways
FGF18	fibroblast growth factor 18	PanCancer Pathways
FGF19	fibroblast growth factor 19	PanCancer Pathways
FGF2	fibroblast growth factor 2 (basic)	PanCancer Pathways
FGF20	fibroblast growth factor 20	PanCancer Pathways
FGF21	fibroblast growth factor 21	PanCancer Pathways

FGF22	fibroblast growth factor 22	PanCancer Pathways
FGF23	fibroblast growth factor 23	PanCancer Pathways
FGF3	fibroblast growth factor 3	PanCancer Pathways
FGF4	fibroblast growth factor 4	PanCancer Pathways
FGF5	fibroblast growth factor 5	PanCancer Pathways
FGF6	fibroblast growth factor 6	PanCancer Pathways
FGF7	fibroblast growth factor 7	PanCancer Pathways
FGF8	fibroblast growth factor 8 (androgen-induced)	PanCancer Pathways
FGF9	fibroblast growth factor 9	PanCancer Pathways
FGFR1	fibroblast growth factor receptor 1	PanCancer Pathways
FGFR2	fibroblast growth factor receptor 2	PanCancer Pathways
FGFR3	fibroblast growth factor receptor 3	PanCancer Pathways
FGFR4	fibroblast growth factor receptor 4	PanCancer Pathways
FIGF	c-fos induced growth factor (vascular endothelial growth factor D)	PanCancer Pathways
FLNA	filamin A, alpha	PanCancer Pathways
FLNC	filamin C, gamma	PanCancer Pathways
FLT1	fms-related tyrosine kinase 1	PanCancer Pathways
FLT3	fms-related tyrosine kinase 3	PanCancer Pathways
FN1	fibronectin 1	PanCancer Pathways
FOS	FBJ murine osteosarcoma viral oncogene homolog	PanCancer Pathways
FOSL1	FOS-like antigen 1	PanCancer Pathways
FOXL2	forkhead box L2	PanCancer Pathways
FOXO4	forkhead box O4	PanCancer Pathways

FST	follistatin	PanCancer Pathways
FUBP1	far upstream element (FUSE) binding protein 1	PanCancer Pathways
FUT8	fucosyltransferase 8 (alpha (1,6) fucosyltransferase)	PanCancer Pathways
FZD10	frizzled family receptor 10	PanCancer Pathways
FZD2	frizzled family receptor 2	PanCancer Pathways
FZD3	frizzled family receptor 3	PanCancer Pathways
FZD7	frizzled family receptor 7	PanCancer Pathways
FZD8	frizzled family receptor 8	PanCancer Pathways
FZD9	frizzled family receptor 9	PanCancer Pathways
GADD45A	growth arrest and DNA-damage-inducible, alpha	PanCancer Pathways
GADD45B	growth arrest and DNA-damage-inducible, beta	PanCancer Pathways
GADD45G	growth arrest and DNA-damage-inducible, gamma	PanCancer Pathways
GAS1	growth arrest-specific 1	PanCancer Pathways
GATA1	GATA binding protein 1 (globin transcription factor 1)	PanCancer Pathways
GATA2	GATA binding protein 2	PanCancer Pathways
GATA3	GATA binding protein 3	PanCancer Pathways
GDF6	growth differentiation factor 6	PanCancer Pathways
GHR	growth hormone receptor	PanCancer Pathways
GLI1	GLI family zinc finger 1	PanCancer Pathways
GLI3	GLI family zinc finger 3	PanCancer Pathways
GNA11	guanine nucleotide binding protein (G protein), alpha 11 (Gq class)	PanCancer Pathways
GNAQ	guanine nucleotide binding protein (G protein), q polypeptide	PanCancer Pathways
GNAS	GNAS complex locus	PanCancer Pathways

GNG12	guanine nucleotide binding protein (G protein), gamma 12	PanCancer Pathways
GNG4	guanine nucleotide binding protein (G protein), gamma 4	PanCancer Pathways
GNG7	guanine nucleotide binding protein (G protein), gamma 7	PanCancer Pathways
GNGT1	guanine nucleotide binding protein (G protein), gamma transducing activity polypeptide 1	PanCancer Pathways
GPC4	glypican 4	PanCancer Pathways
GRB2	growth factor receptor-bound protein 2	PanCancer Pathways
GRIA3	glutamate receptor, ionotropic, AMPA 3	PanCancer Pathways
GRIN1	glutamate receptor, ionotropic, N-methyl D-aspartate 1	PanCancer Pathways
GRIN2A	glutamate receptor, ionotropic, N-methyl D-aspartate 2A	PanCancer Pathways
GRIN2B	glutamate receptor, ionotropic, N-methyl D-aspartate 2B	PanCancer Pathways
GSK3B	glycogen synthase kinase 3 beta	PanCancer Pathways
GTF2H3	general transcription factor IIH, polypeptide 3, 34kDa	PanCancer Pathways
GZMB	granzyme B (granzyme 2, cytotoxic T-lymphocyte-associated serine esterase 1)	PanCancer Pathways
H2AFX	H2A histone family, member X	PanCancer Pathways
H3F3A	H3 histone, family 3A	PanCancer Pathways
H3F3C	H3 histone, family 3C	PanCancer Pathways
HDAC1	histone deacetylase 1	PanCancer Pathways
HDAC10	histone deacetylase 10	PanCancer Pathways
HDAC11	histone deacetylase 11	PanCancer Pathways
HDAC2	histone deacetylase 2	PanCancer Pathways
HDAC4	histone deacetylase 4	PanCancer Pathways
HDAC5	histone deacetylase 5	PanCancer Pathways
HDAC6	histone deacetylase 6	PanCancer Pathways

HELLS	helicase, lymphoid-specific	PanCancer Pathways
HES1	hes family bHLH transcription factor 1	PanCancer Pathways
HES5	hes family bHLH transcription factor 5	PanCancer Pathways
HGF	hepatocyte growth factor (hepapoietin A; scatter factor)	PanCancer Pathways
HHEX	hematopoietically expressed homeobox	PanCancer Pathways
HHIP	hedgehog interacting protein	PanCancer Pathways
HIST1H3B	histone cluster 1, H3b	PanCancer Pathways
HIST1H3G	histone cluster 1, H3g	PanCancer Pathways
HIST1H3H	histone cluster 1, H3h	PanCancer Pathways
HMGA1	high mobility group AT-hook 1	PanCancer Pathways
HMGA2	high mobility group AT-hook 2	PanCancer Pathways
HNF1A	HNF1 homeobox A	PanCancer Pathways
HOXA10	homeobox A10	PanCancer Pathways
HOXA11	homeobox A11	PanCancer Pathways
HOXA9	homeobox A9	PanCancer Pathways
HPGD	hydroxyprostaglandin dehydrogenase 15-(NAD)	PanCancer Pathways
HRAS	Harvey rat sarcoma viral oncogene homolog	PanCancer Pathways
HSP90B1	heat shock protein 90kDa beta (Grp94), member 1	PanCancer Pathways
HSPA1A	heat shock 70kDa protein 1A	PanCancer Pathways
HSPA2	heat shock 70kDa protein 2	PanCancer Pathways
HSPA6	heat shock 70kDa protein 6 (HSP70B')	PanCancer Pathways
HSPB1	heat shock 27kDa protein 1	PanCancer Pathways
IBSP	integrin-binding sialoprotein	PanCancer Pathways

ID1	inhibitor of DNA binding 1, dominant negative helix-loop-helix protein	PanCancer Pathways
ID2	inhibitor of DNA binding 2, dominant negative helix-loop-helix protein	PanCancer Pathways
ID4	inhibitor of DNA binding 4, dominant negative helix-loop-helix protein	PanCancer Pathways
IDH1	isocitrate dehydrogenase 1 (NADP+), soluble	PanCancer Pathways
IDH2	isocitrate dehydrogenase 2 (NADP+), mitochondrial	PanCancer Pathways
IFNA17	interferon, alpha 17	PanCancer Pathways
IFNA2	interferon, alpha 2	PanCancer Pathways
IFNA7	interferon, alpha 7	PanCancer Pathways
IFNG	interferon, gamma	PanCancer Pathways
IGF1	insulin-like growth factor 1 (somatomedin C)	PanCancer Pathways
IGF1R	insulin-like growth factor 1 receptor	PanCancer Pathways
IGFBP3	insulin-like growth factor binding protein 3	PanCancer Pathways
IKBKB	inhibitor of kappa light polypeptide gene enhancer in B-cells, kinase beta	PanCancer Pathways
IKBKG	inhibitor of kappa light polypeptide gene enhancer in B-cells, kinase gamma	PanCancer Pathways
IL10	interleukin 10	PanCancer Pathways
IL11	interleukin 11	PanCancer Pathways
IL11RA	interleukin 11 receptor, alpha	PanCancer Pathways
IL12A	interleukin 12A (natural killer cell stimulatory factor 1, cytotoxic lymphocyte maturation factor 1, p35)	PanCancer Pathways
IL12B	interleukin 12B (natural killer cell stimulatory factor 2, cytotoxic lymphocyte maturation factor 2, p40)	PanCancer Pathways
IL12RB2	interleukin 12 receptor, beta 2	PanCancer Pathways
IL13	interleukin 13	PanCancer Pathways
IL13RA2	interleukin 13 receptor, alpha 2	PanCancer Pathways
IL15	interleukin 15	PanCancer Pathways

IL19	interleukin 19	PanCancer Pathways
IL1A	interleukin 1, alpha	PanCancer Pathways
IL1B	interleukin 1, beta	PanCancer Pathways
IL1R1	interleukin 1 receptor, type I	PanCancer Pathways
IL1R2	interleukin 1 receptor, type II	PanCancer Pathways
IL1RAP	interleukin 1 receptor accessory protein	PanCancer Pathways
IL20RA	interleukin 20 receptor, alpha	PanCancer Pathways
IL20RB	interleukin 20 receptor beta	PanCancer Pathways
IL22RA1	interleukin 22 receptor, alpha 1	PanCancer Pathways
IL22RA2	interleukin 22 receptor, alpha 2	PanCancer Pathways
IL23A	interleukin 23, alpha subunit p19	PanCancer Pathways
IL23R	interleukin 23 receptor	PanCancer Pathways
IL24	interleukin 24	PanCancer Pathways
IL2RA	interleukin 2 receptor, alpha	PanCancer Pathways
IL2RB	interleukin 2 receptor, beta	PanCancer Pathways
IL3	interleukin 3 (colony-stimulating factor, multiple)	PanCancer Pathways
IL3RA	interleukin 3 receptor, alpha (low affinity)	PanCancer Pathways
IL5RA	interleukin 5 receptor, alpha	PanCancer Pathways
IL6	interleukin 6 (interferon, beta 2)	PanCancer Pathways
IL6R	interleukin 6 receptor	PanCancer Pathways
IL7	interleukin 7	PanCancer Pathways
IL7R	interleukin 7 receptor	PanCancer Pathways
IL8	interleukin 8	PanCancer Pathways

INHBA	inhibin, beta A	PanCancer Pathways
INHBB	inhibin, beta B	PanCancer Pathways
IRAK2	interleukin-1 receptor-associated kinase 2	PanCancer Pathways
IRAK3	interleukin-1 receptor-associated kinase 3	PanCancer Pathways
IRS1	insulin receptor substrate 1	PanCancer Pathways
ITGA2	integrin, alpha 2 (CD49B, alpha 2 subunit of VLA-2 receptor)	PanCancer Pathways
ITGA3	integrin, alpha 3 (antigen CD49C, alpha 3 subunit of VLA-3 receptor)	PanCancer Pathways
ITGA6	integrin, alpha 6	PanCancer Pathways
ITGA7	integrin, alpha 7	PanCancer Pathways
ITGA8	integrin, alpha 8	PanCancer Pathways
ITGA9	integrin, alpha 9	PanCancer Pathways
ITGB3	integrin, beta 3 (platelet glycoprotein IIIa, antigen CD61)	PanCancer Pathways
ITGB4	integrin, beta 4	PanCancer Pathways
ITGB6	integrin, beta 6	PanCancer Pathways
ITGB7	integrin, beta 7	PanCancer Pathways
ITGB8	integrin, beta 8	PanCancer Pathways
JAG1	jagged 1	PanCancer Pathways
JAG2	jagged 2	PanCancer Pathways
JAK1	Janus kinase 1	PanCancer Pathways
JAK2	Janus kinase 2	PanCancer Pathways
JAK3	Janus kinase 3	PanCancer Pathways
JUN	jun proto-oncogene	PanCancer Pathways
KAT2B	K(lysine) acetyltransferase 2B	PanCancer Pathways

KDM5C	lysine (K)-specific demethylase 5C	PanCancer Pathways
KDM6A	lysine (K)-specific demethylase 6A	PanCancer Pathways
KIT	v-kit Hardy-Zuckerman 4 feline sarcoma viral oncogene homolog	PanCancer Pathways
KITLG	KIT ligand	PanCancer Pathways
KLF4	Kruppel-like factor 4 (gut)	PanCancer Pathways
KMT2C	lysine (K)-specific methyltransferase 2C	PanCancer Pathways
KMT2D	lysine (K)-specific methyltransferase 2D	PanCancer Pathways
KRAS	Kirsten rat sarcoma viral oncogene homolog	PanCancer Pathways
LAMA1	laminin, alpha 1	PanCancer Pathways
LAMA3	laminin, alpha 3	PanCancer Pathways
LAMA5	laminin, alpha 5	PanCancer Pathways
LAMB3	laminin, beta 3	PanCancer Pathways
LAMB4	laminin, beta 4	PanCancer Pathways
LAMC2	laminin, gamma 2	PanCancer Pathways
LAMC3	laminin, gamma 3	PanCancer Pathways
LAT	linker for activation of T cells	PanCancer Pathways
LEF1	lymphoid enhancer-binding factor 1	PanCancer Pathways
LEFTY1	left-right determination factor 1	PanCancer Pathways
LEFTY2	left-right determination factor 2	PanCancer Pathways
LEP	leptin	PanCancer Pathways
LEPR	leptin receptor	PanCancer Pathways
LFNG	LFNG O-fucosylpeptide 3-beta-N-acetylglucosaminyltransferase	PanCancer Pathways
LIF	leukemia inhibitory factor	PanCancer Pathways

LIFR	leukemia inhibitory factor receptor alpha	PanCancer Pathways
LIG4	ligase IV, DNA, ATP-dependent	PanCancer Pathways
LRP2	low density lipoprotein receptor-related protein 2	PanCancer Pathways
LTBP1	latent transforming growth factor beta binding protein 1	PanCancer Pathways
MAD2L2	MAD2 mitotic arrest deficient-like 2 (yeast)	PanCancer Pathways
MAML2	mastermind-like 2 (Drosophila)	PanCancer Pathways
MAP2K1	mitogen-activated protein kinase kinase 1	PanCancer Pathways
MAP2K2	mitogen-activated protein kinase kinase 2	PanCancer Pathways
MAP2K4	mitogen-activated protein kinase kinase 4	PanCancer Pathways
MAP2K6	mitogen-activated protein kinase kinase 6	PanCancer Pathways
MAP3K1	mitogen-activated protein kinase kinase kinase 1, E3 ubiquitin protein ligase	PanCancer Pathways
MAP3K12	mitogen-activated protein kinase kinase kinase 12	PanCancer Pathways
MAP3K13	mitogen-activated protein kinase kinase kinase 13	PanCancer Pathways
MAP3K14	mitogen-activated protein kinase kinase kinase 14	PanCancer Pathways
MAP3K5	mitogen-activated protein kinase kinase kinase 5	PanCancer Pathways
MAP3K8	mitogen-activated protein kinase kinase kinase 8	PanCancer Pathways
MAPK1	mitogen-activated protein kinase 1	PanCancer Pathways
MAPK10	mitogen-activated protein kinase 10	PanCancer Pathways
MAPK12	mitogen-activated protein kinase 12	PanCancer Pathways
MAPK3	mitogen-activated protein kinase 3	PanCancer Pathways
MAPK8	mitogen-activated protein kinase 8	PanCancer Pathways
MAPK8IP1	mitogen-activated protein kinase 8 interacting protein 1	PanCancer Pathways
MAPK8IP2	mitogen-activated protein kinase 8 interacting protein 2	PanCancer Pathways

MAPK9	mitogen-activated protein kinase 9	PanCancer Pathways
MAPT	microtubule-associated protein tau	PanCancer Pathways
MCM2	minichromosome maintenance complex component 2	PanCancer Pathways
MCM4	minichromosome maintenance complex component 4	PanCancer Pathways
MCM5	minichromosome maintenance complex component 5	PanCancer Pathways
MCM7	minichromosome maintenance complex component 7	PanCancer Pathways
MDC1	mediator of DNA-damage checkpoint 1	PanCancer Pathways
MDM2	MDM2 oncogene, E3 ubiquitin protein ligase	PanCancer Pathways
MECOM	MDS1 and EVI1 complex locus	PanCancer Pathways
MED12	mediator complex subunit 12	PanCancer Pathways
MEN1	multiple endocrine neoplasia I	PanCancer Pathways
MET	met proto-oncogene	PanCancer Pathways
MFNG	MFNG O-fucosylpeptide 3-beta-N-acetylglucosaminyltransferase	PanCancer Pathways
MGMT	O-6-methylguanine-DNA methyltransferase	PanCancer Pathways
MLF1	myeloid leukemia factor 1	PanCancer Pathways
MLH1	mutL homolog 1	PanCancer Pathways
MLLT3	myeloid/lymphoid or mixed-lineage leukemia (trithorax homolog, Drosophila); translocated to, 3	PanCancer Pathways
MLLT4	myeloid/lymphoid or mixed-lineage leukemia (trithorax homolog, Drosophila); translocated to, 4	PanCancer Pathways
MMP3	matrix metallopeptidase 3 (stromelysin 1, progelatinase)	PanCancer Pathways
MMP7	matrix metallopeptidase 7 (matrilysin, uterine)	PanCancer Pathways
MMP9	matrix metallopeptidase 9 (gelatinase B, 92kDa gelatinase, 92kDa type IV collagenase)	PanCancer Pathways
MNAT1	MNAT CDK-activating kinase assembly factor 1	PanCancer Pathways
MPL	myeloproliferative leukemia virus oncogene	PanCancer Pathways

MPO	myeloperoxidase	PanCancer Pathways
MSH2	mutS homolog 2	PanCancer Pathways
MSH6	mutS homolog 6	PanCancer Pathways
MTOR	mechanistic target of rapamycin (serine/threonine kinase)	PanCancer Pathways
MUTYH	mutY homolog	PanCancer Pathways
MYB	v-myb avian myeloblastosis viral oncogene homolog	PanCancer Pathways
MYC	v-myc avian myelocytomatosis viral oncogene homolog	PanCancer Pathways
MYCN	v-myc avian myelocytomatosis viral oncogene neuroblastoma derived homolog	PanCancer Pathways
MYD88	myeloid differentiation primary response 88	PanCancer Pathways
NASP	nuclear autoantigenic sperm protein (histone-binding)	PanCancer Pathways
NBN	nibrin	PanCancer Pathways
NCOR1	nuclear receptor corepressor 1	PanCancer Pathways
NF1	neurofibromin 1	PanCancer Pathways
NF2	neurofibromin 2 (merlin)	PanCancer Pathways
NFATC1	nuclear factor of activated T-cells, cytoplasmic, calcineurin-dependent 1	PanCancer Pathways
NFE2L2	nuclear factor, erythroid 2-like 2	PanCancer Pathways
NFKB1	nuclear factor of kappa light polypeptide gene enhancer in B-cells 1	PanCancer Pathways
NFKBIA	nuclear factor of kappa light polypeptide gene enhancer in B-cells inhibitor, alpha	PanCancer Pathways
NFKBIZ	nuclear factor of kappa light polypeptide gene enhancer in B-cells inhibitor, zeta	PanCancer Pathways
NGF	nerve growth factor (beta polypeptide)	PanCancer Pathways
NGFR	nerve growth factor receptor	PanCancer Pathways
NKD1	naked cuticle homolog 1 (Drosophila)	PanCancer Pathways
NODAL	nodal growth differentiation factor	PanCancer Pathways

NOG	noggin	PanCancer Pathways
NOS3	nitric oxide synthase 3 (endothelial cell)	PanCancer Pathways
NOTCH1	notch 1	PanCancer Pathways
NOTCH2	notch 2	PanCancer Pathways
NOTCH3	notch 3	PanCancer Pathways
NPM1	nucleophosmin (nucleolar phosphoprotein B23, numatrin)	PanCancer Pathways
NPM2	nucleophosmin/nucleoplasmin 2	PanCancer Pathways
NR4A1	nuclear receptor subfamily 4, group A, member 1	PanCancer Pathways
NR4A3	nuclear receptor subfamily 4, group A, member 3	PanCancer Pathways
NRAS	neuroblastoma RAS viral (v-ras) oncogene homolog	PanCancer Pathways
NSD1	nuclear receptor binding SET domain protein 1	PanCancer Pathways
NTF3	neurotrophin 3	PanCancer Pathways
NTHL1	nth endonuclease III-like 1 (E. coli)	PanCancer Pathways
NTRK1	neurotrophic tyrosine kinase, receptor, type 1	PanCancer Pathways
NTRK2	neurotrophic tyrosine kinase, receptor, type 2	PanCancer Pathways
NUMBL	numb homolog (Drosophila)-like	PanCancer Pathways
NUPR1	nuclear protein, transcriptional regulator, 1	PanCancer Pathways
OSM	oncostatin M	PanCancer Pathways
PAK3	p21 protein (Cdc42/Rac)-activated kinase 3	PanCancer Pathways
PAK7	p21 protein (Cdc42/Rac)-activated kinase 7	PanCancer Pathways
PAX3	paired box 3	PanCancer Pathways
PAX5	paired box 5	PanCancer Pathways
PAX8	paired box 8	PanCancer Pathways

PBRM1	polybromo 1	PanCancer Pathways
PBX1	pre-B-cell leukemia homeobox 1	PanCancer Pathways
PBX3	pre-B-cell leukemia homeobox 3	PanCancer Pathways
PCK1	phosphoenolpyruvate carboxykinase 1 (soluble)	PanCancer Pathways
PCNA	proliferating cell nuclear antigen	PanCancer Pathways
PDGFA	platelet-derived growth factor alpha polypeptide	PanCancer Pathways
PDGFB	platelet-derived growth factor beta polypeptide	PanCancer Pathways
PDGFC	platelet derived growth factor C	PanCancer Pathways
PDGFD	platelet derived growth factor D	PanCancer Pathways
PDGFRA	platelet-derived growth factor receptor, alpha polypeptide	PanCancer Pathways
PDGFRB	platelet-derived growth factor receptor, beta polypeptide	PanCancer Pathways
PGF	placental growth factor	PanCancer Pathways
PHF6	PHD finger protein 6	PanCancer Pathways
PIK3CA	phosphatidylinositol-4,5-bisphosphate 3-kinase, catalytic subunit alpha	PanCancer Pathways
PIK3CB	phosphatidylinositol-4,5-bisphosphate 3-kinase, catalytic subunit beta	PanCancer Pathways
PIK3CD	phosphatidylinositol-4,5-bisphosphate 3-kinase, catalytic subunit delta	PanCancer Pathways
PIK3CG	phosphatidylinositol-4,5-bisphosphate 3-kinase, catalytic subunit gamma	PanCancer Pathways
PIK3R1	phosphoinositide-3-kinase, regulatory subunit 1 (alpha)	PanCancer Pathways
PIK3R2	phosphoinositide-3-kinase, regulatory subunit 2 (beta)	PanCancer Pathways
PIK3R3	phosphoinositide-3-kinase, regulatory subunit 3 (gamma)	PanCancer Pathways
PIK3R5	phosphoinositide-3-kinase, regulatory subunit 5	PanCancer Pathways
PIM1	pim-1 oncogene	PanCancer Pathways
PITX2	paired-like homeodomain 2	PanCancer Pathways

PKMYT1	protein kinase, membrane associated tyrosine/threonine 1	PanCancer Pathways
PLA1A	phospholipase A1 member A	PanCancer Pathways
PLA2G10	phospholipase A2, group X	PanCancer Pathways
PLA2G2A	phospholipase A2, group IIA (platelets, synovial fluid)	PanCancer Pathways
PLA2G3	phospholipase A2, group III	PanCancer Pathways
PLA2G4A	phospholipase A2, group IVA (cytosolic, calcium-dependent)	PanCancer Pathways
PLA2G4C	phospholipase A2, group IVC (cytosolic, calcium-independent)	PanCancer Pathways
PLA2G4E	phospholipase A2, group IVE	PanCancer Pathways
PLA2G4F	phospholipase A2, group IVF	PanCancer Pathways
PLA2G5	phospholipase A2, group V	PanCancer Pathways
PLAT	plasminogen activator, tissue	PanCancer Pathways
PLAU	plasminogen activator, urokinase	PanCancer Pathways
PLCB1	phospholipase C, beta 1 (phosphoinositide-specific)	PanCancer Pathways
PLCB4	phospholipase C, beta 4	PanCancer Pathways
PLCE1	phospholipase C, epsilon 1	PanCancer Pathways
PLCG2	phospholipase C, gamma 2 (phosphatidylinositol-specific)	PanCancer Pathways
PLD1	phospholipase D1, phosphatidylcholine-specific	PanCancer Pathways
PML	promyelocytic leukemia	PanCancer Pathways
POLB	polymerase (DNA directed), beta	PanCancer Pathways
POLD1	polymerase (DNA directed), delta 1, catalytic subunit	PanCancer Pathways
POLD4	polymerase (DNA-directed), delta 4, accessory subunit	PanCancer Pathways
POLE2	polymerase (DNA directed), epsilon 2, accessory subunit	PanCancer Pathways
POLR2D	polymerase (RNA) II (DNA directed) polypeptide D	PanCancer Pathways

POLR2H	polymerase (RNA) II (DNA directed) polypeptide H	PanCancer Pathways
POLR2J	polymerase (RNA) II (DNA directed) polypeptide J, 13.3kDa	PanCancer Pathways
PPARG	peroxisome proliferator-activated receptor gamma	PanCancer Pathways
PPARGC1A	peroxisome proliferator-activated receptor gamma, coactivator 1 alpha	PanCancer Pathways
PPP2CB	protein phosphatase 2, catalytic subunit, beta isozyme	PanCancer Pathways
PPP2R1A	protein phosphatase 2, regulatory subunit A, alpha	PanCancer Pathways
PPP2R2B	protein phosphatase 2, regulatory subunit B, beta	PanCancer Pathways
PPP2R2C	protein phosphatase 2, regulatory subunit B, gamma	PanCancer Pathways
PPP3CA	protein phosphatase 3, catalytic subunit, alpha isozyme	PanCancer Pathways
PPP3CB	protein phosphatase 3, catalytic subunit, beta isozyme	PanCancer Pathways
PPP3CC	protein phosphatase 3, catalytic subunit, gamma isozyme	PanCancer Pathways
PPP3R1	protein phosphatase 3, regulatory subunit B, alpha	PanCancer Pathways
PPP3R2	protein phosphatase 3, regulatory subunit B, beta	PanCancer Pathways
PRDM1	PR domain containing 1, with ZNF domain	PanCancer Pathways
PRKAA2	protein kinase, AMP-activated, alpha 2 catalytic subunit	PanCancer Pathways
PRKACA	protein kinase, cAMP-dependent, catalytic, alpha	PanCancer Pathways
PRKACB	protein kinase, cAMP-dependent, catalytic, beta	PanCancer Pathways
PRKACG	protein kinase, cAMP-dependent, catalytic, gamma	PanCancer Pathways
PRKAR1B	protein kinase, cAMP-dependent, regulatory, type I, beta	PanCancer Pathways
PRKAR2A	protein kinase, cAMP-dependent, regulatory, type II, alpha	PanCancer Pathways
PRKAR2B	protein kinase, cAMP-dependent, regulatory, type II, beta	PanCancer Pathways
PRKCA	protein kinase C, alpha	PanCancer Pathways
PRKCB	protein kinase C, beta	PanCancer Pathways

PRKCG	protein kinase C, gamma	PanCancer Pathways
PRKDC	protein kinase, DNA-activated, catalytic polypeptide	PanCancer Pathways
PRKX	protein kinase, X-linked	PanCancer Pathways
PRL	prolactin	PanCancer Pathways
PRLR	prolactin receptor	PanCancer Pathways
PRMT8	protein arginine methyltransferase 8	PanCancer Pathways
PROM1	prominin 1	PanCancer Pathways
PTCH1	patched 1	PanCancer Pathways
PTCRA	pre T-cell antigen receptor alpha	PanCancer Pathways
PTEN	phosphatase and tensin homolog	PanCancer Pathways
PTPN11	protein tyrosine phosphatase, non-receptor type 11	PanCancer Pathways
PTPN5	protein tyrosine phosphatase, non-receptor type 5 (striatum-enriched)	PanCancer Pathways
PTPRR	protein tyrosine phosphatase, receptor type, R	PanCancer Pathways
PTTG2	pituitary tumor-transforming 2	PanCancer Pathways
RAC1	ras-related C3 botulinum toxin substrate 1 (rho family, small GTP binding protein Rac1)	PanCancer Pathways
RAC2	ras-related C3 botulinum toxin substrate 2 (rho family, small GTP binding protein Rac2)	PanCancer Pathways
RAC3	ras-related C3 botulinum toxin substrate 3 (rho family, small GTP binding protein Rac3)	PanCancer Pathways
RAD21	RAD21 homolog (S. pombe)	PanCancer Pathways
RAD50	RAD50 homolog (S. cerevisiae)	PanCancer Pathways
RAD51	RAD51 recombinase	PanCancer Pathways
RAD52	RAD52 homolog (S. cerevisiae)	PanCancer Pathways
RAF1	v-raf-1 murine leukemia viral oncogene homolog 1	PanCancer Pathways
RASA4	RAS p21 protein activator 4	PanCancer Pathways

RASAL1	RAS protein activator like 1 (GAP1 like)	PanCancer Pathways
RASGRF1	Ras protein-specific guanine nucleotide-releasing factor 1	PanCancer Pathways
RASGRF2	Ras protein-specific guanine nucleotide-releasing factor 2	PanCancer Pathways
RASGRP1	RAS guanyl releasing protein 1 (calcium and DAG-regulated)	PanCancer Pathways
RASGRP2	RAS guanyl releasing protein 2 (calcium and DAG-regulated)	PanCancer Pathways
RB1	retinoblastoma 1	PanCancer Pathways
RBX1	ring-box 1, E3 ubiquitin protein ligase	PanCancer Pathways
RELA	v-rel avian reticuloendotheliosis viral oncogene homolog A	PanCancer Pathways
RELN	reelin	PanCancer Pathways
RET	ret proto-oncogene	PanCancer Pathways
RFC3	replication factor C (activator 1) 3, 38kDa	PanCancer Pathways
RFC4	replication factor C (activator 1) 4, 37kDa	PanCancer Pathways
RHOA	ras homolog family member A	PanCancer Pathways
RIN1	Ras and Rab interactor 1	PanCancer Pathways
RNF43	ring finger protein 43	PanCancer Pathways
RPA3	replication protein A3, 14kDa	PanCancer Pathways
RPS27A	ribosomal protein S27a	PanCancer Pathways
RPS6KA5	ribosomal protein S6 kinase, 90kDa, polypeptide 5	PanCancer Pathways
RPS6KA6	ribosomal protein S6 kinase, 90kDa, polypeptide 6	PanCancer Pathways
RRAS2	related RAS viral (r-ras) oncogene homolog 2	PanCancer Pathways
RUNX1	runt-related transcription factor 1	PanCancer Pathways
RUNX1T1	runt-related transcription factor 1; translocated to, 1 (cyclin D-related)	PanCancer Pathways
RXRG	retinoid X receptor, gamma	PanCancer Pathways

SETBP1	SET binding protein 1	PanCancer Pathways
SETD2	SET domain containing 2	PanCancer Pathways
SF3B1	splicing factor 3b, subunit 1, 155kDa	PanCancer Pathways
SFN	stratifin	PanCancer Pathways
SFRP1	secreted frizzled-related protein 1	PanCancer Pathways
SFRP2	secreted frizzled-related protein 2	PanCancer Pathways
SFRP4	secreted frizzled-related protein 4	PanCancer Pathways
SGK2	serum/glucocorticoid regulated kinase 2	PanCancer Pathways
SHC1	SHC (Src homology 2 domain containing) transforming protein 1	PanCancer Pathways
SHC2	SHC (Src homology 2 domain containing) transforming protein 2	PanCancer Pathways
SHC3	SHC (Src homology 2 domain containing) transforming protein 3	PanCancer Pathways
SHC4	SHC (Src homology 2 domain containing) family, member 4	PanCancer Pathways
SIN3A	SIN3 transcription regulator family member A	PanCancer Pathways
SIRT4	sirtuin 4	PanCancer Pathways
SIX1	SIX homeobox 1	PanCancer Pathways
SKP1	S-phase kinase-associated protein 1	PanCancer Pathways
SKP2	S-phase kinase-associated protein 2, E3 ubiquitin protein ligase	PanCancer Pathways
SMAD2	SMAD family member 2	PanCancer Pathways
SMAD3	SMAD family member 3	PanCancer Pathways
SMAD4	SMAD family member 4	PanCancer Pathways
SMAD9	SMAD family member 9	PanCancer Pathways
SMARCA4	SWI/SNF related, matrix associated, actin dependent regulator of chromatin, subfamily a, member 4	PanCancer Pathways
SMARCB1	SWI/SNF related, matrix associated, actin dependent regulator of chromatin, subfamily b, member 1	PanCancer Pathways

SMC1A	structural maintenance of chromosomes 1A	PanCancer Pathways
SMC1B	structural maintenance of chromosomes 1B	PanCancer Pathways
SMC3	structural maintenance of chromosomes 3	PanCancer Pathways
SMO	smoothened, frizzled family receptor	PanCancer Pathways
SOCS1	suppressor of cytokine signaling 1	PanCancer Pathways
SOCS2	suppressor of cytokine signaling 2	PanCancer Pathways
SOCS3	suppressor of cytokine signaling 3	PanCancer Pathways
SOS1	son of sevenless homolog 1 (Drosophila)	PanCancer Pathways
SOS2	son of sevenless homolog 2 (Drosophila)	PanCancer Pathways
SOST	sclerostin	PanCancer Pathways
SOX17	SRY (sex determining region Y)-box 17	PanCancer Pathways
SOX9	SRY (sex determining region Y)-box 9	PanCancer Pathways
SP1	Sp1 transcription factor	PanCancer Pathways
SPOP	speckle-type POZ protein	PanCancer Pathways
SPP1	secreted phosphoprotein 1	PanCancer Pathways
SPRY1	sprouty homolog 1, antagonist of FGF signaling (Drosophila)	PanCancer Pathways
SPRY2	sprouty homolog 2 (Drosophila)	PanCancer Pathways
SPRY4	sprouty homolog 4 (Drosophila)	PanCancer Pathways
SRSF2	serine/arginine-rich splicing factor 2	PanCancer Pathways
SSX1	synovial sarcoma, X breakpoint 1	PanCancer Pathways
STAG2	stromal antigen 2	PanCancer Pathways
STAT1	signal transducer and activator of transcription 1, 91kDa	PanCancer Pathways
STAT3	signal transducer and activator of transcription 3 (acute-phase response factor)	PanCancer Pathways

STAT4	signal transducer and activator of transcription 4	PanCancer Pathways
STK11	serine/threonine kinase 11	PanCancer Pathways
STMN1	stathmin 1	PanCancer Pathways
SUV39H2	suppressor of variegation 3-9 homolog 2 (Drosophila)	PanCancer Pathways
SYK	spleen tyrosine kinase	PanCancer Pathways
TBL1XR1	transducin (beta)-like 1 X-linked receptor 1	PanCancer Pathways
TCF3	transcription factor 3	PanCancer Pathways
TCF7L1	transcription factor 7-like 1 (T-cell specific, HMG-box)	PanCancer Pathways
TCL1B	T-cell leukemia/lymphoma 1B	PanCancer Pathways
TET2	tet methylcytosine dioxygenase 2	PanCancer Pathways
TFDP1	transcription factor Dp-1	PanCancer Pathways
TGFB1	transforming growth factor, beta 1	PanCancer Pathways
TGFB2	transforming growth factor, beta 2	PanCancer Pathways
TGFB3	transforming growth factor, beta 3	PanCancer Pathways
TGFBR2	transforming growth factor, beta receptor II (70/80kDa)	PanCancer Pathways
THBS1	thrombospondin 1	PanCancer Pathways
THBS4	thrombospondin 4	PanCancer Pathways
THEM4	thioesterase superfamily member 4	PanCancer Pathways
TIAM1	T-cell lymphoma invasion and metastasis 1	PanCancer Pathways
TLR2	toll-like receptor 2	PanCancer Pathways
TLR4	toll-like receptor 4	PanCancer Pathways
TLX1	T-cell leukemia homeobox 1	PanCancer Pathways
TMPRSS2	transmembrane protease, serine 2	PanCancer Pathways

TNC	tenascin C	PanCancer Pathways
TNF	tumor necrosis factor	PanCancer Pathways
TNFAIP3	tumor necrosis factor, alpha-induced protein 3	PanCancer Pathways
TNFRSF10A	tumor necrosis factor receptor superfamily, member 10a	PanCancer Pathways
TNFRSF10B	tumor necrosis factor receptor superfamily, member 10b	PanCancer Pathways
TNFRSF10C	tumor necrosis factor receptor superfamily, member 10c, decoy without an intracellular domain	PanCancer Pathways
TNFRSF10D	tumor necrosis factor receptor superfamily, member 10d, decoy with truncated death domain	PanCancer Pathways
TNFSF10	tumor necrosis factor (ligand) superfamily, member 10	PanCancer Pathways
TNN	tenascin N	PanCancer Pathways
TNR	tenascin R	PanCancer Pathways
TP53	tumor protein p53	PanCancer Pathways
TPO	thyroid peroxidase	PanCancer Pathways
TRAF7	TNF receptor-associated factor 7, E3 ubiquitin protein ligase	PanCancer Pathways
TSC1	tuberous sclerosis 1	PanCancer Pathways
TSHR	thyroid stimulating hormone receptor	PanCancer Pathways
TSLP	thymic stromal lymphopoietin	PanCancer Pathways
TSPAN7	tetraspanin 7	PanCancer Pathways
TTK	TTK protein kinase	PanCancer Pathways
U2AF1	U2 small nuclear RNA auxiliary factor 1	PanCancer Pathways
UBB	ubiquitin B	PanCancer Pathways
UBE2T	ubiquitin-conjugating enzyme E2T (putative)	PanCancer Pathways
UTY	ubiquitously transcribed tetratricopeptide repeat containing, Y-linked	PanCancer Pathways
VEGFA	vascular endothelial growth factor A	PanCancer Pathways

VEGFC	vascular endothelial growth factor C	PanCancer Pathways
VHL	von Hippel-Lindau tumor suppressor, E3 ubiquitin protein ligase	PanCancer Pathways
WEE1	WEE1 G2 checkpoint kinase	PanCancer Pathways
WHSC1	Wolf-Hirschhorn syndrome candidate 1	PanCancer Pathways
WHSC1L1	Wolf-Hirschhorn syndrome candidate 1-like 1	PanCancer Pathways
WIF1	WNT inhibitory factor 1	PanCancer Pathways
WNT10A	wingless-type MMTV integration site family, member 10A	PanCancer Pathways
WNT10B	wingless-type MMTV integration site family, member 10B	PanCancer Pathways
WNT11	wingless-type MMTV integration site family, member 11	PanCancer Pathways
WNT16	wingless-type MMTV integration site family, member 16	PanCancer Pathways
WNT2	wingless-type MMTV integration site family member 2	PanCancer Pathways
WNT2B	wingless-type MMTV integration site family, member 2B	PanCancer Pathways
WNT3	wingless-type MMTV integration site family, member 3	PanCancer Pathways
WNT4	wingless-type MMTV integration site family, member 4	PanCancer Pathways
WNT5A	wingless-type MMTV integration site family, member 5A	PanCancer Pathways
WNT5B	wingless-type MMTV integration site family, member 5B	PanCancer Pathways
WNT6	wingless-type MMTV integration site family, member 6	PanCancer Pathways
WNT7A	wingless-type MMTV integration site family, member 7A	PanCancer Pathways
WNT7B	wingless-type MMTV integration site family, member 7B	PanCancer Pathways
WT1	Wilms tumor 1	PanCancer Pathways
XPA	xeroderma pigmentosum, complementation group A	PanCancer Pathways
XRCC4	X-ray repair complementing defective repair in Chinese hamster cells 4	PanCancer Pathways
ZAK	sterile alpha motif and leucine zipper containing kinase AZK	PanCancer Pathways

ZBTB16	zinc finger and BTB domain containing 16	PanCancer Pathways
ZBTB32	zinc finger and BTB domain containing 32	PanCancer Pathways
ZIC2	Zic family member 2	PanCancer Pathways
ACAD9	acyl-CoA dehydrogenase family, member 9	PanCancer Pathways
AGK	acylglycerol kinase	PanCancer Pathways
AMMECR1L	AMMECR1-like	PanCancer Pathways
C10orf76	chromosome 10 open reading frame 76	PanCancer Pathways
CC2D1B	coiled-coil and C2 domain containing 1B	PanCancer Pathways
CNOT10	CCR4-NOT transcription complex, subunit 10	PanCancer Pathways
CNOT4	CCR4-NOT transcription complex, subunit 4	PanCancer Pathways
COG7	component of oligomeric golgi complex 7	PanCancer Pathways
DDX50	DEAD (Asp-Glu-Ala-Asp) box polypeptide 50	PanCancer Pathways
DHX16	DEAH (Asp-Glu-Ala-His) box polypeptide 16	PanCancer Pathways
DNAJC14	DnaJ (Hsp40) homolog, subfamily C, member 14	PanCancer Pathways
EDC3	enhancer of mRNA decapping 3	PanCancer Pathways
EIF2B4	eukaryotic translation initiation factor 2B, subunit 4 delta, 67kDa	PanCancer Pathways
ERCC3	excision repair cross-complementing rodent repair deficiency, complementation group 3	PanCancer Pathways
FCF1	FCF1 rRNA-processing protein	PanCancer Pathways
FTSJ2	FtsJ RNA methyltransferase homolog 2 (E. coli)	PanCancer Pathways
GPATCH3	G patch domain containing 3	PanCancer Pathways
HDAC3	histone deacetylase 3	PanCancer Pathways
MRPS5	mitochondrial ribosomal protein S5	PanCancer Pathways
MTMR14	myotubularin related protein 14	PanCancer Pathways

NOL7	nucleolar protein 7, 27kDa	PanCancer Pathways
NUBP1	nucleotide binding protein 1	PanCancer Pathways
PIAS1	protein inhibitor of activated STAT, 1	PanCancer Pathways
PIK3R4	phosphoinositide-3-kinase, regulatory subunit 4	PanCancer Pathways
PRPF38A	pre-mRNA processing factor 38A	PanCancer Pathways
RBM45	RNA binding motif protein 45	PanCancer Pathways
SAP130	Sin3A-associated protein, 130kDa	PanCancer Pathways
SF3A3	splicing factor 3a, subunit 3, 60kDa	PanCancer Pathways
SLC4A1AP	solute carrier family 4 (anion exchanger), member 1, adaptor protein	PanCancer Pathways
TLK2	tousled-like kinase 2	PanCancer Pathways
TMUB2	transmembrane and ubiquitin-like domain containing 2	PanCancer Pathways
TRIM39	tripartite motif containing 39	PanCancer Pathways
TTC31	tetratricopeptide repeat domain 31	PanCancer Pathways
USP39	ubiquitin specific peptidase 39	PanCancer Pathways
VPS33B	vacuolar protein sorting 33 homolog B (yeast)	PanCancer Pathways
ZC3H14	zinc finger CCCH-type containing 14	PanCancer Pathways
ZKSCAN5	zinc finger with KRAB and SCAN domains 5	PanCancer Pathways
ZNF143	zinc finger protein 143	PanCancer Pathways
ZNF346	zinc finger protein 346	PanCancer Pathways
ZNF384	zinc finger protein 384	PanCancer Pathways
AAMP	angio associated migratory cell protein	PanCancer Progression
ABI3BP	ABI family member 3 binding protein	PanCancer Progression
ACHE	acetylcholinesterase (Cartwright blood group)	PanCancer Progression

ACTG2	actin, gamma 2, smooth muscle, enteric	PanCancer Progression
ACVR1	activin A receptor type 1	PanCancer Progression
ACVR1C	activin A receptor type 1C	PanCancer Progression
ACVRL1	activin A receptor like type 1	PanCancer Progression
ADAM15	ADAM metallopeptidase domain 15	PanCancer Progression
ADAM17	ADAM metallopeptidase domain 17	PanCancer Progression
ADAM28	ADAM metallopeptidase domain 28	PanCancer Progression
ADAM8	ADAM metallopeptidase domain 8	PanCancer Progression
ADAM9	ADAM metallopeptidase domain 9	PanCancer Progression
ADAMTS1	ADAM metallopeptidase with thrombospondin type 1 motif 1	PanCancer Progression
ADAMTS12	ADAM metallopeptidase with thrombospondin type 1 motif 12	PanCancer Progression
ADAMTS8	ADAM metallopeptidase with thrombospondin type 1 motif 8	PanCancer Progression
ADAP1	ArfGAP with dual PH domains 1	PanCancer Progression
ADD1	adducin 1	PanCancer Progression
ADM2	adrenomedullin 2	PanCancer Progression
ADRA2B	adrenoceptor alpha 2B	PanCancer Progression
AEBP1	AE binding protein 1	PanCancer Progression
AGGF1	angiogenic factor with G-patch and FHA domains 1	PanCancer Progression
AGR2	anterior gradient 2, protein disulphide isomerase family member	PanCancer Progression
AGRN	agrin	PanCancer Progression
AGT	angiotensinogen	PanCancer Progression
AHNAK	AHNAK nucleoprotein	PanCancer Progression
AKAP12	A-kinase anchoring protein 12	PanCancer Progression

AKAP2	A-kinase anchoring protein 2	PanCancer Progression
AKT1	AKT serine/threonine kinase 1	PanCancer Progression
AKT2	AKT serine/threonine kinase 2	PanCancer Progression
AKT3	AKT serine/threonine kinase 3	PanCancer Progression
ALB	albumin	PanCancer Progression
ALDOA	aldolase, fructose-bisphosphate A	PanCancer Progression
ALOX5	arachidonate 5-lipoxygenase	PanCancer Progression
AMH	anti-Mullerian hormone	PanCancer Progression
ANG	angiogenin	PanCancer Progression
ANGPT1	angiopoietin 1	PanCancer Progression
ANGPT2	angiopoietin 2	PanCancer Progression
ANGPTL2	angiopoietin like 2	PanCancer Progression
ANGPTL4	angiopoietin like 4	PanCancer Progression
ANPEP	alanyl aminopeptidase, membrane	PanCancer Progression
ANXA2P2	annexin A2 pseudogene 2	PanCancer Progression
AP1M2	adaptor related protein complex 1 mu 2 subunit	PanCancer Progression
APC	APC, WNT signaling pathway regulator	PanCancer Progression
APOD	apolipoprotein D	PanCancer Progression
APOE	apolipoprotein E	PanCancer Progression
APOH	apolipoprotein H	PanCancer Progression
AQP1	aquaporin 1 (Colton blood group)	PanCancer Progression
ARAP2	ArfGAP with RhoGAP domain, ankyrin repeat and PH domain 2	PanCancer Progression
AREG	amphiregulin	PanCancer Progression

ARHGAP32	Rho GTPase activating protein 32	PanCancer Progression
ARHGDIB	Rho GDP dissociation inhibitor beta	PanCancer Progression
ASPN	asporin	PanCancer Progression
ATPIF1	ATP synthase inhibitory factor subunit 1	PanCancer Progression
B3GNT3	UDP-GlcNAc:betaGal beta-1,3-N-acetylglucosaminyltransferase 3	PanCancer Progression
BAD	BCL2 associated agonist of cell death	PanCancer Progression
BAG2	BCL2 associated athanogene 2	PanCancer Progression
BAI1	BAI1 associated protein 2	PanCancer Progression
BAI3	adhesion G protein-coupled receptor B3	PanCancer Progression
BCAS1	breast carcinoma amplified sequence 1	PanCancer Progression
BGN	biglycan	PanCancer Progression
BICC1	BicC family RNA binding protein 1	PanCancer Progression
BMP4	bone morphogenetic protein 4	PanCancer Progression
BMP5	bone morphogenetic protein 5	PanCancer Progression
BMP7	bone morphogenetic protein 7	PanCancer Progression
BMPER	BMP binding endothelial regulator	PanCancer Progression
BMPR1A	bone morphogenetic protein receptor type 1A	PanCancer Progression
BMPR1B	bone morphogenetic protein receptor type 1B	PanCancer Progression
BMPR2	bone morphogenetic protein receptor type 2	PanCancer Progression
BNC2	basonuclin 2	PanCancer Progression
BRMS1	breast cancer metastasis suppressor 1	PanCancer Progression
BTG1	BTG anti-proliferation factor 1	PanCancer Progression
C1S	complement C1s	PanCancer Progression

C3	complement C3	PanCancer Progression
C3AR1	complement C3a receptor 1	PanCancer Progression
CADM1	cell adhesion molecule 1	PanCancer Progression
CALCRL	calcitonin receptor like receptor	PanCancer Progression
CALD1	caldesmon 1	PanCancer Progression
CAMK2A	calcium/calmodulin dependent protein kinase II alpha	PanCancer Progression
CAMK2B	calcium/calmodulin dependent protein kinase II beta	PanCancer Progression
CAMK2D	calcium/calmodulin dependent protein kinase II delta	PanCancer Progression
CAMP	cathelicidin antimicrobial peptide	PanCancer Progression
CASP8	caspase 8	PanCancer Progression
CAV1	caveolin 1	PanCancer Progression
CBLC	Cbl proto-oncogene C	PanCancer Progression
CCBE1	collagen and calcium binding EGF domains 1	PanCancer Progression
CCDC80	coiled-coil domain containing 80	PanCancer Progression
CCL11	C-C motif chemokine ligand 11	PanCancer Progression
CCL21	C-C motif chemokine ligand 21	PanCancer Progression
CCL5	C-C motif chemokine ligand 5	PanCancer Progression
CCL7	C-C motif chemokine ligand 7	PanCancer Progression
CCL8	C-C motif chemokine ligand 8	PanCancer Progression
CCR2	C-C motif chemokine receptor 2	PanCancer Progression
CCR3	C-C motif chemokine receptor 3	PanCancer Progression
CD163	CD163 molecule	PanCancer Progression
CD24	CD24 molecule	PanCancer Progression

CD2AP	CD2 associated protein	PanCancer Progression
CD34	CD34 molecule	PanCancer Progression
CD36	CD36 molecule	PanCancer Progression
CD44	CD44 molecule (Indian blood group)	PanCancer Progression
CD46	CD46 molecule	PanCancer Progression
CD82	CD82 molecule	PanCancer Progression
CDC42	cell division cycle 42	PanCancer Progression
CDH1	cadherin 1	PanCancer Progression
CDH11	cadherin 11	PanCancer Progression
CDH13	cadherin 13	PanCancer Progression
CDH2	cadherin 2	PanCancer Progression
CDK14	cyclin dependent kinase 14	PanCancer Progression
CDKN1A	cyclin dependent kinase inhibitor 1A	PanCancer Progression
CDKN2A	cyclin dependent kinase inhibitor 2A	PanCancer Progression
CDS1	CDP-diacylglycerol synthase 1	PanCancer Progression
CEACAM1	carcinoembryonic antigen related cell adhesion molecule 1	PanCancer Progression
CEACAM5	carcinoembryonic antigen related cell adhesion molecule 5	PanCancer Progression
CEACAM6	carcinoembryonic antigen related cell adhesion molecule 6	PanCancer Progression
CEP170	centrosomal protein 170	PanCancer Progression
CEP295	centrosomal protein 295	PanCancer Progression
CFP	complement factor properdin	PanCancer Progression
CGN	cingulin	PanCancer Progression
CHAD	chondroadherin	PanCancer Progression

CHD4	chromodomain helicase DNA binding protein 4	PanCancer Progression
CHI3L1	chitinase 3 like 1	PanCancer Progression
CHP1	calcineurin like EF-hand protein 1	PanCancer Progression
CHP2	calcineurin like EF-hand protein 2	PanCancer Progression
CHRDL1	chordin like 1	PanCancer Progression
CHRNA7	cholinergic receptor nicotinic alpha 7 subunit	PanCancer Progression
CIB1	calcium and integrin binding 1	PanCancer Progression
CKMT1A	creatine kinase, mitochondrial 1A	PanCancer Progression
CLDN1	claudin 1	PanCancer Progression
CLDN3	claudin 3	PanCancer Progression
CLDN4	claudin 4	PanCancer Progression
CLDN7	claudin 7	PanCancer Progression
CLEC2B	C-type lectin domain family 2 member B	PanCancer Progression
CLEC3B	C-type lectin domain family 3 member B	PanCancer Progression
CLIC4	chloride intracellular channel 4	PanCancer Progression
CLU	clusterin	PanCancer Progression
CMA1	chymase 1	PanCancer Progression
CNN1	calponin 1	PanCancer Progression
COL18A1	collagen type XVIII alpha 1 chain	PanCancer Progression
COL1A1	collagen type I alpha 1 chain	PanCancer Progression
COL1A2	collagen type I alpha 2 chain	PanCancer Progression
COL3A1	collagen type III alpha 1 chain	PanCancer Progression
COL4A1	collagen type IV alpha 1 chain	PanCancer Progression

COL4A2	collagen type IV alpha 2 chain	PanCancer Progression
COL4A6	collagen type IV alpha 6 chain	PanCancer Progression
COL5A1	collagen type V alpha 1 chain	PanCancer Progression
COL5A2	collagen type V alpha 2 chain	PanCancer Progression
COL6A1	collagen type VI alpha 1 chain	PanCancer Progression
COL6A2	collagen type VI alpha 2 chain	PanCancer Progression
COL6A3	collagen type VI alpha 3 chain	PanCancer Progression
COL7A1	collagen type VII alpha 1 chain	PanCancer Progression
COMP	cartilage oligomeric matrix protein	PanCancer Progression
CREBBP	CREB binding protein	PanCancer Progression
CRIP2	cysteine rich protein 2	PanCancer Progression
CRISPLD2	cysteine rich secretory protein LCCL domain containing 2	PanCancer Progression
CSF2RB	colony stimulating factor 2 receptor beta common subunit	PanCancer Progression
CSPG4	chondroitin sulfate proteoglycan 4	PanCancer Progression
CST7	cystatin F	PanCancer Progression
CTNNB1	catenin beta 1	PanCancer Progression
CTNND1	catenin delta 1	PanCancer Progression
CTSG	cathepsin G	PanCancer Progression
CTSH	cathepsin H	PanCancer Progression
CTSK	cathepsin K	PanCancer Progression
CTSL	cathepsin L	PanCancer Progression
CUL1	cullin 1	PanCancer Progression
CX3CL1	C-X3-C motif chemokine ligand 1	PanCancer Progression

CXADR	CXADR, Ig-like cell adhesion molecule	PanCancer Progression
CXCL10	C-X-C motif chemokine ligand 10	PanCancer Progression
CXCL11	C-X-C motif chemokine ligand 11	PanCancer Progression
CXCL12	C-X-C motif chemokine ligand 12	PanCancer Progression
CXCL13	C-X-C motif chemokine ligand 13	PanCancer Progression
CXCL17	C-X-C motif chemokine ligand 17	PanCancer Progression
CXCL8	C-X-C motif chemokine ligand 8	PanCancer Progression
CXCR2	C-X-C motif chemokine receptor 2	PanCancer Progression
CXCR3	C-X-C motif chemokine receptor 3	PanCancer Progression
CXCR4	C-X-C motif chemokine receptor 4	PanCancer Progression
CYB561	cytochrome b561	PanCancer Progression
CYBB	cytochrome b-245 beta chain	PanCancer Progression
CYP1B1	cytochrome P450 family 1 subfamily B member 1	PanCancer Progression
DAG1	dystroglycan 1	PanCancer Progression
DCC	DCC netrin 1 receptor	PanCancer Progression
DCN	decorin	PanCancer Progression
DDR2	discoidin domain receptor tyrosine kinase 2	PanCancer Progression
DENND5A	DENN domain containing 5A	PanCancer Progression
DENR	density regulated re-initiation and release factor	PanCancer Progression
DESI1	desumoylating isopeptidase 1	PanCancer Progression
DICER1	dicer 1, ribonuclease III	PanCancer Progression
DLC1	DLC1 Rho GTPase activating protein	PanCancer Progression
DLG1	discs large MAGUK scaffold protein 1	PanCancer Progression

DLL4	delta like canonical Notch ligand 4	PanCancer Progression
DPT	dermatopontin	PanCancer Progression
DPYSL3	dihydropyrimidinase like 3	PanCancer Progression
DSC2	desmocollin 2	PanCancer Progression
DST	dystonin	PanCancer Progression
ECM1	extracellular matrix protein 1	PanCancer Progression
ECM2	extracellular matrix protein 2	PanCancer Progression
ECSCR	endothelial cell surface expressed chemotaxis and apoptosis regulator	PanCancer Progression
EDN1	endothelin 1	PanCancer Progression
EGF	epidermal growth factor	PanCancer Progression
EGFL7	EGF like domain multiple 7	PanCancer Progression
EGFR	epidermal growth factor receptor	PanCancer Progression
EGLN2	egl-9 family hypoxia inducible factor 2	PanCancer Progression
EGLN3	egl-9 family hypoxia inducible factor 3	PanCancer Progression
EIF2AK3	eukaryotic translation initiation factor 2 alpha kinase 3	PanCancer Progression
EIF4E2	eukaryotic translation initiation factor 4E family member 2	PanCancer Progression
EIF4EBP1	eukaryotic translation initiation factor 4E binding protein 1	PanCancer Progression
ELF3	E74 like ETS transcription factor 3	PanCancer Progression
ELK3	ELK3, ETS transcription factor	PanCancer Progression
EMCN	endomucin	PanCancer Progression
EMILIN1	elastin microfibril interfacer 1	PanCancer Progression
EMILIN3	elastin microfibril interfacer 3	PanCancer Progression
EMP3	epithelial membrane protein 3	PanCancer Progression

ENO1	enolase 1	PanCancer Progression
ENO2	enolase 2	PanCancer Progression
ENO3	enolase 3	PanCancer Progression
ENPEP	glutamyl aminopeptidase	PanCancer Progression
ENPP2	ectonucleotide pyrophosphatase/phosphodiesterase 2	PanCancer Progression
EP300	E1A binding protein p300	PanCancer Progression
EPAS1	endothelial PAS domain protein 1	PanCancer Progression
EPCAM	epithelial cell adhesion molecule	PanCancer Progression
EPHA1	EPH receptor A1	PanCancer Progression
EPHA2	EPH receptor A2	PanCancer Progression
EPHB1	EPH receptor B1	PanCancer Progression
EPHB3	EPH receptor B3	PanCancer Progression
EPHB4	EPH receptor B4	PanCancer Progression
EPN3	epsin 3	PanCancer Progression
EPS8L1	EPS8 like 1	PanCancer Progression
ERBB2	erb-b2 receptor tyrosine kinase 2	PanCancer Progression
ERBB2IP	erbb2 interacting protein	PanCancer Progression
ERBB3	erb-b2 receptor tyrosine kinase 3	PanCancer Progression
EREG	epiregulin	PanCancer Progression
ERMP1	endoplasmic reticulum metallopeptidase 1	PanCancer Progression
ESRP1	epithelial splicing regulatory protein 1	PanCancer Progression
ETV4	ETS variant 4	PanCancer Progression
EVI2A	ecotropic viral integration site 2A	PanCancer Progression

EVPL	envoplakin	PanCancer Progression
F11R	F11 receptor	PanCancer Progression
F3	coagulation factor III, tissue factor	PanCancer Progression
FAM174B	family with sequence similarity 174 member B	PanCancer Progression
FAP	fibroblast activation protein alpha	PanCancer Progression
FASLG	Fas ligand	PanCancer Progression
FBLN1	fibulin 1	PanCancer Progression
FBLN5	fibulin 5	PanCancer Progression
FBN1	fibrillin 1	PanCancer Progression
FBN2	fibrillin 2	PanCancer Progression
FBP1	fructose-bisphosphatase 1	PanCancer Progression
FERMT2	fermitin family member 2	PanCancer Progression
FGF18	fibroblast growth factor 18	PanCancer Progression
FGF2	fibroblast growth factor 2	PanCancer Progression
FGF9	fibroblast growth factor 9	PanCancer Progression
FGFR1	fibroblast growth factor receptor 1	PanCancer Progression
FGFR2	fibroblast growth factor receptor 2	PanCancer Progression
FGFR3	fibroblast growth factor receptor 3	PanCancer Progression
FGFR4	fibroblast growth factor receptor 4	PanCancer Progression
FGL2	fibrinogen like 2	PanCancer Progression
FHL1	four and a half LIM domains 1	PanCancer Progression
FIGF	vascular endothelial growth factor D	PanCancer Progression
FLI1	Fli-1 proto-oncogene, ETS transcription factor	PanCancer Progression

FLT1	fms related tyrosine kinase 1	PanCancer Progression
FLT4	fms related tyrosine kinase 4	PanCancer Progression
FMOD	fibromodulin	PanCancer Progression
FN1	fibronectin 1	PanCancer Progression
FOXC2	forkhead box C2	PanCancer Progression
FOXO4	forkhead box O4	PanCancer Progression
FRAS1	Fraser extracellular matrix complex subunit 1	PanCancer Progression
FREM1	FRAS1 related extracellular matrix 1	PanCancer Progression
FREM2	FRAS1 related extracellular matrix protein 2	PanCancer Progression
FST	follistatin	PanCancer Progression
FSTL1	follistatin like 1	PanCancer Progression
FUT3	fucosyltransferase 3 (Lewis blood group)	PanCancer Progression
FXYD6	FXYD domain containing ion transport regulator 6	PanCancer Progression
GALNT7	polypeptide N-acetylgalactosaminyltransferase 7	PanCancer Progression
GATA4	GATA binding protein 4	PanCancer Progression
GDF15	growth differentiation factor 15	PanCancer Progression
GDF5	growth differentiation factor 5	PanCancer Progression
GDF6	growth differentiation factor 6	PanCancer Progression
GIMAP4	GTPase, IMAP family member 4	PanCancer Progression
GIMAP6	GTPase, IMAP family member 6	PanCancer Progression
GJA5	gap junction protein alpha 5	PanCancer Progression
GLYR1	glyoxylate reductase 1 homolog	PanCancer Progression
GPI	glucose-6-phosphate isomerase	PanCancer Progression

GPR124	adhesion G protein-coupled receptor A2	PanCancer Progression
GPR56	adhesion G protein-coupled receptor G1	PanCancer Progression
GPX1	glutathione peroxidase 1	PanCancer Progression
GREM1	gremlin 1, DAN family BMP antagonist	PanCancer Progression
GRHL2	grainyhead like transcription factor 2	PanCancer Progression
GSN	gelsolin	PanCancer Progression
GTF2I	general transcription factor IIi	PanCancer Progression
GZMK	granzyme K	PanCancer Progression
HAPLN1	hyaluronan and proteoglycan link protein 1	PanCancer Progression
HAS1	hyaluronan synthase 1	PanCancer Progression
HDAC5	histone deacetylase 5	PanCancer Progression
HDHD3	haloacid dehalogenase like hydrolase domain containing 3	PanCancer Progression
HEG1	heart development protein with EGF like domains 1	PanCancer Progression
HGF	hepatocyte growth factor	PanCancer Progression
HIF1A	hypoxia inducible factor 1 alpha subunit	PanCancer Progression
HIPK1	homeodomain interacting protein kinase 1	PanCancer Progression
HIPK2	homeodomain interacting protein kinase 2	PanCancer Progression
HK2	hexokinase 2	PanCancer Progression
HK3	hexokinase 3	PanCancer Progression
HKDC1	hexokinase domain containing 1	PanCancer Progression
HLA-DPB1	major histocompatibility complex, class II, DP beta 1	PanCancer Progression
HMOX1	heme oxygenase 1	PanCancer Progression
HOXA5	homeobox A5	PanCancer Progression

HOXA7	homeobox A7	PanCancer Progression
HOXB13	homeobox B13	PanCancer Progression
HOXB3	homeobox B3	PanCancer Progression
HPSE	heparanase	PanCancer Progression
HRAS	HRas proto-oncogene, GTPase	PanCancer Progression
HSD17B12	hydroxysteroid 17-beta dehydrogenase 12	PanCancer Progression
HSP90B1	heat shock protein 90 beta family member 1	PanCancer Progression
HSPB1	heat shock protein family B (small) member 1	PanCancer Progression
HSPG2	heparan sulfate proteoglycan 2	PanCancer Progression
HUNK	hormonally up-regulated Neu-associated kinase	PanCancer Progression
IBSP	integrin binding sialoprotein	PanCancer Progression
ICAM1	intercellular adhesion molecule 1	PanCancer Progression
ID1	inhibitor of DNA binding 1, HLH protein	PanCancer Progression
ID2	inhibitor of DNA binding 2	PanCancer Progression
ID4	inhibitor of DNA binding 4, HLH protein	PanCancer Progression
IFNG	interferon gamma	PanCancer Progression
IGF1	insulin like growth factor 1	PanCancer Progression
IGFBP4	insulin like growth factor binding protein 4	PanCancer Progression
IGFBP7	insulin like growth factor binding protein 7	PanCancer Progression
IL10RA	interleukin 10 receptor subunit alpha	PanCancer Progression
IL11	interleukin 11	PanCancer Progression
IL13RA2	interleukin 13 receptor subunit alpha 2	PanCancer Progression
IL15	interleukin 15	PanCancer Progression

IL18	interleukin 18	PanCancer Progression
IL1A	interleukin 1 alpha	PanCancer Progression
IL1B	interleukin 1 beta	PanCancer Progression
IL1RL1	interleukin 1 receptor like 1	PanCancer Progression
IL1RN	interleukin 1 receptor antagonist	PanCancer Progression
IL6	interleukin 6	PanCancer Progression
ILK	integrin linked kinase	PanCancer Progression
INHBA	inhibin beta A subunit	PanCancer Progression
INHBE	inhibin beta E subunit	PanCancer Progression
IRF6	interferon regulatory factor 6	PanCancer Progression
ISL1	ISL LIM homeobox 1	PanCancer Progression
ISLR	immunoglobulin superfamily containing leucine rich repeat	PanCancer Progression
ITGA1	integrin subunit alpha 1	PanCancer Progression
ITGA11	integrin subunit alpha 11	PanCancer Progression
ITGA2	integrin subunit alpha 2	PanCancer Progression
ITGA3	integrin subunit alpha 3	PanCancer Progression
ITGA5	integrin subunit alpha 5	PanCancer Progression
ITGA6	integrin subunit alpha 6	PanCancer Progression
ITGA7	integrin subunit alpha 7	PanCancer Progression
ITGA8	integrin subunit alpha 8	PanCancer Progression
ITGA9	integrin subunit alpha 9	PanCancer Progression
ITGAM	integrin subunit alpha M	PanCancer Progression
ITGB1	integrin subunit beta 1	PanCancer Progression

ITGB1BP1	integrin subunit beta 1 binding protein 1	PanCancer Progression
ITGB2	integrin subunit beta 2	PanCancer Progression
ITGB3	integrin subunit beta 3	PanCancer Progression
ITGB4	integrin subunit beta 4	PanCancer Progression
ITGB6	integrin subunit beta 6	PanCancer Progression
ITGB7	integrin subunit beta 7	PanCancer Progression
ITGB8	integrin subunit beta 8	PanCancer Progression
ITM2A	integral membrane protein 2A	PanCancer Progression
JAG1	jagged 1	PanCancer Progression
JAM2	junctional adhesion molecule 2	PanCancer Progression
JAM3	junctional adhesion molecule 3	PanCancer Progression
JUN	Jun proto-oncogene, AP-1 transcription factor subunit	PanCancer Progression
KCNJ8	potassium voltage-gated channel subfamily J member 8	PanCancer Progression
KDM1A	lysine demethylase 1A	PanCancer Progression
KDR	kinase insert domain receptor	PanCancer Progression
KIAA1462	junctional cadherin 5 associated	PanCancer Progression
KISS1	KiSS-1 metastasis suppressor	PanCancer Progression
KLK3	kallikrein related peptidase 3	PanCancer Progression
KRAS	KRAS proto-oncogene, GTPase	PanCancer Progression
KRIT1	KRIT1, ankyrin repeat containing	PanCancer Progression
KRT1	keratin 1	PanCancer Progression
KRT14	keratin 14	PanCancer Progression
KRT19	keratin 19	PanCancer Progression

KRT7	keratin 7	PanCancer Progression
LAD1	ladinin 1	PanCancer Progression
LAMA1	laminin subunit alpha 1	PanCancer Progression
LAMA3	laminin subunit alpha 3	PanCancer Progression
LAMA4	laminin subunit alpha 4	PanCancer Progression
LAMA5	laminin subunit alpha 5	PanCancer Progression
LAMB3	laminin subunit beta 3	PanCancer Progression
LAMC1	laminin subunit gamma 1	PanCancer Progression
LAMC2	laminin subunit gamma 2	PanCancer Progression
LDHA	lactate dehydrogenase A	PanCancer Progression
LEFTY1	left-right determination factor 1	PanCancer Progression
LGALS1	galectin 1	PanCancer Progression
LHFP	LHFPL tetraspan subfamily member 6	PanCancer Progression
LIFR	LIF receptor alpha	PanCancer Progression
LLGL2	LLGL2, scribble cell polarity complex component	PanCancer Progression
LOX	lysyl oxidase	PanCancer Progression
LOXL2	lysyl oxidase like 2	PanCancer Progression
LRG1	leucine rich alpha-2-glycoprotein 1	PanCancer Progression
LTBP4	latent transforming growth factor beta binding protein 4	PanCancer Progression
LUM	lumican	PanCancer Progression
LY96	lymphocyte antigen 96	PanCancer Progression
MAF	MAF bZIP transcription factor	PanCancer Progression
MAP2K1	mitogen-activated protein kinase kinase 1	PanCancer Progression

MAP2K2	mitogen-activated protein kinase kinase 2	PanCancer Progression
MAP2K4	mitogen-activated protein kinase kinase 4	PanCancer Progression
MAP3K7	mitogen-activated protein kinase kinase kinase 7	PanCancer Progression
MAPK1	mitogen-activated protein kinase 1	PanCancer Progression
MAPK3	mitogen-activated protein kinase 3	PanCancer Progression
MAPKAPK 3	mitogen-activated protein kinase-activated protein kinase 3	PanCancer Progression
MCAM	melanoma cell adhesion molecule	PanCancer Progression
MED1	mediator complex subunit 1	PanCancer Progression
MED23	mediator complex subunit 23	PanCancer Progression
MEG3	maternally expressed 3 (non-protein coding)	PanCancer Progression
MEOX2	mesenchyme homeobox 2	PanCancer Progression
MET	MET proto-oncogene, receptor tyrosine kinase	PanCancer Progression
MFAP4	microfibril associated protein 4	PanCancer Progression
MGAT5	mannosyl (alpha-1,6-)-glycoprotein beta-1,6-N-acetyl-glucosaminyltransferase	PanCancer Progression
MGP	matrix Gla protein	PanCancer Progression
MISP	mitotic spindle positioning	PanCancer Progression
MMP1	matrix metallopeptidase 1	PanCancer Progression
MMP10	matrix metallopeptidase 10	PanCancer Progression
MMP12	matrix metallopeptidase 12	PanCancer Progression
MMP13	matrix metallopeptidase 13	PanCancer Progression
MMP14	matrix metallopeptidase 14	PanCancer Progression
MMP17	matrix metallopeptidase 17	PanCancer Progression
MMP2	matrix metallopeptidase 2	PanCancer Progression

MMP24	matrix metallopeptidase 24	PanCancer Progression
MMP3	matrix metallopeptidase 3	PanCancer Progression
MMP9	matrix metallopeptidase 9	PanCancer Progression
MMRN2	multimerin 2	PanCancer Progression
MPDZ	multiple PDZ domain crumbs cell polarity complex component	PanCancer Progression
MRC1	mannose receptor C-type 1	PanCancer Progression
MS4A4A	membrane spanning 4-domains A4A	PanCancer Progression
MS4A6A	membrane spanning 4-domains A6A	PanCancer Progression
MT3	metallothionein 3	PanCancer Progression
MTA1	metastasis associated 1	PanCancer Progression
MTBP	MDM2 binding protein	PanCancer Progression
MTDH	metadherin	PanCancer Progression
MTOR	mechanistic target of rapamycin kinase	PanCancer Progression
MUC1	mucin 1, cell surface associated	PanCancer Progression
MYC	MYC proto-oncogene, bHLH transcription factor	PanCancer Progression
MYCL	MYCL proto-oncogene, bHLH transcription factor	PanCancer Progression
MYH11	myosin heavy chain 11	PanCancer Progression
MYLK	myosin light chain kinase	PanCancer Progression
MYO1D	myosin ID	PanCancer Progression
MYO5C	myosin VC	PanCancer Progression
NAA15	N(alpha)-acetyltransferase 15, NatA auxiliary subunit	PanCancer Progression
NAP1L3	nucleosome assembly protein 1 like 3	PanCancer Progression
NCAM1	neural cell adhesion molecule 1	PanCancer Progression

NCL	nucleolin	PanCancer Progression
NDNF	neuron derived neurotrophic factor	PanCancer Progression
NDP	NDP, norrin cystine knot growth factor	PanCancer Progression
NDRG1	N-myc downstream regulated 1	PanCancer Progression
NF1	neurofibromin 1	PanCancer Progression
NF2	neurofibromin 2	PanCancer Progression
NFAT5	nuclear factor of activated T cells 5	PanCancer Progression
NFATC2	nuclear factor of activated T cells 2	PanCancer Progression
NFKB1	nuclear factor kappa B subunit 1	PanCancer Progression
NID2	nidogen 2	PanCancer Progression
NME1	NME/NM23 nucleoside diphosphate kinase 1	PanCancer Progression
NME4	NME/NM23 nucleoside diphosphate kinase 4	PanCancer Progression
NODAL	nodal growth differentiation factor	PanCancer Progression
NOS2	nitric oxide synthase 2	PanCancer Progression
NOS3	nitric oxide synthase 3	PanCancer Progression
NOTCH1	notch 1	PanCancer Progression
NOX5	NADPH oxidase 5	PanCancer Progression
NPR1	natriuretic peptide receptor 1	PanCancer Progression
NR3C1	nuclear receptor subfamily 3 group C member 1	PanCancer Progression
NR4A1	nuclear receptor subfamily 4 group A member 1	PanCancer Progression
NR4A3	nuclear receptor subfamily 4 group A member 3	PanCancer Progression
NRCAM	neuronal cell adhesion molecule	PanCancer Progression
NRP1	neuropilin 1	PanCancer Progression

NRP2	neuropilin 2	PanCancer Progression
NRXN1	neurexin 1	PanCancer Progression
NRXN3	neurexin 3	PanCancer Progression
NTRK1	neurotrophic receptor tyrosine kinase 1	PanCancer Progression
OAS1	2'-5'-oligoadenylate synthetase 1	PanCancer Progression
OCLN	occludin	PanCancer Progression
OGN	osteoglycin	PanCancer Progression
OLFML2B	olfactomedin like 2B	PanCancer Progression
OVOL2	ovo like zinc finger 2	PanCancer Progression
P3H1	prolyl 3-hydroxylase 1	PanCancer Progression
P3H2	prolyl 3-hydroxylase 2	PanCancer Progression
PCOLCE	procollagen C-endopeptidase enhancer	PanCancer Progression
PDCD10	programmed cell death 10	PanCancer Progression
PDCL3	phosducin like 3	PanCancer Progression
PDGFA	platelet derived growth factor subunit A	PanCancer Progression
PDGFC	platelet derived growth factor C	PanCancer Progression
PDGFRB	platelet derived growth factor receptor beta	PanCancer Progression
PDK1	pyruvate dehydrogenase kinase 1	PanCancer Progression
PDPN	podoplanin	PanCancer Progression
PEBP4	phosphatidylethanolamine binding protein 4	PanCancer Progression
PECAM1	platelet and endothelial cell adhesion molecule 1	PanCancer Progression
PFKFB1	6-phosphofructo-2-kinase/fructose-2,6-biphosphatase 1	PanCancer Progression
PFKFB4	6-phosphofructo-2-kinase/fructose-2,6-biphosphatase 4	PanCancer Progression

PGK1	phosphoglycerate kinase 1	PanCancer Progression
PIK3CA	phosphatidylinositol-4,5-bisphosphate 3-kinase catalytic subunit alpha	PanCancer Progression
PIK3CD	phosphatidylinositol-4,5-bisphosphate 3-kinase catalytic subunit delta	PanCancer Progression
PIK3CG	phosphatidylinositol-4,5-bisphosphate 3-kinase catalytic subunit gamma	PanCancer Progression
PIK3R1	phosphoinositide-3-kinase regulatory subunit 1	PanCancer Progression
PIK3R2	phosphoinositide-3-kinase regulatory subunit 2	PanCancer Progression
PIK3R5	phosphoinositide-3-kinase regulatory subunit 5	PanCancer Progression
PIK3R6	phosphoinositide-3-kinase regulatory subunit 6	PanCancer Progression
PITX2	paired like homeodomain 2	PanCancer Progression
PKM	pyruvate kinase M1/2	PanCancer Progression
PKN1	protein kinase N1	PanCancer Progression
PKNOX1	PBX/knotted 1 homeobox 1	PanCancer Progression
PLA2G10	phospholipase A2 group X	PanCancer Progression
PLA2G2A	phospholipase A2 group IIA	PanCancer Progression
PLA2G2D	phospholipase A2 group IID	PanCancer Progression
PLA2G3	phospholipase A2 group III	PanCancer Progression
PLAU	plasminogen activator, urokinase	PanCancer Progression
PLAUR	plasminogen activator, urokinase receptor	PanCancer Progression
PLCG1	phospholipase C gamma 1	PanCancer Progression
PLCG2	phospholipase C gamma 2	PanCancer Progression
PLEKHO1	pleckstrin homology domain containing O1	PanCancer Progression
PLS1	plastin 1	PanCancer Progression
PLXDC1	plexin domain containing 1	PanCancer Progression

PLXNC1	plexin C1	PanCancer Progression
PLXND1	plexin D1	PanCancer Progression
PMP22	peripheral myelin protein 22	PanCancer Progression
PNPLA6	patatin like phospholipase domain containing 6	PanCancer Progression
POPDC3	popeye domain containing 3	PanCancer Progression
POSTN	periostin	PanCancer Progression
PPFIBP2	PPFIA binding protein 2	PanCancer Progression
PPL	periplakin	PanCancer Progression
PPP1R16B	protein phosphatase 1 regulatory subunit 16B	PanCancer Progression
PPP2CB	protein phosphatase 2 catalytic subunit beta	PanCancer Progression
PPP2R1A	protein phosphatase 2 scaffold subunit Aalpha	PanCancer Progression
PPP3R1	protein phosphatase 3 regulatory subunit B, alpha	PanCancer Progression
PRELP	proline and arginine rich end leucine rich repeat protein	PanCancer Progression
PRF1	perforin 1	PanCancer Progression
PRKCB	protein kinase C beta	PanCancer Progression
PRKCG	protein kinase C gamma	PanCancer Progression
PRKCZ	protein kinase C zeta	PanCancer Progression
PROK2	prokineticin 2	PanCancer Progression
PROM1	prominin 1	PanCancer Progression
PRR15L	proline rich 15 like	PanCancer Progression
PRSS22	protease, serine 22	PanCancer Progression
PRSS8	protease, serine 8	PanCancer Progression
PTEN	phosphatase and tensin homolog	PanCancer Progression

PTGDS	prostaglandin D2 synthase	PanCancer Progression
PTGIS	prostaglandin I2 synthase	PanCancer Progression
PTGS2	prostaglandin-endoperoxide synthase 2	PanCancer Progression
PTK2	protein tyrosine kinase 2	PanCancer Progression
PTK2B	protein tyrosine kinase 2 beta	PanCancer Progression
PTK6	protein tyrosine kinase 6	PanCancer Progression
PTPRB	protein tyrosine phosphatase, receptor type B	PanCancer Progression
PTPRC	protein tyrosine phosphatase, receptor type C	PanCancer Progression
PTPRM	protein tyrosine phosphatase, receptor type M	PanCancer Progression
PTRF	caveolae associated protein 1	PanCancer Progression
PTTG1	pituitary tumor-transforming 1	PanCancer Progression
PTX3	pentraxin 3	PanCancer Progression
PXDN	peroxidasin	PanCancer Progression
PYCARD	PYD and CARD domain containing	PanCancer Progression
QKI	QKI, KH domain containing RNA binding	PanCancer Progression
RAB25	RAB25, member RAS oncogene family	PanCancer Progression
RAC1	Rac family small GTPase 1	PanCancer Progression
RAC2	Rac family small GTPase 2	PanCancer Progression
RAF1	Raf-1 proto-oncogene, serine/threonine kinase	PanCancer Progression
RAMP1	receptor activity modifying protein 1	PanCancer Progression
RAMP2	receptor activity modifying protein 2	PanCancer Progression
RB1	RB transcriptional corepressor 1	PanCancer Progression
RBL1	RB transcriptional corepressor like 1	PanCancer Progression

RBL2	RB transcriptional corepressor like 2	PanCancer Progression
RBM47	RNA binding motif protein 47	PanCancer Progression
RBPJ	recombination signal binding protein for immunoglobulin kappa J region	PanCancer Progression
RBX1	ring-box 1	PanCancer Progression
RELN	reelin	PanCancer Progression
RGCC	regulator of cell cycle	PanCancer Progression
RHOA	ras homolog family member A	PanCancer Progression
RNH1	ribonuclease/angiogenin inhibitor 1	PanCancer Progression
ROBO4	roundabout guidance receptor 4	PanCancer Progression
ROCK1	Rho associated coiled-coil containing protein kinase 1	PanCancer Progression
ROCK2	Rho associated coiled-coil containing protein kinase 2	PanCancer Progression
RORA	RAR related orphan receptor A	PanCancer Progression
RORB	RAR related orphan receptor B	PanCancer Progression
RPS27A	ribosomal protein S27a	PanCancer Progression
RPS6KB1	ribosomal protein S6 kinase B1	PanCancer Progression
RPS6KB2	ribosomal protein S6 kinase B2	PanCancer Progression
RRAS	RAS related	PanCancer Progression
RTN4	reticulon 4	PanCancer Progression
RUNX1	runt related transcription factor 1	PanCancer Progression
RUNX1T1	RUNX1 translocation partner 1	PanCancer Progression
S100A14	S100 calcium binding protein A14	PanCancer Progression
S100A7	S100 calcium binding protein A7	PanCancer Progression
S1PR1	sphingosine-1-phosphate receptor 1	PanCancer Progression

SACS	sacsin molecular chaperone	PanCancer Progression
SAMSN1	SAM domain, SH3 domain and nuclear localization signals 1	PanCancer Progression
SCG2	secretogranin II	PanCancer Progression
SCNN1A	sodium channel epithelial 1 alpha subunit	PanCancer Progression
SDC4	syndecan 4	PanCancer Progression
SELE	selectin E	PanCancer Progression
SEMA3E	semaphorin 3E	PanCancer Progression
SERINC5	serine incorporator 5	PanCancer Progression
SERPINA1	serpin family A member 1	PanCancer Progression
SERPINE1	serpin family E member 1	PanCancer Progression
SERPINF1	serpin family F member 1	PanCancer Progression
SERPING1	serpin family G member 1	PanCancer Progression
SERPINH1	serpin family H member 1	PanCancer Progression
SET	SET nuclear proto-oncogene	PanCancer Progression
SETD2	SET domain containing 2	PanCancer Progression
SFRP1	secreted frizzled related protein 1	PanCancer Progression
SFRP2	secreted frizzled related protein 2	PanCancer Progression
SH2B3	SH2B adaptor protein 3	PanCancer Progression
SH2D3A	SH2 domain containing 3A	PanCancer Progression
SH3YL1	SH3 and SYLF domain containing 1	PanCancer Progression
SHB	SH2 domain containing adaptor protein B	PanCancer Progression
SIRT1	sirtuin 1	PanCancer Progression
SKP1	S-phase kinase associated protein 1	PanCancer Progression

SLC12A6	solute carrier family 12 member 6	PanCancer Progression
SLC2A1	solute carrier family 2 member 1	PanCancer Progression
SLC35A3	solute carrier family 35 member A3	PanCancer Progression
SLC37A1	solute carrier family 37 member 1	PanCancer Progression
SLC44A4	solute carrier family 44 member 4	PanCancer Progression
SLIT2	slit guidance ligand 2	PanCancer Progression
SLPI	secretory leukocyte peptidase inhibitor	PanCancer Progression
SMAD1	SMAD family member 1	PanCancer Progression
SMAD2	SMAD family member 2	PanCancer Progression
SMAD3	SMAD family member 3	PanCancer Progression
SMAD4	SMAD family member 4	PanCancer Progression
SMAD5	SMAD family member 5	PanCancer Progression
SMAD9	SMAD family member 9	PanCancer Progression
SMC3	structural maintenance of chromosomes 3	PanCancer Progression
SMOC1	SPARC related modular calcium binding 1	PanCancer Progression
SMURF1	SMAD specific E3 ubiquitin protein ligase 1	PanCancer Progression
SMURF2	SMAD specific E3 ubiquitin protein ligase 2	PanCancer Progression
SNAI1	snail family transcriptional repressor 1	PanCancer Progression
SNAI2	snail family transcriptional repressor 2	PanCancer Progression
SNAI3	snail family transcriptional repressor 3	PanCancer Progression
SNRPF	small nuclear ribonucleoprotein polypeptide F	PanCancer Progression
SOD1	superoxide dismutase 1	PanCancer Progression
SORD	sorbitol dehydrogenase	PanCancer Progression

SOX17	SRY-box 17	PanCancer Progression
SOX2	SRY-box 2	PanCancer Progression
SOX9	SRY-box 9	PanCancer Progression
SP1	Sp1 transcription factor	PanCancer Progression
SPARC	secreted protein acidic and cysteine rich	PanCancer Progression
SPARCL1	SPARC like 1	PanCancer Progression
SPDEF	SAM pointed domain containing ETS transcription factor	PanCancer Progression
SPHK2	sphingosine kinase 2	PanCancer Progression
SPINK5	serine peptidase inhibitor, Kazal type 5	PanCancer Progression
SPINT1	serine peptidase inhibitor, Kunitz type 1	PanCancer Progression
SPOCK3	SPARC/osteonectin, cwcv and kazal like domains proteoglycan 3	PanCancer Progression
SPP1	secreted phosphoprotein 1	PanCancer Progression
SRC	SRC proto-oncogene, non-receptor tyrosine kinase	PanCancer Progression
SRF	serum response factor	PanCancer Progression
SRGN	serglycin	PanCancer Progression
SRPK2	SRSF protein kinase 2	PanCancer Progression
SRPX2	sushi repeat containing protein, X-linked 2	PanCancer Progression
SSTR2	somatostatin receptor 2	PanCancer Progression
ST14	suppression of tumorigenicity 14	PanCancer Progression
STAB1	stabilin 1	PanCancer Progression
STAB2	stabilin 2	PanCancer Progression
STAT1	signal transducer and activator of transcription 1	PanCancer Progression
STAT3	signal transducer and activator of transcription 3	PanCancer Progression

SULF1	sulfatase 1	PanCancer Progression
SV2B	synaptic vesicle glycoprotein 2B	PanCancer Progression
SYK	spleen associated tyrosine kinase	PanCancer Progression
SYNE1	spectrin repeat containing nuclear envelope protein 1	PanCancer Progression
TACSTD2	tumor associated calcium signal transducer 2	PanCancer Progression
TAL1	TAL bHLH transcription factor 1, erythroid differentiation factor	PanCancer Progression
TBX1	T-box 1	PanCancer Progression
TBX4	T-box 4	PanCancer Progression
TBXA2R	thromboxane A2 receptor	PanCancer Progression
TCEB1	elongin C	PanCancer Progression
TCEB2	elongin B	PanCancer Progression
TCF20	transcription factor 20	PanCancer Progression
TCF3	transcription factor 3	PanCancer Progression
TCF4	transcription factor 4	PanCancer Progression
TDGF1	teratocarcinoma-derived growth factor 1	PanCancer Progression
TEK	TEK receptor tyrosine kinase	PanCancer Progression
TF	transferrin	PanCancer Progression
TFDP1	transcription factor Dp-1	PanCancer Progression
TFPI2	tissue factor pathway inhibitor 2	PanCancer Progression
TGFB1	transforming growth factor beta 1	PanCancer Progression
TGFB2	transforming growth factor beta 2	PanCancer Progression
TGFBI	transforming growth factor beta induced	PanCancer Progression
TGFBR2	transforming growth factor beta receptor 2	PanCancer Progression

THBS1	thrombospondin 1	PanCancer Progression
THBS2	thrombospondin 2	PanCancer Progression
THBS4	thrombospondin 4	PanCancer Progression
THY1	Thy-1 cell surface antigen	PanCancer Progression
TIE1	tyrosine kinase with immunoglobulin like and EGF like domains 1	PanCancer Progression
TIMP1	TIMP metallopeptidase inhibitor 1	PanCancer Progression
TIMP2	TIMP metallopeptidase inhibitor 2	PanCancer Progression
TIMP4	TIMP metallopeptidase inhibitor 4	PanCancer Progression
TJP2	tight junction protein 2	PanCancer Progression
TJP3	tight junction protein 3	PanCancer Progression
TLR4	toll like receptor 4	PanCancer Progression
TMC6	transmembrane channel like 6	PanCancer Progression
TMEM100	transmembrane protein 100	PanCancer Progression
TMEM30B	transmembrane protein 30B	PanCancer Progression
TMPRSS2	transmembrane protease, serine 2	PanCancer Progression
TMPRSS4	transmembrane protease, serine 4	PanCancer Progression
TMPRSS6	transmembrane protease, serine 6	PanCancer Progression
TNC	tenascin C	PanCancer Progression
TNF	tumor necrosis factor	PanCancer Progression
TNFRSF12A	TNF receptor superfamily member 12A	PanCancer Progression
TNFRSF1A	TNF receptor superfamily member 1A	PanCancer Progression
TNFSF10	TNF superfamily member 10	PanCancer Progression
TNFSF12	TNF superfamily member 12	PanCancer Progression

TNFSF13	TNF superfamily member 13	PanCancer Progression
TNMD	tenomodulin	PanCancer Progression
TNN	tenascin N	PanCancer Progression
TNS1	tensin 1	PanCancer Progression
TNXB	tenascin XB	PanCancer Progression
TOM1L1	target of myb1 like 1 membrane trafficking protein	PanCancer Progression
TP53	tumor protein p53	PanCancer Progression
TPM2	tropomyosin 2	PanCancer Progression
TPSB2	tryptase beta 2 (gene/pseudogene)	PanCancer Progression
TPSD1	tryptase delta 1	PanCancer Progression
TSHR	thyroid stimulating hormone receptor	PanCancer Progression
TSPAN1	tetraspanin 1	PanCancer Progression
TWIST1	twist family bHLH transcription factor 1	PanCancer Progression
TWIST2	twist family bHLH transcription factor 2	PanCancer Progression
TXNIP	thioredoxin interacting protein	PanCancer Progression
TYMP	thymidine phosphorylase	PanCancer Progression
UBA52	ubiquitin A-52 residue ribosomal protein fusion product 1	PanCancer Progression
UTS2	urotensin 2	PanCancer Progression
VAMP8	vesicle associated membrane protein 8	PanCancer Progression
VASH1	vasohibin 1	PanCancer Progression
VAV2	vav guanine nucleotide exchange factor 2	PanCancer Progression
VAV3	vav guanine nucleotide exchange factor 3	PanCancer Progression
VCAM1	vascular cell adhesion molecule 1	PanCancer Progression

VCAN	versican	PanCancer Progression
VEGFA	vascular endothelial growth factor A	PanCancer Progression
VEGFB	vascular endothelial growth factor B	PanCancer Progression
VEGFC	vascular endothelial growth factor C	PanCancer Progression
VEZF1	vascular endothelial zinc finger 1	PanCancer Progression
VHL	von Hippel-Lindau tumor suppressor	PanCancer Progression
VIM	vimentin	PanCancer Progression
VIT	vitrin	PanCancer Progression
VPS13A	vacuolar protein sorting 13 homolog A	PanCancer Progression
VSIG4	V-set and immunoglobulin domain containing 4	PanCancer Progression
VWA1	von Willebrand factor A domain containing 1	PanCancer Progression
VWA2	von Willebrand factor A domain containing 2	PanCancer Progression
WARS	tryptophanyl-tRNA synthetase	PanCancer Progression
WIPF1	WAS/WASL interacting protein family member 1	PanCancer Progression
WNT5A	Wnt family member 5A	PanCancer Progression
WNT5B	Wnt family member 5B	PanCancer Progression
WWTR1	WW domain containing transcription regulator 1	PanCancer Progression
ZC3H12A	zinc finger CCCH-type containing 12A	PanCancer Progression
ZCCHC24	zinc finger CCHC-type containing 24	PanCancer Progression
ZEB1	zinc finger E-box binding homeobox 1	PanCancer Progression
ZEB2	zinc finger E-box binding homeobox 2	PanCancer Progression
ZFPM2	zinc finger protein, FOG family member 2	PanCancer Progression
ZFYVE16	zinc finger FYVE-type containing 16	PanCancer Progression

ZFYVE9	zinc finger FYVE-type containing 9	PanCancer Progression
AGK	acylglycerol kinase	PanCancer Progression
AMMECR1L	AMMECR1 like	PanCancer Progression
CC2D1B	coiled-coil and C2 domain containing 1B	PanCancer Progression
CNOT10	CCR4-NOT transcription complex subunit 10	PanCancer Progression
CNOT4	CCR4-NOT transcription complex subunit 4	PanCancer Progression
COG7	component of oligomeric golgi complex 7	PanCancer Progression
DDX50	DExD-box helicase 50	PanCancer Progression
DHX16	DEAH-box helicase 16	PanCancer Progression
DNAJC14	DnaJ heat shock protein family (Hsp40) member C14	PanCancer Progression
EDC3	enhancer of mRNA decapping 3	PanCancer Progression
EIF2B4	eukaryotic translation initiation factor 2B subunit delta	PanCancer Progression
ERCC3	ERCC excision repair 3, TFIIH core complex helicase subunit	PanCancer Progression
FCF1	FCF1, rRNA-processing protein	PanCancer Progression
GPATCH3	G-patch domain containing 3	PanCancer Progression
HDAC3	histone deacetylase 3	PanCancer Progression
MRPS5	mitochondrial ribosomal protein S5	PanCancer Progression
MTMR14	myotubularin related protein 14	PanCancer Progression
NOL7	nucleolar protein 7	PanCancer Progression
NUBP1	nucleotide binding protein 1	PanCancer Progression
PRPF38A	pre-mRNA processing factor 38A	PanCancer Progression
SAP130	Sin3A associated protein 130	PanCancer Progression
SF3A3	splicing factor 3a subunit 3	PanCancer Progression

TLK2	tousled like kinase 2	PanCancer Progression
TMUB2	transmembrane and ubiquitin like domain containing 2	PanCancer Progression
TRIM39	tripartite motif containing 39	PanCancer Progression
USP39	ubiquitin specific peptidase 39	PanCancer Progression
ZC3H14	zinc finger CCCH-type containing 14	PanCancer Progression
ZKSCAN5	zinc finger with KRAB and SCAN domains 5	PanCancer Progression
ZNF143	zinc finger protein 143	PanCancer Progression
ZNF346	zinc finger protein 346	PanCancer Progression
ACACA	acetyl-Coenzyme A carboxylase alpha	Custom Content
ALOX5AP	arachidonate 5-lipoxygenase-activating protein	Custom Content
AQP9	aquaporin 9	Custom Content
ART1	ADP-ribosyltransferase 1	Custom Content
BCAT1	branched chain amino-acid transaminase 1, cytosolic	Custom Content
CD300A	CD300a molecule	Custom Content
CERS6	ceramide synthase 6	Custom Content
CPT1A	carnitine palmitoyltransferase 1A (liver)	Custom Content
CTNNBIP1	catenin, beta interacting protein 1	Custom Content
EIF2AK4	eukaryotic translation initiation factor 2 alpha kinase 4	Custom Content
FAM129A	family with sequence similarity 129, member A	Custom Content
FOXO3	forkhead box O3	Custom Content
FOXP1	forkhead box P1	Custom Content
HK1	hexokinase 1	Custom Content
HMGB3	high mobility group box 3	Custom Content
HMGCR	3-hydroxy-3-methylglutaryl-Coenzyme A reductase	Custom Content
HNRNPLL	heterogeneous nuclear ribonucleoprotein L-like	Custom Content
MTHFD1	methylenetetrahydrofolate dehydrogenase, cyclohydrolase and formyltetrahydrofolate synthetase 1	Custom Content
P2RX7	purinergic receptor P2X, ligand-gated ion channel, 7	Custom Content
PDE3B	phosphodiesterase 3B, cGMP-inhibited	Custom Content
PNPLA2	patatin-like phospholipase domain containing 2	Custom Content
PRR5L	proline rich 5 like	Custom Content
SLC3A2	solute carrier family 3 (activators of dibasic and neutral amino acid transport), member 2	Custom Content
SREBF1	sterol regulatory element binding transcription factor 1	Custom Content
SREBF2	sterol regulatory element binding transcription factor 2	Custom Content
TDH	L-threonine dehydrogenase	Custom Content

TIMD4	T-cell immunoglobulin and mucin domain containing 4	Custom Content
TOX	thymus high mobility group box protein TOX	Custom Content
TPP2	tripeptidyl peptidase 2	Custom Content
WNT3A	wingless-type MMTV integration site family, member 3A	Custom Content
ASS1	argininosuccinate synthetase 1	Custom Content
CEACAM3	carcinoembryonic antigen-related cell adhesion molecule 3	Custom Content
CPA3	carboxypeptidase A3	Custom Content
ERCC5	excision repair cross-complementing rodent repair deficiency, complementation group 5	Custom Content
EXO1	exonuclease 1	Custom Content
FCAR	Fc fragment of IgA, receptor for	Custom Content
FCGR3B	Fc fragment of IgG, low affinity IIIb, receptor (CD16b)	Custom Content
FCRL2	Fc receptor-like 2	Custom Content
FPR1	formyl peptide receptor 1	Custom Content
GLS	glutaminase	Custom Content
GLUL	glutamate-ammonia ligase	Custom Content
HAT1	histone acetyltransferase 1	Custom Content
KAT5	K(lysine) acetyltransferase 5	Custom Content
FAM30A	family with sequence similarity 30 member A	Custom Content
KLF2	Kruppel-like factor 2	Custom Content
LIPA	lipase A, lysosomal acid, cholesterol esterase	Custom Content
MLANA	melan-A	Custom Content
MRE11	MRE11 homolog, double strand break repair nuclease	Custom Content
NEIL2	nei like DNA glycosylase 2	Custom Content
PHACTR2	phosphatase and actin regulator 2	Custom Content
PNOC	prepronociceptin	Custom Content
PTGER4	prostaglandin E receptor 4 (subtype EP4)	Custom Content
RPA1	replication protein A1	Custom Content
SEMA4A	sema domain, immunoglobulin domain (Ig), transmembrane domain (TM) and short cytoplasmic domain, (semaphorin) 4A	Custom Content
SIGLEC5	sialic acid binding Ig-like lectin 5	Custom Content
SPIB	Spi-B transcription factor (Spi-1/PU.1 related)	Custom Content
TCL1A	T-cell leukemia/lymphoma 1A	Custom Content
TFAP4	transcription factor AP-4 (activating enhancer binding protein 4)	Custom Content
TOP3A	topoisomerase (DNA) III alpha	Custom Content
TREX1	three prime repair exonuclease 1	Custom Content
VSIR	V-set immunoregulatory receptor	Custom Content
CD69	CD69 molecule	Custom Content
FOXO1	forkhead box O1	Custom Content
GAPDH	glyceraldehyde-3-phosphate dehydrogenase	Custom Content
HDC	histidine decarboxylase	Custom Content
HLA-DRB1	major histocompatibility complex, class II, DR beta 1	Custom Content

IHH	Indian hedgehog	Custom Content
IL4I1	interleukin 4 induced 1	Custom Content
KDR	kinase insert domain receptor	Custom Content
LEXM	chromosome 1 open reading frame 177	Custom Content
MKI67	antigen identified by monoclonal antibody	Custom Content
NKG7	natural killer cell group 7 sequence	Custom Content
NRDE2	chromosome 14 open reading frame 102	Custom Content
OAZ1	ornithine decarboxylase antizyme 1	Custom Content
PF4	platelet factor 4	Custom Content
PGK1	phosphoglycerate kinase 1	Custom Content
PROK2	prokineticin 2	Custom Content
PROM1	prominin 1	Custom Content
REN	renin	Custom Content
RPLP0	ribosomal protein, large, P0	Custom Content
SLC1A5	solute carrier family 1 (neutral amino acid transporter), member 5	Custom Content
SLC7A5	solute carrier family 7 member 5	Custom Content
SP2	Sp2 transcription factor	Custom Content
SPI1	spleen focus forming virus (SFFV) proviral integration oncogene spi1	Custom Content
STK11IP	serine/threonine kinase 11 interacting protein	Custom Content
TBC1D10B	TBC1 domain family, member 10B	Custom Content
TRAT1	T cell receptor associated transmembrane adaptor 1	Custom Content
TSLP	thymic stromal lymphopoietin	Custom Content
UBB	ubiquitin B	Custom Content
XCL1	chemokine (C motif) ligand 1	Custom Content
