# Supplementary material for: CTLA-4 blockade and interferon-α induce proinflammatory transcriptional changes in the tumor immune landscape that correlate with pathologic response in melanoma
Source: PLoS One. 2021 Jan 11;16(1):e0245287. doi: 10.1371/journal.pone.0245287 (PMC7799833; doi:10.1371/journal.pone.0245287)
Supplement: S2 Table — (PDF) [file pone.0245287.s007.pdf]

S2 Table. Patient Demographics and Baseline Disease Characteristics of patients enrolled in clinical trial (N=30 patients)*

**Variable**		**No. of Patients (%)**
Age (years); Median (range)		61 (37-76)
Cutaneous primary		21 (70)
Unknown primary		8 (27)
Mucosal		1 (3)
Gender	Female	12 (40)
	Male	18 (60)
Performance Status	0	16 (53)
	1	14 (47)
	BRAF mutation (+)	8 (27)
	BRAF wild type	13 (43)
	Unknown	9 (30)
Recurrent disease after prior surgery		15 (50)
Presence of in-transit metastases		16 (53)
Prior adjuvant HDI		5 (17)
Estimated risk Stage	IIIB	3 (10)
	IIIC	25 (83)
	*IV (Not eligible)	2 (7)

HDI: High-dose interferon-a; ECOG: Eastern Cooperative Oncology Group
